# No evidence of brown adipose tissue activation after 24 weeks of supervised exercise training in young sedentary adults in the ACTIBATE randomized controlled trial

**DOI:** 10.1038/s41467-022-32502-x

**Published:** 2022-09-12

**Authors:** Borja Martinez-Tellez, Guillermo Sanchez-Delgado, Francisco M. Acosta, Juan M. A. Alcantara, Francisco J. Amaro-Gahete, Wendy D. Martinez-Avila, Elisa Merchan-Ramirez, Victoria Muñoz-Hernandez, Francisco J. Osuna-Prieto, Lucas Jurado-Fasoli, Huiwen Xu, Lourdes Ortiz-Alvarez, María J. Arias-Tellez, Andrea Mendez-Gutierrez, Idoia Labayen, Francisco B. Ortega, Milena Schönke, Patrick C. N. Rensen, Concepción M. Aguilera, José M. Llamas-Elvira, Ángel Gil, Jonatan R. Ruiz

**Affiliations:** 1 PROFITH (PROmoting FITness and Health through Physical Activity) Research Group, Department of Physical Education and Sport, Faculty of Sport Sciences, University of Granada, Granada, Spain; 2 Department of Medicine, Division of Endocrinology and Einthoven Laboratory for Experimental Vascular Medicine, Leiden University Medical Center, Leiden, The Netherlands; 3 Department of Education, Faculty of Education Sciences and SPORT Research Group (CTS-1024), CERNEP Research Center, University of Almería, Almería, Spain; 4 Pennington Biomedical Research Center, Baton Rouge, LA USA; 5 Turku PET Centre, University of Turku, Turku, Finland; 6 Turku PET Centre, Turku University Hospital, Turku, Finland; 7 InFLAMES Research Flagship Center, University of Turku, Turku, Finland; 8 EFFECTS-262 Research Group, Department of Physiology, School of Medicine, University of Granada, Granada, Spain; 9 Department of Analytical Chemistry, University of Granada, Granada, Spain; 10 Research and Development of Functional Food Center (CIDAF), Granada, Spain; 11 Department of Biochemistry and Molecular Biology II, “José Mataix Verdú” Institute of Nutrition and Food Technology (INYTA), Biomedical Research Center (CIBM), University of Granada, Granada, Spain; 12 Department of Nutrition, Faculty of Medicine, University of Chile, Independence, 1027 Santiago, Chile; 13 Instituto de Investigación Biosanitaria, ibs.Granada, Granada, Spain; 14 CIBER Fisiopatología de la Obesidad y la Nutrición (CIBEROBN), Madrid, Spain; 15 Institute for Innovation & Sustainable Development in Food Chain (IS-FOOD), Public University of Navarra, Campus de Arrosadía, 31008 Pamplona, Spain; 16 Faculty of Sport and Health Sciences, University of Jyväskylä, Jyväskylä, Finland; 17 Nuclear Medicine Service, Virgen de las Nieves University Hospital, Granada, Spain; 18 Nuclear Medicine Department, Biohealth Research Institute in Granada, Granada, Spain

**Keywords:** Obesity, Fat metabolism

## Abstract

Exercise modulates both brown adipose tissue (BAT) metabolism and white adipose tissue (WAT) browning in murine models. Whether this is true in humans, however, has remained unknown. An unblinded randomized controlled trial (ClinicalTrials.gov ID: NCT02365129) was therefore conducted to study the effects of a 24-week supervised exercise intervention, combining endurance and resistance training, on BAT volume and activity (primary outcome). The study was carried out in the Sport and Health University Research Institute and the Virgen de las Nieves University Hospital of the University of Granada (Spain). One hundred and forty-five young sedentary adults were assigned to either (i) a control group (no exercise, *n* = 54), (ii) a moderate intensity exercise group (MOD-EX, n = 48), or (iii) a vigorous intensity exercise group (VIG-EX *n* = 43) by unrestricted randomization. No relevant adverse events were recorded. 97 participants (34 men, 63 women) were included in the final analysis (Control; *n* = 35, MOD-EX; *n* = 31, and VIG-EX; *n* = 31). We observed no changes in BAT volume (Δ Control: −22.2 ± 52.6 ml; Δ MOD-EX: −15.5 ± 62.1 ml, Δ VIG-EX: −6.8 ± 66.4 ml; P = 0.771) or ^18^F-fluorodeoxyglucose uptake (SUVpeak Δ Control: −2.6 ± 3.1 ml; Δ MOD-EX: −1.2 ± 4.8, Δ VIG-EX: −2.2 ± 5.1; *p* = 0.476) in either the control or the exercise groups. Thus, we did not find any evidence of an exercise-induced change on BAT volume or activity in young sedentary adults.

## Abstract (web summary)

Exercise modulates brown adipose tissue (BAT) metabolism in murine models. Here the authors report that there is no evidence that 24 weeks of supervised exercise training modulates BAT volume or function in young sedentary adults in the ACTIBATE randomized controlled trial.

## Introduction

Brown adipose tissue (BAT) is a mammalian thermogenic tissue, characterized by its ability to dissipate energy as heat via the action of uncoupling protein 1 (UCP1). This is mainly triggered by the activation of the sympathetic nervous system in response to cold^1–4^. In the late 2000s, several independent research groups showed BAT to be present and metabolically active in human adults^5–9^. Moreover, the presence of beige adipocytes, a brown-like type of adipocyte that expresses UCP1 and shows non-shivering thermogenic capacity^10^, was documented within white adipose tissue (WAT) depots in humans^11^. Beige adipocyte proliferation and activity can be increased through a process known as WAT browning^12^.

Over the last decade, BAT and beige adipocyte recruitment and activation have been thought potential targets for combating obesity and its comorbidities^13^. Initially, it was hypothesized that BAT might have a profound impact on human daily energy expenditure, and might therefore be enlisted to help reduce adiposity^14^ and improve cardiometabolic health^15,16^. Although this was found to be true for mice^4,17,18^, BAT seems to play only a minor role in human daily energy expenditure^3,19^. BAT is known, however, to secrete several endocrine signaling molecules that might orchestrate favorable cardiometabolic effects^20–22^. Recruiting or activating BAT and WAT browning might still have cardiometabolic health benefits. Mouse studies have shown that exercise induces WAT browning^23,24^, and exercise might thus be speculated to do the same in humans^25^. Indeed, exercise stimulates the secretion of many endocrine factors that increase BAT metabolism and WAT browning^26^. However, exercise also increases heat production, which might downregulate the thermogenic activity of BAT^27^. Unfortunately, little information is available regarding the effect of exercise on human BAT, and what there is, is controversial^28,29^.

In the present work, it was hypothesized that exercise training in young sedentary adults increases BAT volume and glucose uptake (as measured by ^18^F-fluorodeoxyglucose [^18^F-FDG] uptake) in an intensity-dependent manner (i.e., the greater the exercise intensity, the stronger the BAT responses). A randomized controlled trial was therefore undertaken to investigate the effect of a 24-week supervised exercise, intervention combining endurance and resistance training (moderate or vigorous intensity) on BAT volume, ^18^F-FDG uptake, and mean radiodensity in young sedentary adults. Here, we show no evidence that BAT volume, ^18^F-FDG uptake or mean radiodensity levels are modified in response to the exercise training intervention. While exercise reduces adiposity (−11% [mean for both exercise intensity groups]), and improves muscular (+15%) and cardiorespiratory fitness (+11%), these changes are not correlated with initial BAT volume, ^18^F-FDG uptake or mean radiodensity, nor any changes therein.

## Results and discussion

145 young, sedentary adults were randomly assigned to either: (i) a control group (no exercise, hereinafter CON), (ii) a moderate-intensity exercise group (hereinafter MOD-EX), or (iii) a vigorous-intensity exercise group (hereinafter VIG-EX). Both exercise groups performed 150 min/week of endurance training, distributed over 3–4 sessions/week, plus 80 min per week of resistance exercise over two sessions/week (Supplementary Fig. S1). The primary endpoints were BAT volume, ^18^F-FDG uptake and mean radiodensity, as measured by a static ^18^F-FDG positron emission tomography/computed tomography (PET/CT) scan following 2 h of personalized cold exposure. The secondary endpoints included body weight and composition, cardiometabolic risk factors (i.e., blood pressure, fasting serum glucose, insulin, triacylglycerols, cholesterol, liver enzymes and C-reactive protein), and physical fitness (i.e., muscular strength and cardiorespiratory capacity).

### Study participants

**Fig. 1:**
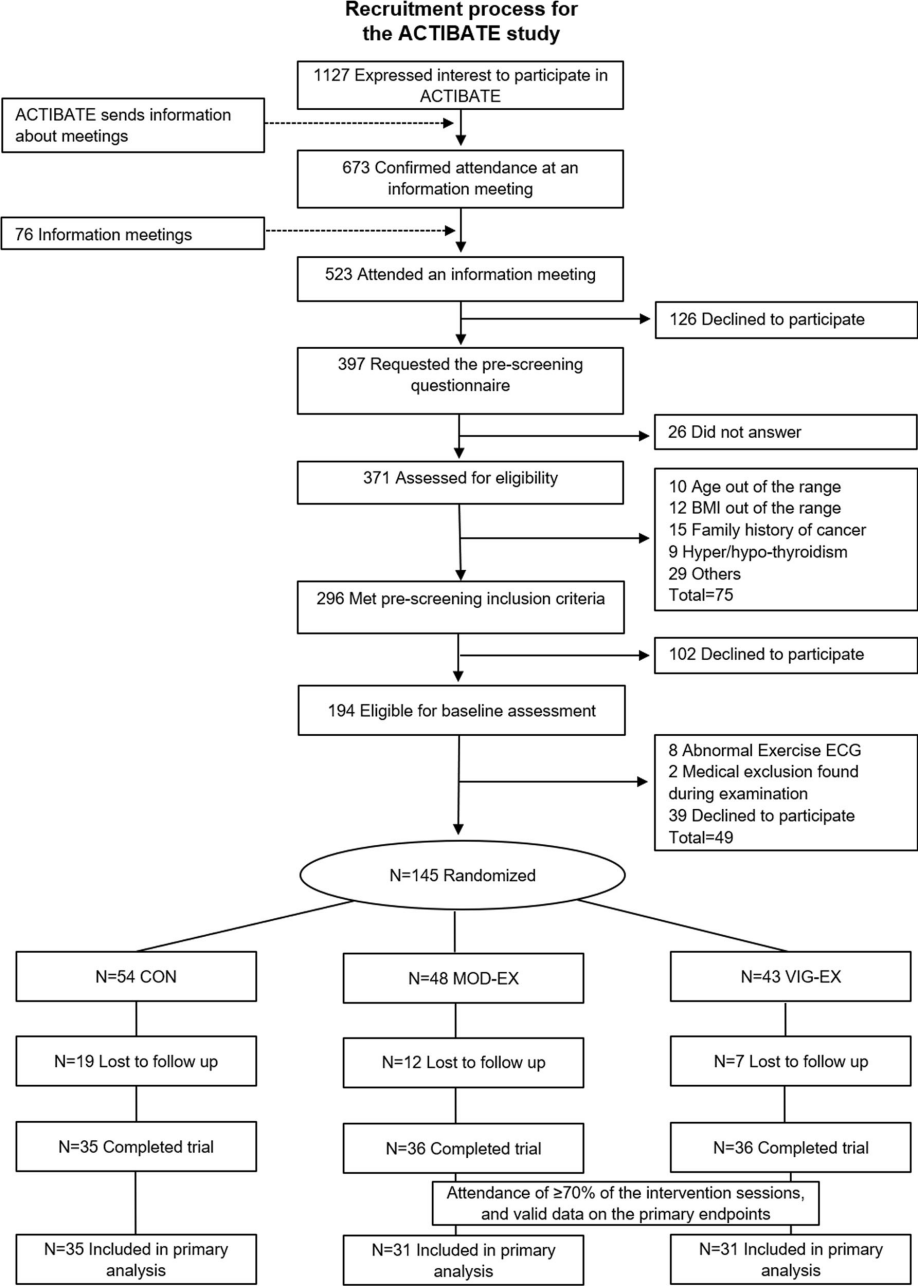
Participant enrollment in the ACTIBATE study. BMI body mass index, CON control group, MOD-EX moderate-intensity exercise group, VIG-EX vigorous-intensity exercise group, ECG electrocardiogram.

Out of 1127 initially interested individuals, 523 attended one of 76 scheduled information meetings. 126 of the latter individuals declined to participate, and 397 completed a pre-screening survey. Among these, 296 were eligible for screening (Fig. 1). When contacted to schedule their screening visit, 102 declined to participate. The remaining 194 participants provided informed consent to be included in the study, and began baseline assessment; however, 49 did not finish this phase (39 declined to participate, 8 had an abnormal exercise electrocardiogram, and 2 met a medical exclusion criterion). The first participant was enrolled on October 5^th^, 2015 and the last participant was enrolled on November 7th, 2016. Thus, 145 individuals were finally randomized into the three experimental groups. During the 24-week intervention period, another 38 participants dropped out of the study; 107 participants therefore completed it. Among these, three participants from the MOD-EX group, and two participants from the VIG-EX group, were excluded from the main analyses for attending <70% of the training sessions. Additionally, five participants (MOD-EX; *n* = 2 and VIG-EX; *n* = 3) were excluded from the main analyses due to problems in collecting the primary outcome data (the cooling vest or chiller involved suffered technical problems during testing, or the ^18^F-FDG tracer was improperly injected). The baseline characteristics of the remaining 97 participants (65% women, CON; n = 35, MOD-EX; *n* = 31, and VIG-EX; *n* = 31) were similar across the three experimental groups (all *P* ≥ 0.059, Table 1 and Supplementary Table S1).

**Table 1:** Baseline characteristics of the study participants

	CON (*n* = 35)		MOD-EX (*n* = 31)		VIG-EX (*n* = 31)	
	Mean	SD	Mean	SD	Mean	SD
Demographics						
Age (years old)	22.0	2.0	22.0	2.1	22.0	2.5
Male (*n*, %)	15	42.8	9	29.1	10	32.2
Female (*n*, %)	20	57.2	22	70.9	21	67.8
Brown adipose tissue outcomes						
BAT volume (mL)	74.6	52.4	68.9	62.9	64.0	59.1
BAT SUVmean	2.2	1.0	2.1	1.2	2.2	1.1
BAT SUVpeak	6.7	4.1	6.1	5.0	6.6	5.3
BAT mean radiodensity (HU)	−60.5	8.9	−60.3	9.0	−58.0	11.0
Body composition outcomes						
BMI (kg/m^2^)	24.5	4.4	24.9	4.2	25.2	4.5
Overweight (*n*, %)	9	26	9	28	13	41
Obesity (*n*, %)	5	14	4	12	4	12
Lean mass (kg)	42.6	10.7	40.8	7.9	42.5	9.6
Fat mass (kg)	23.6	7.8	25.3	9.1	26.1	8.1
Fat mass (%)	34.3	7.2	36.4	8.7	36.4	6.9
VAT mass (g)	333	172	351	181	365	190
Cardiometabolic risk factors						
Glucose (mg/dL)	87.6	6.6	87.8	7.1	86.7	5.8
Insulin (µIU/mL)	8.2	5.4	8.3	4.5	8.6	4.2
HOMA-IR	1.8	1.4	1.8	1.2	1.9	1.0
γ-GT (IU/L)	22.5	28.1	15.6	8.4	19.1	9.7
ALT (IU/L)	24.4	32.4	15.5	6.1	17.6	8.9
ALP (IU/L)	71.9	22.8	72.4	18.4	74.1	16.5
Cholesterol (mg/dL)	154.8	27.8	163.8	31.1	170.4	33.2
HDL-C (mg/dL)	53.0	10.0	52.6	11.3	51.7	13.9
LDL-C (mg/dL)	86.2	22.9	95.9	28.5	100.2	24.6
Triacylglycerols (mg/dL)	78.5	44.0	87.1	63.0	93.2	48.8
C-reactive protein (mg/L)	2.2	2.3	3.5	5.2	2.2	2.6
Systolic blood pressure (mmHg)	116.9	12.6	117.8	10.6	116.6	11.4
Diastolic blood pressure (mmHg)	69.5	7.8	72.2	5.9	72.0	8.0
Physical fitness outcomes						
Handgrip strength (kg)	32.3	7.1	30.6	7.8	31.2	7.7
RM leg press (kg)	212.5	75.0	190.7	56.9	200.6	68.6
RM bench press (kg)	34.4	16.5	28.6	10.7	30.9	13.2
Time to exhaustion (s)	883	234	942	192	953	180
VO_2_peak (mL/kg/min)	42.0	9.2	40.7	6.7	41.0	7.9
VO_2_peak (mL/min)	2955	858	2817	543	2971	866

Data are presented as means and standard deviations (SD) unless otherwise stated. Serum and plasma concentrations were log_10_ transformed for statistical analyses. BAT mean radiodensity sample size: CON *n* = 25; MOD-EX *n* = 17; VIG-EX *n* = 16. BAT SUVmean and SUVpeak are shown relative to lean mass.

*γ-GT* gamma-galactosyltransferase, *ALP* alkaline phosphatase, *ALT* alanine aminotransferase, *BAT* brown adipose tissue, *BMI* body mass index, *CON* control group, *CRP* C-reactive protein, *HDL-C* high-density lipoprotein cholesterol, *HOMA-IR* homeostatic model assessment for insulin resistance, *HU* Hounsfield Units, *LDL-C* low-density lipoprotein cholesterol, *MOD-EX* moderate-intensity exercise group, *VIG-EX* vigorous-intensity exercise group, *RM* repetition maximum, *SUV* standardized uptake value, *VAT* visceral adipose tissue, *VO*_*2*_ oxygen consumption.

### No evidence of brown adipose tissue volume, ^18^F-fluorodeoxyglucose uptake, or mean radiodensity changes after 24 weeks of supervised exercise training

**Fig. 2:**
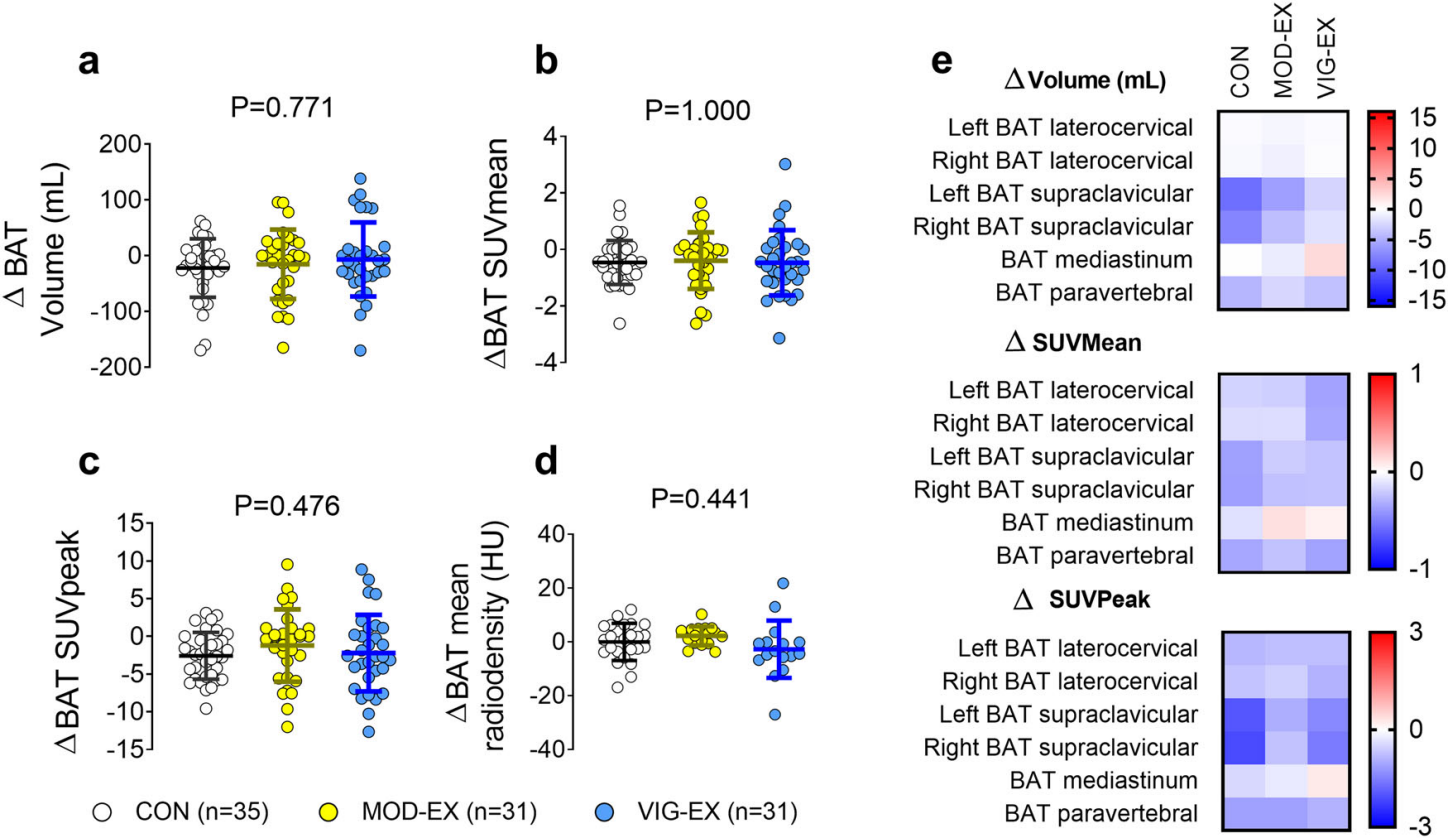
Effect of the 24-week supervised exercise intervention on brown adipose tissue (BAT) volume, ^18^F-fluorodeoxyglucose (^18^F-FDG) uptake (Standardized uptake Value [SUV] mean and peak), and mean radiodensity. **a** Total BAT volume. **b** Total BAT SUVmean. **c** Total BAT SUVpeak. **d** Total BAT mean radiodensity (CON *n* = 25; MOD-EX *n* = 17; VIG-EX *n* = 16). **E** Regional BAT volume, SUVmean, and SUVpeak. SUV values are shown relative to lean mass. Δ was calculated as post-intervention minus baseline values for every outcome. *P* values are from analyses of covariance adjusting for baseline values (*n* = 97). In panel **e**, all *P* values ≥0.1. CON control group, HU Hounsfield Units, MOD-EX Moderate-intensity exercise group, VIG-EX Vigorous-intensity exercise group. Bars represent mean and standard deviation. Source data are provided as a Source Data file.

Neither the MOD-EX nor VIG-EX regimen significantly altered BAT volume (Δ [post-baseline values]: Δ CON = −22.2 ± 52.6 mL (mean ± standard deviation); Δ MOD-EX = −15.5 ± 62.1 mL; Δ VIG-EX = −6.8 ± 66.4 mL; *p* = 0.771, Fig. 2a). Nor did they alter ^18^F-FDG uptake (standardized uptake value [SUV] mean: Δ CON = −0.4 ± 0.7; Δ MOD-EX = −0.4 ± 1.0; Δ VIG-EX = −0.5 ± 1.1; *P* = 1.000, Fig. 2b; SUVpeak: Δ CON = −2.6 ± 3.1; Δ MOD-EX = −1.2 ± 4.8; Δ VIG-EX = −2.2 ± 5.1: *P* = 0.476, Fig. 2c). In addition, neither BAT volume nor ^18^F-FDG uptake in different BAT depots (i.e., laterocervical, supraclavicular, mediastinum or paravertebral areas; all *P* > 0.1, Fig. 2e) were significantly modified. BAT mean radiodensity (a proxy of fat content^30^ also remained unchanged (Δ CON = −0.04 ± 6.91 Hounsfield Units [HU]; Δ MOD-EX = 2.2 ± 3.5 HU; Δ VIG-EX = −2.7 ± 10.7 HU; *P* = 0.441, Fig. 2d). We repeated the analyses using mixed-effects regression models and the absence of evidence of BAT activation after supervised exercise training persisted (Supplementary Fig. S2).

Previous clinical studies have shown that BAT volume and ^18^F-FDG uptake in response to mild-cold and thermoneutral exposures are different in women and men^31,32^, which was also confirmed in this cohort^33^. However, the absence of evidence of BAT activation after exercise training was recorded for both sexes when analyzed separately (all *P* > 0.7). Only participants who attended ≥70% of the training sessions were included in the main analyses (Supplementary Fig. S3), although the results did not change when sensitivity analyses were performed including participants whose attendance was <70% and ≥85%.

Following the recommendations of the *Brown Adipose Reporting Criteria in Imaging STudies* (BARCIST 1.0^34^) report, BAT was defined as those imaging voxels with a radiodensity between −190 and −10 HU, and a SUV higher than an individualized threshold (1.2/[lean mass/body mass]). Both our research group and others have reported there to be inter-study heterogeneity regarding the criteria used for BAT quantification in ^18^F-FDG PET/CT scans (i.e., radiodensity range and SUV threshold)^35–37^. Our group also showed that the most frequently used criteria for measuring BAT provide non-comparable estimates of BAT volume and ^18^F-FDG uptake^35^. Hence, the use of different criteria might sometimes allow the effect of an intervention to be detected. Accordingly, in the present work, sensitivity analyses were performed using alternative criteria to quantify BAT (i.e., radiodensity: −250/−50 HU and SUV threshold >2.0; and radiodensity: −180/−10 HU and SUV threshold > 1.5). The lack of evidence of BAT activation persisted (all *P* > 0.5).

**Fig. 3:**
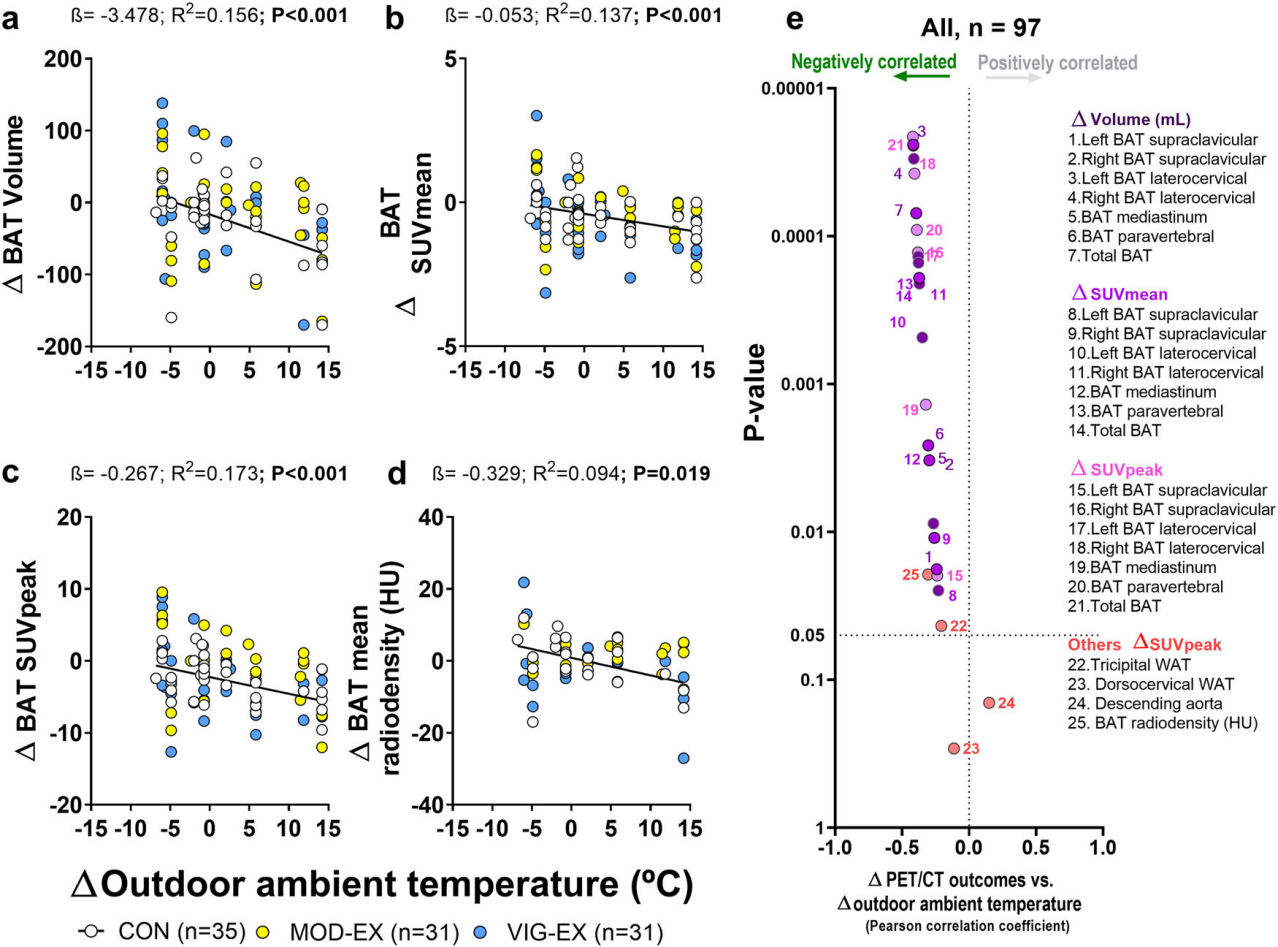
Associations between the Δ outdoor ambient temperature and Δ (post-intervention minus baseline value) brown adipose tissue (BAT)-related outcomes. **a** Total BAT volume. **b** Total BAT standardized uptake value (SUV) mean. **c** Total BAT SUVpeak. **d** Total BAT mean radiodensity (CON *n* = 25; MOD-EX *n* = 17; VIG-EX *n* = 16). **e** Regional BAT volume, SUVmean, and SUVpeak. SUV values are shown relative to lean mass. *P* and *β* values are obtained from linear regression analyses. β non-standardized coefficients, BAT brown adipose tissue, CON Control group, HU Hounsfield units, MOD-EX moderate-intensity exercise group, *R*^2^ explained variance, SUV standardized uptake value, VIG-EX vigorous-intensity exercise group, WAT white adipose tissue.

In agreement with the present results, a previous study^29^ showed that six weeks of moderate resistance training did not modify BAT ^18^F-FDG uptake, although a slight reduction in BAT volume was seen in the laterocervical region in 11 men. However, the latter study did not include a control group, limiting the conclusions that can be drawn. BAT volume and ^18^F-FDG uptake are strongly influenced by outdoor temperature, with activation greater in colder months^38–47^; therefore, not including a control group leaves it impossible to know if any changes in BAT volume and/or ^18^F-FDG uptake were the result of seasonal temperature variation or a consequence of the intervention. The present results clearly support this notion, since the Δ outdoor ambient temperature was negatively associated with Δ BAT volume, Δ ^18^F-FDG uptake and Δ BAT mean radiodensity (all *R*^2^ > 0.09 and *P* < 0.001, Fig. 3a–d) in all three groups. These correlations remained unaltered when BAT-related outcomes were calculated for individual BAT depots separately (all *r* ≤ −0.207 and *P* ≤ 0.043, Fig. 3e). At baseline, we found that outdoor ambient temperature at which the PET/CT scans were performed negatively correlated with BAT-related outcomes (all *r* ≥ −0.364 and *P* < 0.001, Supplementary Table S2), although these significant correlations disappeared when post-intervention values were used instead (Supplementary Table S2). After observing these correlations, we performed the main analyses adding the outdoor ambient temperature as covariate and the results did not change (all *P* ≥ 0.182; Supplementary Table S3). In addition, we found weak to moderate correlations (range from *r* = 0.515 to *r* = 0.872) between baseline and post-intervention BAT-related outcomes, which suggests that our protocol is reproducible despite the presence of large seasonal variation (Supplementary Table S4).

The present BAT assessments were made after a personalized 2 h cold exposure, i.e., based on the participants’ shivering threshold - a proxy of cold tolerance^16^. While this method is widely used^15,33,34^ it has been suggested that it might not be entirely adequate. Shivering patterns are variable among individuals, making the detection of shivering onset difficult to be determined objectively^48,49^. Moreover, exercise has been reported to increase cold tolerance^50^, and could therefore alter the detection of shivering onset during post-intervention personalized cold exposure, introducing a bias regarding the effect of exercise on BAT recruitment/activation. In the present work, however, the shivering threshold (assessed via the cooling vest water temperature at the onset of shivering [see Methods]) was not modified in any of the groups after the intervention (*P* = 0.154, Supplementary Table S1).

The lack of evidence of BAT volume, ^18^F-FDG uptake or mean radiodensity modification after exercise training is in line with the results of cross-sectional studies that show no association of BAT-related outcomes with objectively measured free-living physical activity^51^ or muscular or cardiorespiratory fitness^52^. Case-control studies have revealed, however, that endurance-trained athletes show lower BAT volumes and ^18^F-FDG uptakes than their sedentary counterparts^53,54^. It is thus conceivable that the high volume of exercise training undertaken by endurance-trained athletes reduces BAT volume and ^18^F-FDG uptake as an adaptation to high activity energy expenditure levels, while the more moderate exercise training regimens implemented in the present study do not. This hypothesis concurs with the constrained total energy expenditure model, which suggests that low levels of physical activity do not inhibit energy-consuming physiological processes such as BAT thermogenesis^55^, whereas higher levels of physical activity do in order to maintain total daily energy expenditure within a homeostatic range^56,57^.

Although ^18^F-FDG PET/CT scanning is still the most widely used technique for the in vivo assessment of human BAT, the use of ^18^F-FDG has important limitations^58^. Cold exposure increases BAT ^18^F-FDG uptake, but the amount of ^18^F-FDG taken up does not necessarily mimic the BAT’s thermogenic activity, since most glucose taken up by BAT does not fuel mitochondrial oxidative capacity^59,60^. Accordingly, insulin infusions increase human BAT ^18^F-FDG uptake without increasing BAT perfusion, suggesting that the increase in ^18^F-FDG uptake is not paralleled by increased BAT thermogenic activity^61^. ^18^F-FDG is taken up by BAT through insulin-independent (glucose transporter 1, GLUT-1) and insulin-dependent (GLUT-4) mechanisms^62^. Consequently, BAT insulin sensitivity is likely to partially bias the assessment of BAT volume and activity, when performing ^18^F-FDG uptake quantification^63^. Any intervention that changes BAT insulin sensitivity might therefore result in a different ^18^F-FDG uptake, regardless of the change in BAT thermogenic activity. Indeed, exercise is known to increase insulin sensitivity in many peripheral tissues^64^, so it is plausible that it modifies BAT insulin sensitivity too. Moreover, exercise training increases mitochondrial oxidative capacity in skeletal muscle^65^, and it might likewise increase BAT oxidative metabolism rather than BAT ^18^F-FDG uptake. BAT oxidative metabolism, as well as the uptake of fatty acids in human BAT, has been recently quantified by ^11^C-acetate, ^15^O-oxygen and ^18^F-fluoro-thiaheptadecanoic acid (^18^FTHA) PET in response to cold exposure in humans^48,66–68^. Thus, it would be interesting to ascertain in future studies if assessing BAT metabolism by methods other than glucose uptake would unveil whether exercise can activate/recruit BAT.

Even if a better PET radiotracer were available, PET/CT imaging would still be affected by important limitations when evaluating the effect of exercise on BAT recruitment/activation. It has been suggested that the human BAT detectable by PET/CT scanning in the laterocervical and supraclavicular areas is composed of a combination of brown and beige-like adipocytes^69–73^. Most preclinical studies have shown that exercise induces WAT browning^23,24^, although these results might be just applicable to room temperature environments (i.e., below thermoneutrality^74,75^). Thus, exercise might not increase the volume or activity of the brown-like depots detectable by PET/CT scanning, but may still induce the browning of small clusters of adipocytes, that are not detected by this technique. If this is the case, the limited resolution of PET imaging (usually 4 mm) would not allow the appearance of beige cells within the WAT to be captured^58^. The effect of exercise on BAT volume/activity and WAT browning needs to be further explored as better methods become available.

The present finding that exercise seems not to alter BAT volume nor activity in human adults should also be tested in new studies involving different types of exercise program and populations. It is well known that different exercise types (endurance vs. resistance vs. a combination of both) and intensities induce different physiological adaptations^76^. The present exercise regimens combined resistance and endurance exercises, but it cannot be determined from the results whether the resistance or endurance components on their own might have different effects on BAT recruitment/activation. Interestingly, we found that outdoor ambient temperature influenced BAT volume, ^18^F-FDG uptake and radiodensity, which might be masking the possible effect of exercise on BAT recruitment/activity. Thus, other study designs and experimental settings, with longer durations, and taking into account the potential effect of outdoor ambient temperature, should be implemented for addressing this scientific question. Finally, BAT presence and activity are reported to be blunted by aging^6,13,77–79^ and metabolic disease^46^. Since the present work involved relatively healthy young sedentary adults, the findings made may not apply to older or metabolically compromised individuals.

### Twenty-four weeks of supervised exercise training reduces adiposity and increases muscular and cardiorespiratory fitness

**Fig. 4:**
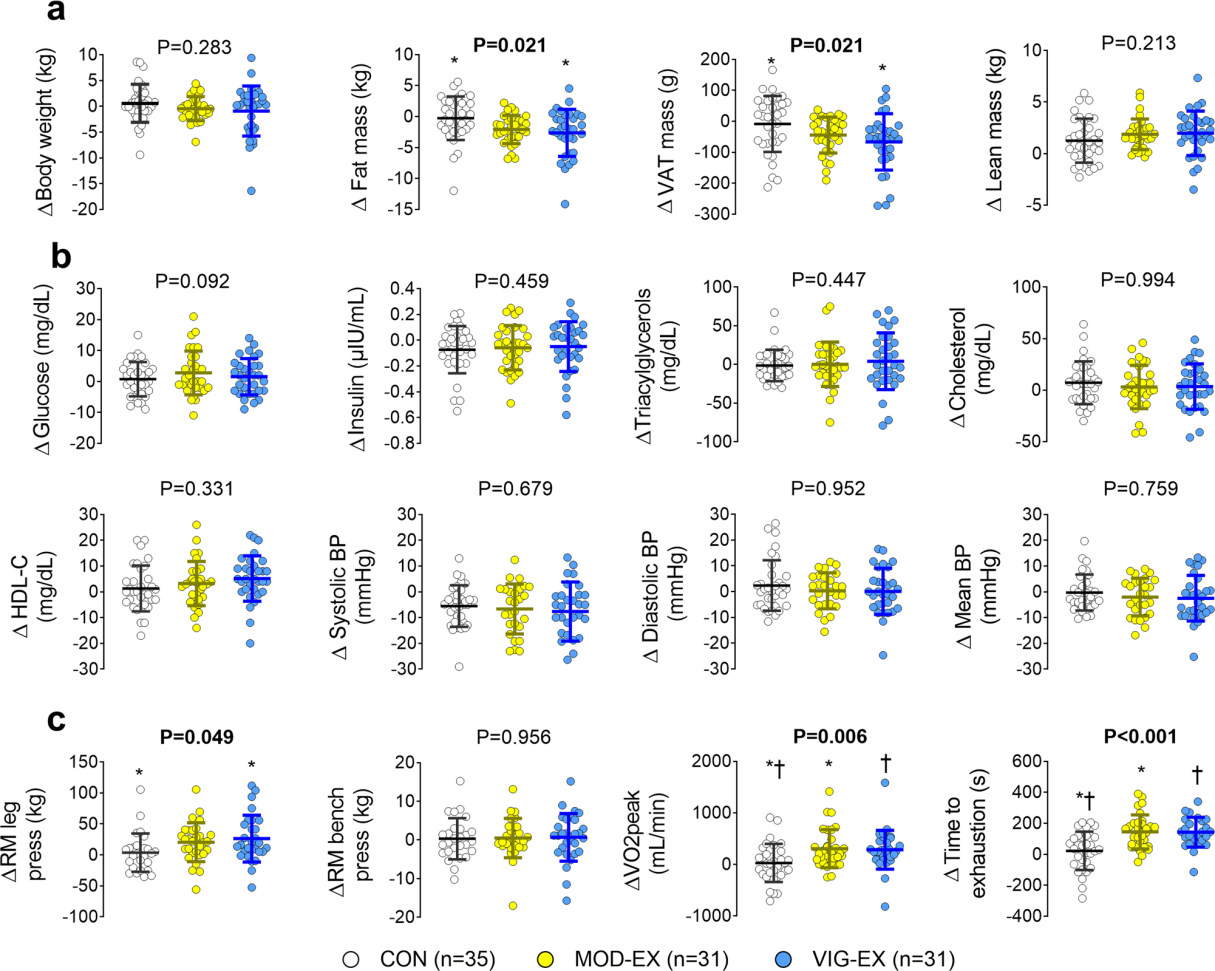
Effect of the 24-week supervised exercise intervention on secondary endpoints. **a** Body weight and composition parameters, **b** cardiometabolic risk factors, and **c** physical fitness parameters. Δ was calculated as post-intervention minus baseline value for every outcome. Serum concentrations were log_10_ transformed. P values are from analyses of covariance (ANCOVAs), adjusting for baseline values. * and † indicate significant differences between pairs after Bonferroni correction. BP Blood pressure, CON Control group, HDL-C high-density lipoprotein cholesterol, MOD-EX Moderate-intensity exercise group, RM repetition maximum, VAT visceral adipose tissue, VO_2_ oxygen consumption, VIG-EX vigorous-intensity exercise group. Bars represent the mean and standard deviation. Source data are provided as a Source Data file.

Exercise reduced both total body fat (Δ CON = −0.3 ± 3.5 kg; Δ MOD-EX = −2.1 ± 2.3 kg; Δ VIG-EX = −2.7 ± 3.8 kg; *P* = 0.021) and visceral fat mass (Δ CON = −8.6 ± 90.3 g; Δ MOD-EX = −44.5 ± 57.9 g; Δ VIG-EX = −66.7 ± 91.3 g; *P* = 0.021), although post-hoc comparisons detected differences only between the CON and VIG-EX groups (Fig. 4a and Supplementary Table S1). As expected, exercise tended to increase lean mass in both intervention groups, although only a trend was seen with respect to the control group (*P* = 0.213, Fig. 4a and Supplementary Table S1). No evidence of a reduction in body weight (*P* = 0.283, Fig. 4a), or improvements on blood pressure, fasting serum concentrations of glucose, insulin, liver enzymes (i.e., γ-GT, ALT and ALP), cholesterol (i.e., HDL-C and LDL-C), triacylglycerols, and C-reactive protein (all *P* ≥ 0.092, Fig. 4b and Supplementary Table S1) after the exercise intervention were observed. These findings concur with those of previous exercise intervention studies in relatively healthy individuals^80,81^. The lack of evidence of an improvement in cardiometabolic risk factors after exercise training may be related to the young age (22 ± 2 years old) and the relatively healthy status of the present participants, whose cardiometabolic risk markers were usually within normal ranges.

The intervention enhanced lower body muscular strength as measured via the repetition maximum (RM) leg press test (Δ CON = 3.7 ± 30.8 kg; Δ MOD-EX = 20.5 ± 31.1 kg; Δ VIG-EX = 26.3 ± 37.7 kg; *P* = 0.049, Fig. 4c, and Supplementary Table S1), whereas a lack of effect was observed on RM bench press or handgrip strength results after the exercise intervention (all *P* ≥ 0.993, Supplementary Table S1). In addition, the intervention increased the time to exhaustion in a maximum effort test (Δ CON = 21.8 ± 124.0 s; Δ MOD-EX = 144.2 ± 109.8 sec; Δ VIG-EX = 142.2 ± 96.2 s; *P* < 0.001, Fig. 4c) and VO_2_peak (Δ CON = 28 ± 371 mL/min; Δ MOD-EX = 305 ± 367 mL/min; Δ VIG-EX = 285 ± 376 mL/min; *P* = 0.006, Fig. 4c and Supplementary Table S1), although no significant differences were seen between the exercise groups. Only those participants who attended ≥70% of the total training sessions were included in the main analyses (Supplementary Fig. S3), but the results remained unaltered when other attendance criteria were taken into account (<70%, or ≥85%).

### Exercise-induced changes in adiposity, muscular, and cardiorespiratory fitness do not correlate with any changes in brown adipose tissue

Preclinical studies have shown that, during exercise, BAT secretes signaling molecules that favor skeletal muscle function^82–84^. Thus, it may be hypothesized that individuals with higher BAT volumes might benefit to a greater extent from exercise. However, no significant correlations were detected between baseline values, or any change in BAT-related outcomes, and any exercise-induced changes in body composition, cardiometabolic risk profile, muscular strength or cardiorespiratory capacity (all *P* > 0.1, Supplementary Table S5). Although significant correlations were found between changes in total plasma cholesterol and BAT-related outcomes in the CON and MOD-EX groups, these did not persist after false discovery rate correction (all *q* values ≥0.54; Supplementary Table S5). These data agree with the findings of a previous study in which no relationship was seen between exercise-induced reduction in adiposity or improvement in the lipoprotein profile, and exercise-induced changes in BAT ^18^F-FDG uptake^29^. These observations suggest that the cardiometabolic improvements induced by the exercise intervention were not mediated by BAT activation, at least as quantified by ^18^F-FDG PET-CT.

In conclusion, there was no evidence of changes in BAT-related outcomes after 24-week supervised exercise intervention combining resistance and endurance training at different intensities in young sedentary adults. The exercise intervention reduced adiposity and enhanced muscular and cardiorespiratory fitness to a comparable extent in both exercise groups, but seemed not to modify other cardiometabolic risk factors. These exercise-induced changes in adiposity, muscular and cardiorespiratory fitness were not correlated with any individual changes in BAT-related outcomes, suggesting that the observed exercise-induced benefits are independent of BAT in such young sedentary adults.

## Methods

### Participant details

The study was approved by the Ethics Committee on Human Research of the University of Granada (no. 924) and by the *Servicio Andaluz de Salud* (*Centro de Granada, CEI*-Granada, Spain). All participants provided informed consent in order to be included. A total of 145 young sedentary adults aged 18–25 years old participated in the *Activating Brown Adipose Tissue Through Exercise* (ACTIBATE) study (ClinicalTrials.gov ID: NCT02365129; Fig. 1)^85^. For participant recruitment, the study was advertised through social networks, local media, and via posters. The inclusion criteria included self-reported sedentary lifestyle (performing a maximum of 20 min of moderate-to-vigorous physical activity per day on less than three days per week), being a non-smoker, not taking any medication, and having a stable body weight over the last three months. The exclusion criteria were: having been diagnosed with diabetes, hypertension, or any other significant medical condition (either life-threatening or that might interfere with or be aggravated by exercise), being pregnant, using medication deemed to affect energy metabolism, or to be frequently exposed to cold temperatures^85^.

### Study design

After baseline examination, participants were assigned to one of three groups via computer-generated simple unrestricted randomization^86^: (i) a control group (no exercise, CON), (ii) a moderate-intensity exercise group (MOD-EX), and (iii) a vigorous-intensity exercise group (VIG-EX). The study was conducted over two consecutive years (from September 2015 to June 2016, and from September 2016 to June 2017). In both years, participants were enrolled over four waves (16–24 participants each) starting between September and December. The first participant was enrolled on October 5th, 2015 and the last participant was enrolled on November 7th, 2016. All outcome assessments were made at the same time of day before and after the 24 weeks intervention. All participants were instructed not to modify their normal routine or their physical activity or dietary pattern over the study period.

### Supervised exercise training program

A detailed description of the supervised exercise training program used in this work, which combined resistance and endurance training as recommended by World Health Organization (WHO) guidelines^87^, has been previously published^82^. For 24 weeks, participants trained 3–4 times per week, completing a total of 150 min/week of endurance exercise (performed in all sessions) and 80 min/week of resistance exercise (performed over 2 sessions/week) at the same training center. Both resistance and endurance training were individualized to each participant’s physical fitness. The MOD-EX group performed resistance training at 50% of the repetition maximum (RM), whereas the VIG-EX group trained with loads equivalent to 70% RM. The calculation of RM loads is described below and was adjusted monthly (Supplementary Fig. S1). All endurance training in the MOD-EX group was performed at 60% heart rate reserve (HRres), whereas in the VIG-EX group it was performed for 75 min/week at 60% HRres and 75 min/week at 80% HRres. HRres was calculated as the difference between the resting and maximum heart rates. The exercise intervention was organized in five phases of different duration, starting with a four-week familiarization phase (see detailed description Supplementary Fig. S1).

Participants exercised in groups of 10–12 people at the same time of day over the 24 weeks intervention (either 8.30–10.30 a.m., 4–6 p.m., 6–8 p.m., or 8–10 p.m). Attendance was registered via an electronic attendance sheet. Adherence to the prescribed intensity of endurance training was quantified by heart rate monitors (RS800CX, Polar Electro Öy, Kempele, Finland).

### Primary endpoints: brown adipose tissue measurements

#### Shivering threshold test

For personalizing the cooling protocol used to activate BAT, the participants’ shivering threshold was determined 48–72 h before BAT volume and metabolism assessments. Participants first entered a warm room (22.1 ± 1.6 °C) where they rested in a sitting position for 30 min. They then entered in an air-conditioned room (19.8 ± 0.5 °C) and sat in a chair. After 15 min, they were asked to put on a temperature-controlled, water-perfused cooling vest covering the abdomen, chest, and supraclavicular region (Polar Products Inc., Stow, OH, USA). The water temperature was initially set at 16.6 °C and reduced slightly every 10 min until reaching 5.5 °C. If shivering was not detected at this point, the water temperature was decreased by 0.6 °C every 15 min until 3.8 °C was reached. If shivering did not occur, participants remained in the cold room for another 45 min (water at 3.8 °C). Shivering onset was visually detected by the researcher and confirmed by the participants themselves. The water temperature at shivering onset was deemed the shivering threshold and was used for determining the cooling vest water temperature during the personalized cooling protocol before the ^18^F-FDG-PET/CT scan (4 °C above the shivering threshold). If shivering did not occur, participants started the personalized cooling protocol before the ^18^F-FDG-PET/CT scan with the water temperature set at 3.8 °C.

#### Personalized cooling protocol and positron emission tomography/computed tomography scanning

Forty-eight to 72 h after the shivering threshold test, participants went to the *Hospital Virgen de las Nieves* in Granada (Spain) for the assessment of BAT volume, ^18^F-FDG uptake, and radiodensity. After resting in a warm room, they were placed in a cool room (19.5–20 °C) wearing the same cooling vest with the water temperature set at the personalized temperature, as explained above. After the first hour of cold exposure, the participants received an injection of ^18^F-FDG (~185 MBq) and the water temperature was increased by 1 °C to avoid shivering. The water temperature was also slightly increased at any time participants reported shivering. One hour later, they were subjected to ^18^F-FDG-PET/CT scanning in the supine position using a Siemens Biograph 16 PET/CT scanner (Siemens, Erlangen, Germany). Image acquisition covered an area of two BED positions from the atlas vertebra to thoracic vertebra 6, approximately. The natural calendar day when the baseline, and post-intervention PET/CT scan were performed was recorded from day 1 (January 1st) to day 365/366 (December 31^st^).

#### BAT volume, ^18^F-FDG uptake, and mean radiodensity

BAT volume, ^18^F-FDG uptake and BAT mean radiodensity were determined following current recommendations^34^ and using the Beth Israel plug-in for the FIJI program v.1^88^. Several regions of interest (ROIs) were semi-automatically outlined from the atlas vertebra to thoracic vertebra 4, on both the left and right sides of the body: laterocervical, supraclavicular, mediastinum, and paravertebral, which were later computed as a single ROI, as extensively described elsewhere^36,89^. BAT was defined as those voxels within the ROI showing: (i) a radiodensity between −190 and −10 HU; and (ii) a standardized uptake value (SUV) higher than an individualized threshold (1.2/[lean body mass/body mass])^34^. BAT volume was calculated as the sum of all voxels meeting the above-mentioned criteria. BAT ^18^F-FDG uptake was determined as the average SUV of all voxels meeting the above-mentioned criteria (SUVmean), and as the average of the three voxels showing the highest ^18^F-FDG uptake within 1 cm^3^ of the voxel with the highest ^18^F-FDG uptake among those meeting the criteria described above (SUVpeak). SUV values were expressed as a function of lean body mass^89^. BAT mean radiodensity was determined as the average radiodensity of those voxels meeting the aforementioned criteria in a single ROI covering the body (except the mouth) from the atlas to thoracic vertebra 4. Twenty-eight participants showed several voxels classified as BAT outside of the anatomical areas where BAT is located; their data were excluded from BAT mean radiodensity analyses. For sensitivity analyses, BAT volume, ^18^F-FDG uptake, and BAT mean radiodensity were also determined using other common criteria (i.e., radiodensity: −250/−50 HU and SUV threshold >2.0; and radiodensity: −180/−10 HU and SUV threshold >1.5)^35,36^.

#### Outdoor ambient temperature

We downloaded the outdoor ambient temperature from the Spanish National Meteorological Agency daily during the period of the study (from September 1, 2015 to July 1, 2017).

### Secondary endpoints

#### Anthropometrics and body composition

Body composition (i.e., fat, lean, and visceral adipose tissue mass) was measured by dual-energy X-ray absorptiometry scanning using a Hologic Discovery Wi device (Hologic Inc., Bedford, MA, USA). Participant height and weight (barefoot and wearing light clothing) were determined using a model 799 SECA scale and stadiometer (Electronic Column Scale, Hamburg, Germany). Body mass index (BMI) was calculated as body mass (kg) divided by height squared (m^2^).

#### Cardiometabolic risk factors

At 1–3 weeks before starting the exercise intervention, and at 3–4 days after the last exercise session, blood samples were drawn in the morning following an overnight fast. These samples were centrifuged and the serum aliquoted and stored at −80 °C for future analyses. Glucose, cholesterol, high-density lipoprotein cholesterol (HDL-C), and triacylglycerols were assessed using an AU5832 automated analyzer (Beckman Coulter Inc., Brea CA, USA). Low-density lipoprotein cholesterol (LDL-C) was estimated using the Friedewald formula^90^. Insulin was measured using the Access Ultrasensitive Insulin Chemiluminescent Immunoassay Kit (Beckman Coulter Inc., Brea, CA, USA). The homeostatic model assessment (HOMA) index was calculated as insulin (µU/mL) x glucose (mmol/L)/22.5. C-reactive protein was measured by immunoturbidimetric assay, employing the same automated analyzer as above. Liver enzyme (γ-GT, ALT, or ALP) concentrations were determined using a Beckman Coulter AU5832 analyzer and the appropriate Beckman Coulter reagent. Resting blood pressure was measured in the sitting position before and after exercise training on three non-consecutive days using an automatic Omrom M2 device (Omron Healthcare, Kyoto, Japan). Three measurements were made and the mean blood pressure calculated as *diastolic blood pressure* + 0.33 × (s*ystolic blood pressure*-*diastolic blood pressure*).

#### Physical fitness

Physical fitness was assessed in two different sessions, one focusing on muscular strength and the other on cardiorespiratory fitness. Before each session, participants fasted for 3–5 h, performed no vigorous exercise in the previous 48 h, nor moderate exercise in the previous 24 h, and did not consume caffeine-containing beverages on the day of the tests.

##### Muscular strength

Muscular strength was determined via the handgrip strength and RM leg and bench press tests. The handgrip strength test was performed using a Takei 5401 digital Grip-D hand dynamometer (Takei, Tokyo, Japan). Participants were asked to squeeze the grip gradually and continuously, until reaching maximum intensity. The test was performed twice with each hand, alternating between hands with a 1 min rest after each attempt. Men executed the test with the grip span of the dynamometer fixed at 5.5 cm, while it was adjusted to the hand size for women, according to a validated equation^91^. The maximum strength recorded in each hand was selected and the mean used in analyses.

Leg press RM was assessed using an A300/Model 2531 pneumatic leg press machine (Keiser Corporation, Fresno, CA, USA). To warm-up, participants first performed a set of 10 repetitions with a self-selected light weight. They then performed a set of eight repetitions using a load with which they could perform a 15-repetition maximum. After a 1 min recovery period, the resistance load was increased by the researcher, aiming to set a target load at which the participant could perform fewer than 10 repetitions (all participants were instructed to perform as many repetitions as they could). The test was stopped after 3–4 repetitions if the participants felt they could perform more than 10 repetitions with the resistance load set. A greater load was then selected, and the test repeated after resting for 5 min. No more than three attempts were allowed for assessing the RM. Later, following a similar procedure, each participant performed a bench press RM test using a bench within a Model 3111 pneumatic power rack (Keiser Corporation, Fresno, CA, USA). The RM was estimated by a standard equation^92^.

##### Cardiorespiratory fitness

Cardiorespiratory fitness (i.e., maximum oxygen consumption) was assessed by a maximum graded exercise test on a treadmill, (H/P/Cosmos Sports & Medical GmbH, Nussdorf-Traunstein, Germany), following the modified Balke protocol^93^. The warm-up consisted of walking at 3 km/h for 1 min, followed by 2 min at 4 km/h (0% slope). The incremental protocol started at a speed of 5.3 km/h and 0% slope. The slope was then increased by 1% every minute until the participants became exhausted (i.e., volitional exhaustion). Finally, participants went through a cooling-down period, walking at 4 km/h (0% slope) for 5 min. During the whole test, respiratory gas exchange (oxygen consumption [VO_2_] and carbon dioxide production [VCO_2_]) was recorded via indirect calorimetry using a CPX Ultima CardioO_2_ metabolic cart (Medical Graphics Corp, St. Paul, MN, USA), equipped with a model 7400 plastic facemask (Hans Rudolph Inc., Kansas City, MO, USA) and a preVent™ high metabolic flow sensor (Medical graphics Corp, St. Paul, MN, USA). The VO_2_peak was registered after excluding obvious artefacts.

We also measured other variables (secondary outcomes) such as appetite-related sensations and energy intake in an ad libitum meal test, thermal perceptions, heart rate variability and energy expenditure in response to cold and a standardized meal^85^. However, for the shake of simplicity and clarity we decided not to include those outcomes in this manuscript.

#### Quantification and statistical analysis

A conservative approach to sample size estimation was followed, and a relatively large standard deviation assumed based on the heterogeneity of the data published on humans up to the time when the study was designed (March 2014). Increases of 10% and 20% were anticipated in activated BAT volume at 24 weeks in the MOD-EX and VIG-EX groups respectively (rising from a baseline level of 50–70 mL), along with a standard deviation of 50–60 mL. Assuming an effect in either direction, differences of at least 10% in BAT volume could be detected with a power of >80% and an α of 0.05 in a group of 17 participants per study group. To study sex differences, a total of 34 participants (17 men and 17 women) were required for each group. Assuming a maximum loss to follow-up of 30%, 150 participants were thus targeted (i.e., 50 per group). The IBM-SPSS Sample power software (version 22) was used for calculations.

Data are expressed as mean ± standard deviation (SD), unless otherwise stated. Data normality was examined using the Shapiro-Wilk test, visual histograms, and Q-Q plots. None of the blood markers were normally distributed; their values were therefore log_10_ transformed for analyses. Delta values (Δ: post-baselines values) were calculated for every outcome. To study the effect of the intervention on the primary (i.e., Δ BAT-related outcomes) and secondary (i.e., Δ body composition, Δ cardiometabolic risk factors, Δ physical fitness) endpoints, analyses of covariance (ANCOVA) were conducted including the corresponding baseline value as a covariate. Bonferroni post-hoc adjustments for multiple comparisons were used to examine differences between the three groups. As sensitivity analyses, we performed mixed-effects regression models with random intercepts to the primary hypothesis with BAT volume, BAT SUVmean, BAT SUVpeak, and BAT mean radiodensity (Supplementary Fig. S2). Pearson correlations were performed to analyze the correlation between Δ outdoor ambient temperature and Δ BAT-related outcomes calculated for specific depots. Univariate linear regression models were used to examine the association between the outdoor ambient temperature when the baseline PET/CT scan was performed and the Δ BAT-related outcomes. Partial correlations analyses were performed between the Δ body composition, the Δ cardiometabolic risk profile and the Δ physical fitness parameters, and the initial BAT-parameters and Δ BAT-related outcomes adjusting for the outdoor ambient temperature when the baseline PET/CT scan was performed. In these correlations, all *P* values were corrected by the two-stage step-up procedure of Benjamini, Krieger and Yekutieli for multiple comparisons, controlling for the false discovery rate^94^. All statistical analyses were performed using the Statistical Package for the Social Sciences v.22.0 (IBM Corporation, Chicago, IL, USA). All graphs were plotted using GraphPad Prism software v.7 (GraphPad Software, San Diego, CA, USA). Significance was set at *P* < 0.05.

### Reporting summary

Further information on research design is available in the Nature Research Reporting Summary linked to this article.

## Supplementary information

Supplementary Information

Peer Review File

Reporting Summary

## Data Availability

Source data as well as the study protocol (see supplementary note 1) are provided with this paper. All of the individual participant data collected during the trial, after deidentification, will be available for any researchers who provide a methodologically sound proposal. Proposals should be directed to ruizj@ugr.es. To gain access, data requestors will need to sign a data access agreement and the data will be provided to achieve the aims of the approved proposal. All type of analysis is allowed. These proposals may be submitted up to 60 months following article publication. After this period, data will be available in our University’s data warehouse but without investigator support other than deposited metadata. Source data are provided with this paper.

## Source data

Source Data
