# Supplementary material for: No evidence of brown adipose tissue activation after 24 weeks of supervised exercise training in young sedentary adults in the ACTIBATE randomized controlled trial
Source: Nat Commun. 2022 Sep 12;13:5259. doi: 10.1038/s41467-022-32502-x (PMC9467993; doi:10.1038/s41467-022-32502-x)
Supplement: Supplementary file 1 — Supplementary Information [file 41467_2022_32502_MOESM1_ESM.pdf]

SUPPLEMENTARY INFORMATION

The Supplementary Information includes five tables and three figures. We included as supplementary note 1 the study protocol

No evidence of brown adipose tissue activation after 24 weeks of supervised exercise training in young sedentary adults in the ACTIBATE randomized controlled trial

Borja Martinez-Tellez[1,2,3]*, Guillermo Sanchez-Delgado[1,4]*, Francisco M. Acosta[1,5,6], Juan M.A. Alcantara[1], Francisco J. Amaro-Gahete[1,7], Wendy D. Martinez-Avila[1], Elisa Merchan-Ramirez[1], Victoria Muñoz-Hernandez[1], Francisco J. Osuna-Prieto[1,8,9], Lucas Jurado-Fasoli[1], Huiwen Xu[1,10], Lourdes Ortiz-Alvarez[1,10], María J. Arias-Tellez[1,11], Andrea Mendez-Gutierrez[1,10,12,13], Idoia Labayen[14], Francisco B. Ortega[1,15], Milena Schönke[2], Patrick C.N. Rensen[2], Concepción M. Aguilera[10,12,13], José M. Llamas-Elvira[12,16,17], Ángel Gil[10,12,13], Jonatan R. Ruiz[1,12,18].

[1]PROFITH (PROmoting FITness and Health through Physical Activity) Research Group, Department of Physical Education and Sport, Faculty of Sport Sciences, University of Granada, Granada, Spain.
[2]Department of Medicine, Division of Endocrinology and Einthoven Laboratory for Experimental Vascular Medicine, Leiden University Medical Center, Leiden, The Netherlands.
[3]Department of Education, Faculty of Education Sciences and SPORT Research Group (CTS-1024), CERNEP Research Center, University of Almería, Almería, Spain
[4]Pennington Biomedical Research Center, Baton Rouge, Louisiana, USA.
[5]Turku PET Centre, University of Turku, Turku, Finland.
[6]Turku PET Centre, Turku University Hospital, Turku, Finland.
[7]Department of Medical Physiology, School of Medicine, University of Granada, Granada, Spain.
[8]Department of Analytical Chemistry, University of Granada, Granada, Spain.
[9]Research and Development of Functional Food Center (CIDAF), Granada, Spain.
[10]Department of Biochemistry and Molecular Biology II, "José Mataix Verdú" Institute of Nutrition and Food Technology (INYTA), Biomedical Research Center (CIBM), University of Granada, Granada, Spain.

[11]Department of Nutrition, Faculty of Medicine, University of Chile, Independence, 1027 Santiago, Chile.
[12]Instituto de Investigación Biosanitaria, ibs.Granada, Granada, Spain
[13]CIBER Fisiopatología de la Obesidad y la Nutrición (CIBEROBN), Madrid, Spain.
[14]Institute for Innovation & Sustainable Development in Food Chain (IS-FOOD), Public University of Navarra, Campus de Arrosadía, 31008 Pamplona, Spain.
[15]Faculty of Sport and Health Sciences. University of Jyväskylä, Jyväskylä, Finland.
[16]Nuclear Medicine Service, Virgen de las Nieves University Hospital, Granada, Spain.
[17]Nuclear Medicine Department, Biohealth Research Institute in Granada, Granada, Spain.
[18]Correspondence ruizj@ugr.es (J.R.R)
*These authors contributed equally: Borja Martinez-Tellez and Guillermo Sanchez-Delgado
Jonatan R. Ruiz, Department of Physical Education and Sports, Faculty of Sport Sciences, University of Granada, Carretera de Alfacar s/n, Granada 18071, Spain

Table S1. Effect of the 24-week supervised exercise intervention on the primary and secondary endpoints.

	CON (n=35)						MOD-EX (n=31)						VIG-EX (n=31)						
	Baseline		Post		Δ (Post-Bas)		Baseline		Post		Δ (Post-Bas)		Baseline		Post		Δ (Post-Bas)		Δ P
	Mean	SD	Mean	SD	Mean	SD	Mean	SD	Mean	SD	Mean	SD	Mean	SD	Mean	SD	Mean	SD	
Demographics																			
Age (years old)	22.0	2.0	-		-		22.0	2.1	-		-		22.0	2.5	-		-		-
Male (n, %)	15	42.8	-		-		9	29.1	-		-		10	32.2	-		-		-
Female (n, %)	20	57.2	-		-		22	70.9	-		-		21	67.8	-		-		
Brown adipose tissue outcomes																			
BAT volume (mL)	74.6	52.4	52.5	46.1	-22.2	52.6	68.9	62.9	53.4	43.3	-15.5	62.1	64.0	59.1	57.2	57.6	-6.8	66.4	0.771
BAT SUVmean	2.2	1.0	1.7	0.6	-0.4	0.7	2.1	1.2	1.7	1.1	-0.4	1.0	2.2	1.1	1.8	0.8	-0.5	1.1	1.000
BAT SUVpeak	6.7	4.1	4.1	2.5	-2.6	3.1	6.1	5.0	4.8	4.9	-1.2	4.8	6.6	5.3	4.4	3.4	-2.2	5.1	0.476
BAT mean radiodensity (HU)	-60.5	8.9	-62.0	10.2	-0.04	6.91	-60.3	9.0	-61.8	7.0	2.2	3.5	-58.0	11.0	-60.0	6.9	-2.7	10.7	0.441
Body composition																			
BMI (kg/m^2)	24.5	4.4	24.6	4.5	0.1	1.2	24.9	4.2	24.6	4.1	-0.2	0.9	25.2	4.5	24.8	4.1	-0.4	1.6	0.304
Lean mass (kg)	42.6	10.7	43.9	11.6	1.3	2.1	40.8	7.9	42.6	8.4	1.8	1.5	42.5	9.6	44.5	10.2	1.9	2.2	0.213
Fat mass (kg)	23.6	7.8	23.4	7.8	-0.3*	3.5	25.3	9.1	23.1	8.7	-2.1	2.3	26.1	8.1	23.4	7.3	-2.7*	3.8	**0.021**
Fat mass (%)	34.3	7.2	33.6	7.0	-0.8*†	3.4	36.4	8.7	33.5	8.4	-2.9*	2.5	36.4	6.9	33.1	7.2	-3.3†	3.4	**0.006**
VAT mass (g)	333	173	324	191	-9*	90	351	181	307	176	-45	58	365	190	298	157	-67*	91	**0.021**
Cardiometabolic risk factors																			
Glucose (mg/dL)	87.6	6.6	87.7	5.5	0.7	5.6	87.8	7.1	90.5	5.6	2.8	7.1	86.7	5.8	88.4	4.9	1.5	5.9	0.092
Insulin (µIU/mL)	8.2	5.4	6.1	2.5	-1.2	3.4	8.3	4.5	7.1	3.2	-1.2	3.2	8.6	4.2	7.9	4.6	-0.6	4.0	0.459
HOMA-IR	1.8	1.4	1.3	0.6	-0.3	0.8	1.8	1.2	1.6	0.8	-0.3	0.8	1.9	1.0	1.7	1.0	-0.1	0.9	0.390
γ-GT (IU/L)	22.5	28.1	19.2	11.3	-3.1	26.3	15.6	8.4	14.2	5.6	-1.4	4.8	19.1	9.7	16.7	8.2	-2.4	10.0	0.227
ALT (IU/L)	24.4	32.4	18.7	14.4	-6.0	23.2	15.5	6.1	13.6	5.4	-1.9	3.0	17.6	8.9	15.9	7.3	-1.5	4.0	0.440
ALP (IU/L)	71.9	22.8	67.2	17.9	-3.8	11.9	72.4	18.4	72.1	26.9	-0.3	13.7	74.1	16.5	76.5	16.5	2.1	11.4	0.098
Cholesterol (mg/dL)	154.8	27.8	161.2	33.6	7.1	20.7	163.8	31.1	166.8	27.0	3.0	21.0	170.4	33.2	174.0	34.8	3.4	22.1	0.994
HDL-C (mg/dL)	53.0	10.0	55.2	10.3	1.3	8.9	52.6	11.3	56.4	10.4	3.8	9.1	51.7	13.9	56.8	13.8	4.6	8.4	0.331
LDL-C (mg/dL)	86.2	22.9	91.6	25.7	6.2	14.4	95.9	28.5	93.0	20.7	-2.9	20.0	100.2	24.6	98.4	25.2	-1.7	18.4	0.488
Triacylglycerols (mg/dL)	78.5	44.0	72.7	36.5	-1.4	20.4	87.1	63.0	87.3	55.3	0.3	28.7	93.2	48.8	95.9	51.2	4.1	36.6	0.447
CRP (mg/L)	2.2	2.3	2.0	2.3	0.1	1.3	3.5	5.2	2.7	4.7	-0.8	4.1	2.2	2.6	2.2	2.5	0.0	3.5	0.257
Systolic blood pressure (mmHg)	116.9	12.6	111.9	12.8	-5.5	8.1	117.8	10.6	111.2	9.6	-6.6	9.7	116.6	11.4	109.7	12.3	-7.6	11.5	0.679
Diastolic blood pressure (mmHg)	69.5	7.8	72.3	7.5	2.4	9.8	72.2	5.9	72.6	8.0	0.3	7.0	72.0	8.0	72.6	9.8	0.1	8.9	0.952
Physical Fitness																			
Handgrip strength (kg)	32.3	7.1	33.4	8.6	1.8	2.9	30.6	7.8	33.0	8.0	1.9	2.0	31.2	7.7	33.6	8.4	1.8	2.8	0.993
RM leg press (kg)	212.5	75.0	216.8	85.3	3.7*	30.8	190.7	56.9	216.9	59.5	20.5	31.3	200.6	68.6	236.7	71.7	26.3*	37.7	**0.049**
RM bench press (kg)	34.4	16.5	34.0	17.6	0.3	5.3	28.6	10.7	31.2	14.3	0.5	5.1	30.9	13.2	32.8	11.7	0.7	6.2	0.956
Time to exhaustion (s)	883	234	940	258	21*†	124	942	192	1082	239	144*	109	953	180	1064	220	142†	96	**<0.001**
VO_2peak (mL/kg/min)	42.0	9.2	42.5	9.4	0.5*†	6.3	40.7	6.7	45.2	10.1	4.5*	6.2	41.0	7.9	45.3	7.7	4.7†	5.0	**0.011**
VO_2peak (mL/min)	2955	858	2994	949	27*†	370	2817	543	3122	672	304*	369	2971	866	3189	785	285†	375	**0.006**
Personalized cold exposure																			
Cooling vest water temperature (ºC)	5.7	1.8	6.4	2.0	0.74	1.99	5.7	1.9	5.7	1.3	-0.02	2.22	6.0	2.4	5.0	4.5	-0.99	5.23	0.154

Outdoor temperature before the PET/CT scan (ºC)	15.4	4.1	16.9	3.4	1.5	6.6	14.5	4.4	17.5	3.6	3.2	6.9	15.9	4.2	16.4	3.5	0.5	6.8	0.904
Day when baseline PET/CT scan was performed	305.9	19.8	-	-	-	-	310.2	18.5	-	-	-	-	304.6	18.5	-	-	-	-	-

Data are presented as means and standard deviations (SD) unless otherwise stated. Analyses of covariance were used to compare the Δ values (post-intervention - baseline). Serum concentrations were $\log_{10}$ transformed. * and † indicate significant differences between groups after Bonferroni correction. BAT mean radiodensity sample size was CON n=25; MOD-EX n=17; VIG-EX n=16. BAT SUVmean and SUVpeak are shown relative to lean mass. γ-GT: gamma-galactosyltransferase; ALP: alkaline phosphatase; ALT: alanine aminotransferase; BAT: brown adipose tissue; BMI: body mass index; CON: control group; CRP: C-reactive protein; EI: energy intake; HDL-C: high density lipoprotein cholesterol; HOMA-IR: Homeostatic Model Assessment for Insulin Resistance; HU: Hounsfield Units; LDL-C: low density lipoprotein cholesterol; MOD-EX: Moderate-intensity exercise group; RM: repetition maximum; PET/CT: positron emission tomography/computed tomography scan; SUV: standardized uptake value; VAT: visceral adipose tissue; VIG-EX: Vigorous-intensity exercise group; VO_2: oxygen consumption.

Table S2. Pearson correlations between outdoor ambient temperature when the baseline or post-intervention positron emission tomography/computed tomography (PET/CT) scans were performed with baseline, or post-intervention, brown adipose tissue (BAT)-related outcomes.

	BAT VOLUME		BAT SUVmean		BAT SUVpeak		BAT mean radiodensity	
**BASELINE**								
All sample (n=97)								
	r	P	r	P	r	P	r	P
Baseline outdoor ambient temperature	-0.430	**<0.001**	-0.364	**<0.001**	-0.409	**<0.001**	-0.104	0.398
**POST-INTERVENTION**								
All sample (n=97)								
	r	P	r	P	r	P	r	P
Post-intervention outdoor ambient temperature	-0.201	0.050	-0.146	0.157	-0.179	0.081	0.021	0.858
CON (N=35)								
	r	P	r	P	r	P	r	P
Post-intervention outdoor ambient temperature	-0.224	0.196	-0.073	0.678	-0.166	0.340	-0.083	0.670
MOD-EX (N=31)								
	r	P	r	P	r	P	r	P
Post-intervention outdoor ambient temperature	-0.064	0.739	-0.195	0.302	-0.241	0.200	0.246	0.247
VIG-EX (N=31)								
	r	P	r	P	r	P	r	P
Post-intervention outdoor ambient temperature	-0.287	0.118	-0.149	0.422	-0.151	0.416	-0.007	0.975

CON: control group; MOD-EX: moderate-intensity exercise group; VIG-EX: vigorous-intensity exercise group.

Table S3. Effect of the 24-week supervised exercise intervention on brown adipose tissue (BAT) volume, 18F-fluorodeoxyglucose (^{18}F-FDG) uptake (Standardized uptake Value [SUV] mean and peak), and mean radiodensity.

Outcomes	P-value
**Model 1**	
Δ BAT volume (mL)	0.782
Δ BAT SUVmean	0.961
Δ BAT SUVpeak	0.433
Δ Total BAT mean radiodensity (HU)	0.182
**Model 2**	
Δ BAT volume (mL)	0.789
Δ BAT SUVmean	0.929
Δ BAT SUVpeak	0.332
Δ Total BAT mean radiodensity (HU)	0.815

Δ was calculated as post-intervention minus baseline values for every outcome. Model 1; P values are from analyses of covariance adjusting for baseline values and baseline outdoor ambient temperature (n=97). Model 2; P values are from analyses of covariance adjusting for baseline values and Δ outdoor ambient temperature (n=97) Total BAT mean radiodensity (control group n=25; Moderate-intensity exercise group n=17; Vigorous-intensity exercise group n=16).

Table S4: Partial correlations between baseline and post-intervention brown adipose tissue (BAT)-related outcomes adjusted for baseline outdoor ambient temperature.

		**POST-INTERVENTION**							
		BAT Volume		BAT SUVmean		BAT SUVpeak		BAT mean radiodensity	
	**BASELINE**	r	P	r	P	r	P	r	P
CON (n=35)	BAT Volume	0.539	**0.007**	-	-	-	-	-	-
	BAT SUVmean	-	-	0.690	**<0.001**	-	-	-	-
	BAT SUVpeak	-	-	-	-	0.634	**0.001**	-	-
	BAT mean radiodensity	-	-	-	-	-	-	0.775	**<0.001**
MOD-EX (n=31)	BAT Volume	0.085	0.755	-	-	-	-	-	-
	BAT SUVmean	-	-	0.494	0.052	-	-	-	-
	BAT SUVpeak	-	-	-	-	0.407	0.118	-	-
	BAT mean radiodensity	-	-	-	-	-	-	0.872	**<0.001**
VIG-EX (n=31)	BAT Volume	0.516	**0.049**	-	-	-	-	-	-
	BAT SUVmean	-	-	0.344	0.210	-	-	-	-
	BAT SUVpeak	-	-	-	-	0.515	**0.050**	-	-
	BAT mean radiodensity	-	-	-	-	-	-	0.275	0.321

Similar results were obtained when Δ outdoor ambient temperature was used instead of baseline outdoor ambient temperature (data not shown). CON: control group; MOD-EX: Moderate-intensity exercise group; VIG-EX: Vigorous-intensity exercise group.

Table S5. Associations between exercise-induced changes in secondary endpoints and changes in brown adipose tissue variables.

	CON									MOD-EX									VIG-EX								
		ΔBAT volume (mL)		ΔBAT SUVmean		ΔBAT SUVpeak		ΔBAT mean radiodensity (HU)			ΔBAT volume (mL)		ΔBAT SUVmean		ΔBAT SUVpeak		ΔBAT mean radiodensity (HU)			ΔBAT volume (mL)		ΔBAT SUVmean		ΔBAT SUVpeak		ΔBAT mean radiodensity (HU)	
	N	r	P	r	P	r	P	r	P	N	r	P	r	P	r	P	r	P	N	r	P	r	P	r	P	r	P
ΔBody composition																											
BMI (kg/m^2)	34	-0.04	0.80	0.00	0.99	0.04	0.77	-0.07	0.73	30	0.01	0.95	0.07	0.72	0.05	0.80	-0.21	0.44	30	-0.14	0.45	-0.18	0.31	-0.15	0.40	-0.66	**0.01**
Lean mass (kg)	34	-0.05	0.76	-0.17	0.30	-0.06	0.70	-0.27	0.17	30	-0.15	0.43	-0.12	0.52	-0.18	0.33	0.27	0.39	30	-0.17	0.36	-0.22	0.20	-0.11	0.52	-0.29	0.26
Fat mass (kg)	34	-0.03	0.86	0.12	0.47	0.11	0.48	0.04	0.85	30	0.09	0.61	0.07	0.72	0.10	0.60	-0.30	0.26	30	-0.08	0.67	0.01	0.95	-0.04	0.83	-0.53	0.05
Fat mass (%)	34	0.02	0.89	0.13	0.44	0.10	0.53	0.06	0.75	30	0.21	0.24	0.16	0.37	0.21	0.23	-0.23	0.40	30	-0.03	0.88	0.12	0.51	0.02	0.92	-0.27	0.33
VAT mass (g)	34	-0.01	0.97	0.06	0.71	0.01	0.94	0.19	0.32	30	0.27	0.13	0.23	0.19	0.26	0.14	-0.38	0.15	30	0.22	0.22	0.20	0.25	0.12	0.50	-0.16	0.58
ΔCardiometabolic risk factors																											
Glucose (mg/dL)	32	0.10	0.53	0.03	0.85	0.13	0.42	0.22	0.27	30	0.04	0.84	0.06	0.73	-0.02	0.93	-0.36	0.17	29	-0.15	0.41	0.06	0.74	0.07	0.68	0.13	0.64
Insulin (µIU/mL)	32	0.03	0.86	-0.31	0.06	-0.16	0.32	0.24	0.23	30	-0.08	0.65	-0.02	0.93	-0.12	0.52	-0.28	0.31	29	0.16	0.42	0.04	0.84	0.09	0.62	0.37	0.22
HOMA-IR	32	0.04	0.81	-0.29	0.08	-0.13	0.42	0.26	0.20	30	-0.07	0.69	-0.01	0.98	-0.11	0.53	-0.37	0.18	29	0.12	0.54	0.05	0.81	0.09	0.61	0.38	0.20
Cholesterol (mg/dL)	32	0.38	**0.02**	0.34	0.05	0.34	**0.03**	0.03	0.89	30	0.47	**0.01**	0.32	0.07	0.36	**0.04**	0.21	0.44	29	0.20	0.29	0.26	0.15	0.12	0.51	0.26	0.38
LDL-C (mg/dL)	32	0.35	**0.03**	0.22	0.20	0.26	0.10	0.00	0.99	30	0.31	0.08	0.17	0.36	0.25	0.18	0.05	0.85	29	0.22	0.24	0.21	0.26	0.10	0.59	0.20	0.55
HDL-C (mg/dL)	32	0.37	**0.02**	0.44	**0.01**	0.39	**0.01**	0.01	0.96	30	0.46	**0.01**	0.44	**0.01**	0.41	**0.02**	0.50	0.08	29	0.24	0.19	0.42	**0.01**	0.24	0.15	0.39	0.15
Triacylglycerols (mg/dL)	32	0.12	0.49	-0.06	0.74	-0.03	0.87	0.06	0.78	30	-0.02	0.90	-0.05	0.80	-0.14	0.44	-0.13	0.64	29	-0.18	0.33	-0.18	0.30	-0.15	0.37	0.10	0.71
Systolic blood pressure (mmHg)	33	0.13	0.42	0.13	0.44	0.10	0.54	0.21	0.30	26	0.19	0.33	0.12	0.57	0.17	0.39	-0.18	0.54	29	0.08	0.66	-0.03	0.87	-0.08	0.66	-0.04	0.88
Diastolic blood pressure (mmHg)	33	0.20	0.21	0.02	0.91	-0.05	0.77	0.21	0.27	26	0.10	0.61	0.13	0.53	0.14	0.49	-0.27	0.33	29	0.09	0.64	-0.10	0.58	-0.11	0.51	-0.05	0.87
ΔPhysical fitness																											
Handgrip strength (kg)	27	-0.03	0.85	-0.06	0.75	0.14	0.41	-0.11	0.63	28	-0.05	0.81	0.18	0.37	0.18	0.37	0.17	0.53	27	0.20	0.28	0.07	0.70	0.18	0.33	0.24	0.39
RM leg press (kg)	27	-0.02	0.91	0.38	0.05	0.34	0.05	-0.19	0.39	28	-0.23	0.21	-0.15	0.42	-0.20	0.28	-0.07	0.81	27	-0.08	0.69	0.24	0.19	0.20	0.27	-0.15	0.60
RM bench press (kg)	26	-0.04	0.82	0.06	0.77	0.19	0.28	-0.14	0.57	28	-0.08	0.68	0.11	0.57	0.05	0.81	-0.26	0.34	27	-0.11	0.60	0.01	0.97	-0.04	0.81	-0.34	0.24
VO_2peak (mL/min)	32	0.11	0.50	0.00	0.99	0.09	0.59	0.08	0.67	30	0.41	**0.02**	0.11	0.54	0.22	0.22	0.18	0.51	28	-0.11	0.57	0.10	0.61	0.17	0.36	0.13	0.65
VO_2peak (mL/kg/min)	32	0.00	0.98	-0.04	0.80	0.03	0.86	-0.10	0.61	30	0.28	0.12	0.02	0.91	0.14	0.45	0.24	0.37	28	-0.08	0.70	0.14	0.44	0.23	0.21	0.51	**0.04**

Pearson r and P values obtained from partial correlation analyses adjusting for baseline outdoor ambient temperature. Significant partial correlations (P<0.05) are highlighted in bold. No correlation persisted after false discovery rate correction (all q values >0.5). Similar results were obtained when Δ outdoor ambient temperature was used instead of baseline outdoor ambient temperature (data not shown). BAT SUVmean and SUVpeak are shown relative to lean mass. BAT:

brown adipose tissue; BMI: body mass index; CON: control group; HDL-C: high density lipoprotein cholesterol; HOMA-IR: homeostatic model assessment for insulin resistance; HU: Hounsfield Units; LDL-C: low density lipoprotein cholesterol; MOD-EX: Moderate-intensity exercise group; VIG-EX: Vigorous-intensity exercise group; RM: repetition maximum; SUV: standardized uptake value; VAT: visceral adipose tissue; VO_2: oxygen consumption.

	Phases	Familiarization				Phase 1					Phase 2					Phase 3				Phase 4					
	Weeks	1	2	3	4	5	6	7	8	9	10	11	12	13	14	15	16	17	18	19	20	21	22	23	24
Endurance training	Aerobic training volume (min)	75	105	135	150	120	150	150	150	150	120	150	150	150	150	120	150	150	150	150	120	150	150	120	150
	Intensity (%HRres) MOD-EX	60	60	60	60	60	60	60	60	60	60	60	60	60	60	60	60	60	60	60	60	60	60	60	60
	Intensity (%HRres) VIG-EX	60	60	60	60	70	75	80	80	80	80	80	80	80	80	80	80	80	80	80	80	80	80	80	80
Resistance training	Intensity (%RM) MOD-EX	Weight- bearing and elastics bands					50	50	50			50	50	50			50	50	50		50	50		50	50
	Intensity (%RM) VIG-EX						50	60	70			60	70	70			60	70	70		70	70		70	70
	Number of different combined session per week	1					2					2					2				2			2	
	Type of exercises performed	Slow and global movement pattern (light weights)					Exercises localized to major muscle groups					Exercises localized to major muscle groups					Whole body exercises				Exercises localized to major muscle groups			Whole body exercises	
	Training stimulus aim	Learning of movement patterns					Initial adaptations to resistance training					Session A: mechanical tension and muscle damage					Session A: mechanical tension and muscle damage				Session A: mechanical tension and power			Session A: mechanical tension and power	
		Compensatory training: -Core stability - Flexibility - Stabilizing muscles					Compensatory training: -Core stability - Flexibility - Stabilizing muscles					Session B: Metabolic Stress					Session B: Metabolic Stress				Session B: Metabolic Stress			Session B: Metabolic Stress	

Figure S1. Supervised exercise intervention of the ACTIBATE study, adapted from Sanchez-Delgado et al. [1]. Grey blocks represent weeks in which resistance training load were reduced in order to learn the proper techniques of the exercises used in the next phase. Black blocks represent weeks in which strength training also involved repetition maximum (RM; expressed as %) assessments. HRres: heart rate reserve, expressed as %; Min: minutes; MOD-EX: Moderate-intensity exercise group; VIG-EX: Vigorous-intensity exercise group.

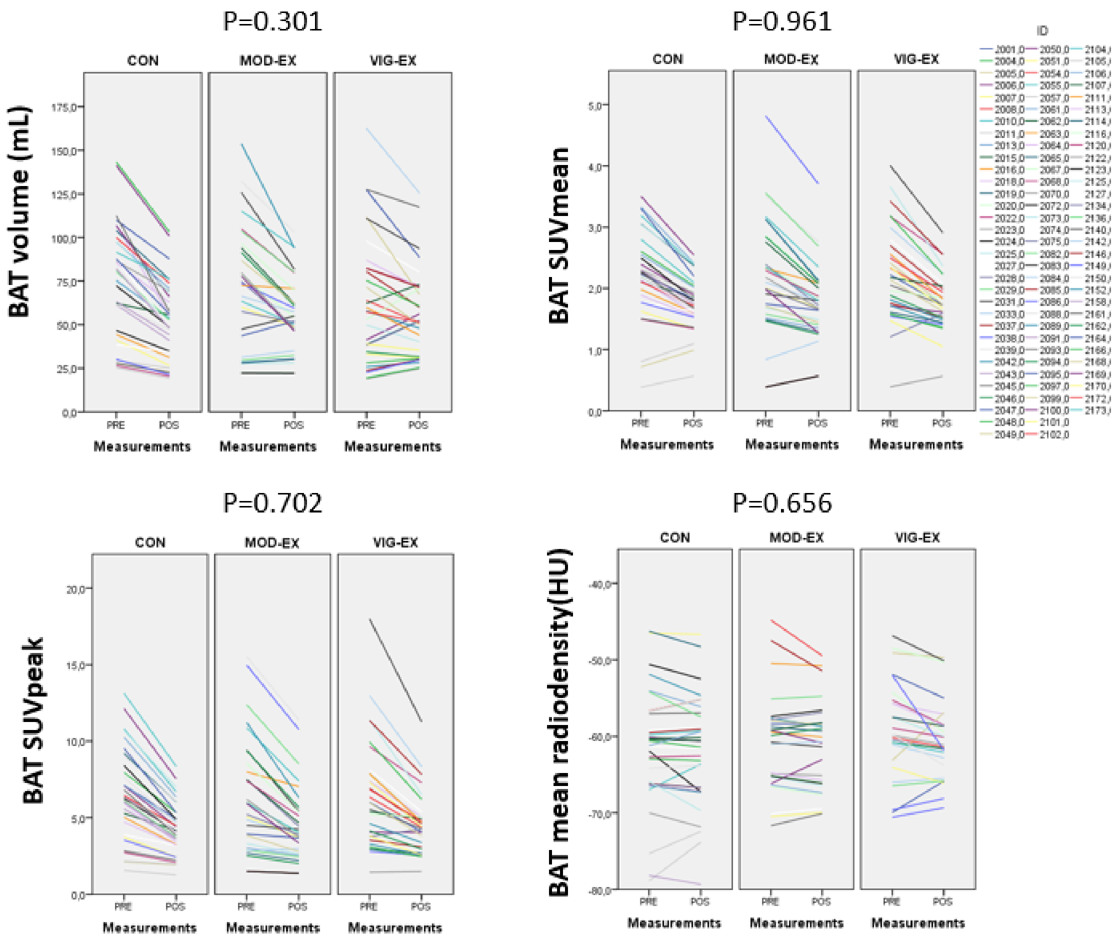

Figure S2: No evidence of brown adipose tissue activation after 24 weeks of supervised exercise training in young sedentary adults when using mixed-effects regression models. BAT: brown adipose tissue; CON: Control; HU: Hounsfield Units; ID: identification code of the participant. MOD-EX: moderate exercise intensity; PRE: before starting exercise training measurements. POS: post-intervention measurements; VIG-EX: vigorous exercise intensity.

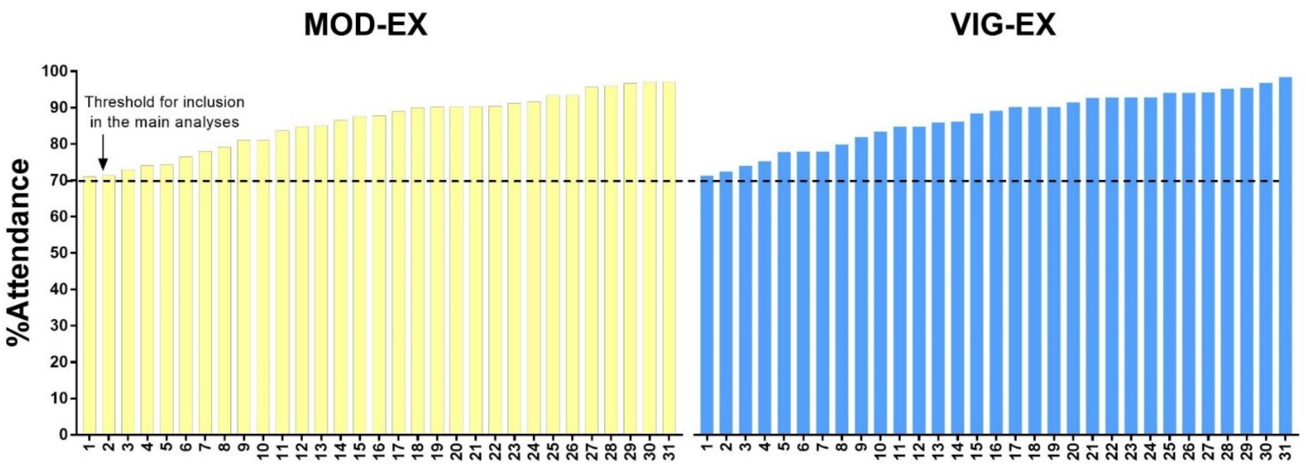

Figure S3. Waterfall plot showing individual attendance percentages for each exercise group. Each bar represents a single individual. MOD-EX: Moderate-intensity exercise group; VIG-EX: Vigorous-intensity exercise group.

Supplementary note 1

Study protocol

Original version in Spanish was APPROVED by the Ethics Committee on Human Research of the University of Granada (no. 924) and by the Servicio Andaluz de Salud (Centro de Granada, CEI-Granada, Spain). This study protocol was published in Sanchez Delgado et al. 2015. Contemporary Clinical Trials. Doi: http://dx.doi.org/10.1016/j.cct.2015.11.004

Index

LIST OF ABBREVIATIONS 14
COMPETING INTEREST 15
ACKNOWLEDGEMENTS 15
ABSTRACT 16
BACKGROUND 17
METHODS 18
Participants and selection criteria 18
Randomization and blinding 19
Sample size 19
Statistical analysis 20
Participant retention and adherence 20
Exercise program rationale and selection of doses 20
Exercise training program: 23
Training sessions 24
Outcomes measures 25
Primary outcome 25
Secondary outcomes 26
Potential impact of ACTIBATE 28
Table 1. Selection criteria. 33
Table 2. Summary of time point measurements of the primary and secondary outcomes of the study. 34
Figure 1. Flow diagram of the study participants. 35
Figure 2. Training periodization. 36
Figure 3. Examples of types of a combined training session 37

LIST OF ABBREVIATIONS

ACTIBATE: Activating Brown Adipose Tissue through Exercise
BAIBA: β-aminoisobutyric acid
BAT: Brown adipose tissue
BMI: Body mass index
BRITE: Brown-in-white
CAMD: *Centro Andaluz de Medicina del Deporte*
CIT: Cold-induced thermogenesis
DXA: Dual Energy X-ray Absorptiometry
^{18}F-FDG: ^{18}F-fluorodeoxyglucose (^{18}F-FDG)
HRres: Heart rate reserve
iMUDS: *Instituto Mixto Deporte y Salud*
MIT: Meal-induced thermogenesis
PET/CT: Positron emission tomography/computed tomography
RCT: Randomized controlled trial
REE: Resting energy expenditure
RM: Repetition maximum
SNS: Sympathetic nervous system
UCP-1: Uncoupling protein 1
VAS: Visual analogue scales

COMPETING INTEREST

The authors declare that they have no competing interests

ACKNOWLEDGEMENTS

The study is supported by the Spanish Ministry of Economy and Competitiveness, Fondo de Investigación Sanitaria del Instituto de Salud Carlos III (PI13/01393), Fondos Estructurales de la Unión Europea (FEDER), by the Spanish Ministry of Science and Innovation (RYC-2010-05957, RYC-2011-09011), by the Spanish Ministry of Education (FPU 13/04365), by the Fundación Iberoamericana de Nutrición (FINUT), by the Redes temáticas de investigación cooperativa RETIC (Red SAMID RD12/0026/0015), and by Vegenat ®.

ABSTRACT

Aims: The energy expenditure capacity of brown adipose tissue (BAT) makes it an attractive target as a therapy against obesity and type 2 diabetes. BAT activators namely catecholamines, natriuretic peptides and certain myokines, are secreted in response to exercise. ACTIBATE will determine the effect of exercise on BAT activity and mass measured by positron emission tomography/computed tomography (PET/CT, primary outcome) in young adults. ACTIBATE will also investigate the physiological consequences of activating BAT (secondary outcomes).

Methods: ACTIBATE will recruit 150 sedentary, healthy, young adults (50% women) aged 18-25 years. Eligible participants will be randomly assigned to a non-exercise group (n=50) or one of two exercise groups (n=50 each). Participants in the exercise groups will perform aerobic and strength training 3-4 days/week at a heart rate equivalent to 60% of heart rate reserve (HRres), and at 50% of 1 repetition maximum (RM) for the moderate-intensity group, and at 80% of HRres and 70%RM for the vigorous-intensity group. Laboratory measures completed at baseline and 6 months include BAT activity and mass, resting energy expenditure, meal and cold-induced thermogenesis, body temperature regulation and shivering threshold, body composition and cardiovascular disease risk factors. We will also obtain biopsies from abdominal subcutaneous white adipose tissue and skeletal muscle to analyse the expression of genes encoding proteins involved in the thermogenic machinery.

Discussion: ACTIBATE will allow quantification of the dose-response of different exercise intensities (i.e. no exercise, moderate-intensity and vigorous-intensity) BAT activity and mass in humans Findings from ACTIBATE will have significant implications for our understanding of exercise and its protective effects against the development of type 2 diabetes, obesity and related metabolic diseases.
ClinicalTrials.gov ID: NCT02365129

Key words: Brown adipose tissue; energy metabolism; thermogenesis; body temperature regulation; obesity; type 2 diabetes.

BACKGROUND

Both obesity and diabetes are serious health problems. Obesity is one of the major risk factors for cardiovascular disease, diabetes and several types of cancer. Other factors such as osteoarthritis, chronic pain, asthma, gallbladder disease and metabolic disorders are also present in obese individuals [1]. Data from the International Diabetes Federation [2] estimates that in 2035 a total of 592 million people will have diabetes. For society, both obesity and diabetes has also substantial direct and indirect costs that put a strain on healthcare and social resources [2]. Obesity is already responsible for 2-8% of health costs and 10-13% of deaths in different parts of the EU Region. In US, obesity overtook smoking as the leading contributor to the overall disease burden in 2010 [3]. Both unhealthy diets and physical inactivity are the main causes of the increasing levels of obesity and insulin resistance, which in turn lead to compensatory hyperinsulinaemia and ultimately type 2 diabetes. Although much research has been done, it is evident that our current knowledge is insufficient and new approaches have to be harnessed and exploited.

The ability to oxidize glucose and lipids and to dissipate energy as heat makes brown adipose tissue (BAT) an attractive target for anti- obesity and type 2 diabetes [4]. BAT is highly regulated by the sympathetic nervous system (SNS) to increase body temperature when mammals are exposed to temperatures below thermoneutrality. The heat production is mediated by uncoupling protein 1 (UCP-1), an inner-membrane mitochondrial protein exclusively expressed in BAT [4].

In humans, it was for long believed that BAT was only present in newborns and was responsible for non-shivering thermogenesis [5, 6]_ENREF_16. In 2009, a number of human studies showed that BAT exists and that is thermogenically active in adults [7-10]. More recently, brown adipocyte-like cells in white adipose tissue of both rodents and humans have been found. These cells, so called brown-in-white (BRITE, or beige) adipocytes, possess a multilocular morphology and express the brown adipocyte-specific UCP-1 [11, 12].

A potential clinical implication of activating BAT relates to the stimulation of resting energy expenditure (REE), meal-induced thermogenesis (MIT) [13] and cold-induced thermogenesis (CIT) [14]. In humans, MIT seems to be higher in those possessing BAT, so that they convert a higher proportion of the calories in the meal directly to heat than do individuals without brown fat [15]. CIT has been strongly correlated with BAT, and it has been estimated that BAT increases total energy expenditure on 200-400 kcal/day when humans are under cold conditions [16]. In addition, it has been proposed that even if BAT activation would not produce enough energy expenditure to induce a negative energy balance, it could exerts metabolic benefits on the prevention and treatment of type 2 diabetes and associated dyslipidaemia [17-19]_ENREF_19_ENREF_38.

The SNS, through adrenergic signalling, is the classical regulator of BAT; however, recent findings have shown a pool of novel BAT activators that sidestep the need for stimulating the SNS, such as cardiac natriuretic peptides [20], irisin [21], β-aminoisobutyric acid (BAIBA) [22], interleukin-6 (IL-6) [23], and fibroblast growth factor 21 [24]. Of interest is that both SNS and non-SNS BAT activators are sensitive to exercise, which opens new horizons to study the potential effect of exercise-based therapeutic interventions [25, 26]. Several studies conducted in animal models have reported an increased BAT activity and browning after an exercise-based intervention [21, 27-30], yet there is still some controversy [31-35]. In humans, there seem to exist a positive association of exercise with BAT and browning activity [36-38].

The primary objective of the ACTIBATE randomized controlled trial (RCT) is to quantify the dose-effect of different exercise intensities (i.e. no exercise, moderate-intensity and vigorous-intensity), on BAT activity and mass (primary outcome), and on REE, MIT, CIT, body temperature regulation and shivering threshold, body composition and cardiovascular disease risk factors in young adults. We will also obtain biopsies from abdominal subcutaneous white adipose tissue and skeletal muscle to analyse the expression of genes encoding proteins involved in the thermogenic machinery.

METHODS

The present study is a RCT (ClinicalTrials.gov ID: NCT02365129). The Human Research Ethics Committee of both University of Granada (nº 924) and *Servicio Andaluz de Salud* (*Centro de Granada, CEI-Granada*) approved the study design, study protocols and informed consent procedure. All participants will have to provide a written informed consent. Participants will be randomly allocated to the usual care (control), moderate-intensity exercise or vigorous-intensity exercise groups, and will be followed for 6 months during the exercise interventions. All the baseline and follow-up examinations will be performed in the same setting [*Instituto Mixto Deporte y Salud (iMUDS)* at the University of Granada and *Hospital Universitario Virgen de las Nieves, Granada*, Spain] and by the same investigators. The study will be performed following the ethical guidelines of the Declaration of Helsinki, last modified in 2013.

Participants and selection criteria

Participants will be students from the University of Granada (Granada, Spain). The University of Granada has ≈65,000 graduate students, of which ≈40,000 are 18-25 years. Eligible participants should be 18-25 years old and should have a body mass index (BMI) between 18.5 to 35 kg/m^2. The inclusion and exclusion criteria are listed in Table 1. We decided to implement the intervention on young adults with a wide range of BMI because overweight and obesity at this age is associated with an increased risk of cardiovascular disease among future middle-aged adults [39]. Moreover, BAT seems to be related to age and BMI [9, 40], being higher in younger and thinner people. Thus, people aged 18-25 years old are expected to present detectable BAT activity and mass [10, 41]. Further, persons of this age are legally able to sign themselves informed consent to participate in ACTIBATE, and no ethical issues are raised due to participant’s age and type of measurements to be undertaken in the study. Risks will be minimized by ruling out contraindications to the testing and training protocols via a health history and a thorough physical examination prior to the testing sessions. If any medical problems arise during the project, participants will be referred for medical evaluation and, if necessary, dropped from the study. ACTIBATE has subscribed private liability insurance covering participants, investigator's responsibility as well as the responsibility of any person involved in the study, provided that there is proper adherence to the protocol.

Information period: participants will visit the iMUDS in two occasions. In the first visit, participants will receive a thorough explanation about the study aims, measurements to be undertaken, study requirements of the participants, and intervention types. What participants can expect from the study will be clarified, and any questions will be answered. Potential interested participants will therefore receive a short web-based questionnaire to obtain information about age, weight, height, physical activity, weight during the last 3 months, current medical history and medication use, smoking and alcohol habits, and residence. Individuals who remain eligible after the first screening and express

willingness to participate in the study will be invited to a second orientation session. In the second visit, participants will receive detailed written information about the study. Eligible participants who wish to participate in further screening will be asked to sign the informed consent. Participants will then be scheduled for their first baseline measurement visit. Figure 1 illustrates the participant flow from recruitment to randomisation.

Randomization and blinding

Eligible participants will be randomly assigned after completing the baseline measurements to either the control or exercise training groups. The randomization will be computer generated. Assessment staff will be blinded to participant randomisation assignment. Participants will be explicitly informed on the group to which they will be assigned as well as on the study hypotheses and will be reminded frequently not to disclose their randomisation assignments to assessment staff in the follow-up measurements. For practical and feasibility reasons, the study will be conducted in waves of maximum 15 persons.

Sample size

The estimations of the sample size and power of our study are made based on diverse observations from acute-cold exposure studies in humans, and taking assumptions extrapolated from animal models [8, 13, 42]. We used a conservative approach to sample size estimation and assumed a relatively large standard deviation based on the heterogeneity of data published in humans. Moreover, due to the nature and duration of the study as well as the complexity of the outcomes to be measured, there were conservative estimates of effect sizes, accounting for higher drop-outs than that achieved in prior exercise-based RCTs. Power calculations are made for the primary outcome measures of BAT activity and mass.

It has been estimated that 50 g of activated BAT translates to ≈5% of REE in humans [8, 42]. An example of an ACTIBATE participant would be a sedentary man aged 19 years, body weight 90 kg, height 1.75m, and a body mass index of 29.4 kg/m^2, with a REE of ≈2000 kcal/day. We anticipated increases of 10% and 20% on activated BAT mass at 6 months in moderate-intensity and vigorous-intensity exercise groups, respectively, from a baseline level of 50 g, and change score SD of 50 g. This increase could translate into a REE of 110-120 kcal/day. Affecting energy balance by 50-100 kcal/day could prevent weight gain in the vast majority of the population [43]. Assuming a bilateral alternative (which means that an effect in either direction will be interpreted), we can detect differences of at least 10% in BAT mass with a power of >80% and α of 0.05 in a group of 17 participants. With this number of participants per group we can also detect differences of 10-20% in BAT activity with a power of >80% and α of 0.05. Since several studies reported sex-differences in BAT mass [9], all the analysis will be conducted in men and women separately. Therefore, to avoid loss of statistical power, a total of 34 (50% women) participants should be enrolled in each group. Assuming a maximum loss at follow-up of 30%, we decided to recruit 50 participants (50% women) for each of the study groups: control, moderate-intensity and vigorous-intensity groups. A total of 150 participants (of which 75 will be women and 75 men) will be enrolled in ACTIBATE. We believe this sample size is feasible and realistic based on our previous experiences in RCTs [44, 45]. We used IBM-SPSS Sample power software (version 3.0.1) for calculations.

Statistical analysis

All outcome variables will be checked for normality and results will be expressed as mean and SD or median and ranges. For between group comparisons at baseline (control group *vs* moderate-intensity and vigorous-intensity), we will analyse continuous variables with one-way analysis of variance or the non-parametric method of Kruskall-Wallis, and Chi-square tests (or exact techniques if needed), as appropriate. We will use General Lineal Models to assess the training effects (time × group interactions) on the primary and secondary study outcomes. We will adjust multiple comparisons for mass significance [46]. We will also examine the differences between drop-outs and participants who remain in the study. We will analyse the data according to the intention-to-treat principle [47], and will handle missing data due to drop-outs or non-compliance using multiple imputation methods. To fully appreciate the potential influence of missing responses, we will perform sensitivity analysis. In addition, multiple regression analysis will be performed to evaluate potential independent predictors of BAT activity.

Participant retention and adherence

Participants will be allowed to withdraw at any time; however, to reduce participants drop out and to maintain adherence to the training program, several strategies will be used (see below). In anticipation of private commitments, vacations, etc. that might interfere with a participant's availability to come to the exercise centre for an exercise session, participants will be allowed to do exercise out of the centre. We will allow participants to do a maximum of 15 unsupervised exercise sessions throughout the study. In this situation, participants will receive a heart rate monitor and instructions on how to operate it, a kit of elastic bands to perform the strength training and the session planning. They will be asked to complete a detailed log sheet to record the type of activity completed, duration of exercise, and distance travelled during exercise, if applicable.

All sessions will be accompanied with music that participants will choose, and will be performed on an airy, well-lighted exercise room. Qualified fitness specialists will carefully supervise every training session and will work with groups of no more than 10 persons to ensure that participants are performing the exercises correctly, and at proper intensity. The training specialist and other study staff will constantly support participants.

Exercise program rationale and selection of doses

With the final aim of making the exercise program transferable to society, the basis for the specific exercise dose in ACTIBATE is the physical activity recommendations for adults proposed by the World Health Organization [48]. Since there is no information regarding the ideal exercise model to activate and recruit BAT, ACTIBATE will combine both aerobic and resistance training. A major objective of the study is to evaluate various exercise intensity levels (moderate and vigorous) that fall within the current public health recommendations to test whether higher intensity levels provide more benefit than the standard moderate-intensity level.

The length of the trial is 6 months based on results from previous large scale RCT, and based on the fact that substantial physiological adaptations occur within the first 3-6 months of exercise [49]. We have also considered the increased logistical and participation burdens, which indeed might lead to poorer adherence, as well as the cost of running a highly controlled-laboratory based study for a longer period. No dietary prescription or instructions will be provided to the participants in both control and exercise groups, except for the prescription to not change their dietary habits during the study period.

Volume. An important goal in the development of exercise doses of ACTIBATE was to keep the total volume (in minutes) of weekly exercise similar in both intervention groups while ensuring that the aerobic, as well as the resistance training, prescriptions met current guidelines [48, 50]. We will achieve both goals since the total time of aerobic exercise in both moderate-intensity and vigorous-intensity groups will be 150 minutes/week, whereas the time needed to complete the resistance training exercises will be ≈80 minutes for both groups.

It is estimated that 150 minutes/week of moderate-intensity [(≈3-5.9 metabolic equivalents (METs), 1 MET= 3.5 ml O_2/kg/min] aerobic physical activity is equivalent to 1000 kcal/week, which is associated with lower rates of cardiovascular disease and premature mortality [51]. An energy expenditure of 1000 kcal/week can also be achieved with ≈75 minutes/week of vigorous intensity (≥6 METs).

We had discussions on whether the total volume of energy expended in both moderate-intensity and vigorous-intensity groups should be equal, so that it would be possible to test the independent contributions of volume expended versus intensity. The volume selected for ACTIBATE is based on the minimum weekly physical activity time recommended by the public health organizations, with the final goal of making the results of the intervention easily transferable and understandable to the population in terms of time in minutes/week, intensity, and frequency. The lack of time is one of the main reasons reported to justify not to engage in physical activity. For this reason, we decided to test two different training strategies that take the same time, but that due to the different intensities might lead to different results. Thus, both the moderate-intensity and vigorous-intensity groups will perform 150 minutes/week of aerobic exercise and ≈80 minutes/week of strength training. For the aerobic exercise, the vigorous intensity group will perform 75 minutes/week at moderate intensity (i.e. 60% of heart rate reserve (HRres)) and 75 minutes/week at vigorous intensity (i.e. 80% HRres), while the moderate-intensity group will perform the total of 150 minutes/week of aerobic training at 60% HRres. The strength training will be performed at 50% of 1 repetition maximum (RM) for the moderate-intensity group and at 70% RM for the vigorous-intensity group.

Therefore, the energy expenditure associated to the aerobic training prescribed for the vigorous-intensity group will be ≈1500 kcal/week (75 minutes/week of vigorous-intensity = 1000 kcal/week + 75 minutes/week of moderate-intensity = 500 kcal/week). We will monitor exerciser's heart rate (RS300X, Polar Electro Öy, Kempele, Finland) during the exercise sessions. We will estimate energy expenditure during the exercise sessions by calibrating energy expenditure to heart rate during the VO_{2max} tests done at baseline and at post-intervention time-points. The regression of energy expenditure on heart rate will be calculated for each participant, and energy expenditure during exercise will be estimated from the participant's average heart rate and minutes spent exercising during the training sessions. Additionally, rating of perceived effort will be collected each session [52].

Intensity. Several public health institutions recommend that moderate-intensity physical activity might be beneficial for health in deconditioned persons [48, 50, 51]. Yet, additional benefits have been observed of vigorous *vs.* moderate-intensity exercise [51]. An intensity of 60% HRres is sufficient to produce clinically significant physiological adaptations in sedentary individuals [48, 50, 51]. The intensity selected for ACTIBATE was 60% HRres for the moderate-intensity group and 80% of HRres for the vigorous-intensity group. The intensity for the resistance training will be 50% RM and 70% RM for the moderate-intensity and vigorous-intensity group, respectively. 1RM is the maximum amount of weight one can lift in a single repetition for a given exercise. An intensity equivalent to 40-50% of 1RM may be beneficial for improving muscle strength

in sedentary persons beginning a resistance training program [53], whereas 60%-70% of 1RM is recommended for novice to intermediate exercisers to improve strength [53]. As the load (i.e. %RM) is not the only variable that influences strength training intensity, we will control variables as speed of movement during both concentric and eccentric phase, recovery time and range of motion. Therefore, we assume that different loads (i.e. 50% RM and 70% RM) will really constitute different training intensities [54-56].

Frequency. Although the physical activity recommendations suggest doing physical activity on most, preferably all days of the week, there was a concern that more than 5 days per week would be and excessive burden and might have undesirable effects on adherence and motivation to the exercise intervention program. Studies on exercise frequency show little differences for 3 or more days per week provided the weekly dose of exercise is attained [51]. Participants in ACTIBATE will be asked to do their weekly exercise dose in 3 to 4 sessions, at their own choice. The length of the training sessions will be adjusted, so that participants attending 3 or 4 days/week have the same weekly dose. Strength training will be performed on 2 of these 3 or 4 days/week, and therefore 1 or 2 sessions per week will consist solely on aerobic exercise. Participants will be advised to not to be out of training for more than 2 consecutive days, and will not be allowed to train <3 days/week or >5 days/week. Participants will be contacted if they miss a scheduled session or if they do not meet the weekly recommendations.

Type of exercise. Activities programmed for the aerobic exercise are cycle ergometer, treadmill and elliptical ergometer. The resistance training program will mainly involve major upper and lower body muscle groups [53].

Training load variation. We are aware that participants might not be immediately capable of exercising at their required volume and intensity dose; therefore, there will be a gradual progression to the assigned exercise dose (see Figure 2).

Aerobic training. Participants of both exercise groups will start with a dose of 75 minutes/week at 60% HRres. The volume will increase 30 minutes/week, so by the 4th week both groups will achieve the 150 minutes/week dose. From this point onwards, the vigorous-intensity group will start a gradual increase of the exercise intensity. For this group, half of the aerobic training time will be performed at a higher intensity, which will be 5% higher than previous week (i.e. 60% HRres the 4th week, 70% HRres the 5th week, etc.), so by the 7th week, both groups will be training at the pre-specified dose.

Strength training. Participants will go through a familiarization period during the first 4 weeks of the study. During this phase, participants will learn the movement patterns that constitute the base of the different exercises (e.g. squat, horizontal pull, vertical push). They will also perform compensatory training such as core stability, flexibility and stabilizers muscles in order to minimize risk of injuries as well as to promote training adherence.

We are aware that both aerobic and strength training load should be incremented as the participants fitness increase. Aerobic training intensity is controlled based on HRres, which means that a higher speed or power has to be selected to achieve a determined percentage of HRres when fitness is increasing. For the strength training, the load equivalent to the participant's 50% RM or 70% RM will be assessed at the beginning of each training phase (i.e. each 5 weeks, see Figure 2). On the other hand, it is well known that different configurations of the strength-training stimulus can elicit different physiological responses (e.g. muscle damage, metabolic stress, etc.) [57]. Taking into account that it is not known which kind of strength training stimulus is better to activate or recruit BAT, ACTIBATE will vary the type of stimuli across the different training phases (Figure 2).

Exercise training program: Training periodization.

The training program is divided into 5 phases of different duration (Figure 2), starting with a familiarization phase.

Familiarization. This phase corresponds to the first four weeks of the training program. Participants will perform a combined (aerobic plus strength) session 3 times per week, which means that during familiarization phase there will not be "aerobic exclusive" training sessions. A sedentary person is not able to immediately train at the selected doses for ACTIBATE, therefore, the familiarization phase will allow participants to progressively increment the aerobic training volume until the selected dose is achieved (see Figure 2). On the other hand, the strength training will set light loads using elastic bands or weight bearing exercises, that will increased in load and coordinative difficulty as soon as participants are able to perform the exercises with the proper technique. Strength training will be focused on learning the main movement patterns to be used throughout the program (i.e. squat, hinge, bridge, lunge, planks, horizontal and vertical pulls, and horizontal and vertical push) and to improve: a) Core stability; b) Joint stabilizing muscles strength (e.g. rotator cuff, gluteus medius muscle, etc.); c) Balance and standing stability; and d) Flexibility. Strength training on the last week of the familiarization phase will consist on the same exercises performed in phase 1 (see below), but without considering the RM (i.e. light weights), so that participants will learn the proper movement technique.

All training phases after the familiarization period (i.e. phases 1, 2, 3 and 4) have the same structure and duration. Each phase will have 5 weeks, of which the first one will be used to evaluate the strength training load (RM indirect measure) of the exercises used in this phase. Volume of aerobic training will be reduced up to 120 minutes/week during this evaluation week. Selected intensities (50% RM and 70% RM) will be applied during the second, third and fourth week of the phase. Finally, the last week of each phase (i.e. the fifth) will be used to learn the exercises used in the next phase and its proper technique and, consequently, the load will be reduced.

In every week (except for the evaluation one), participants will perform 2 combined training sessions, and 1-2 aerobic sessions (depending on the selected frequency). Both combined sessions will be different on the structure (so called session type A or B, see Figure 3) or on the exercises performed. During the weeks of the same phase, participants will repeat the same sessions.

- ***Phase 1***. Aerobic training volume will be 150 minutes/week (except for the evaluation week, see Figure 2). The vigorous-intensity group will exercise at 70% of HRres half of the aerobic training time (i.e. 60 minutes) in the 5th week of the training program. During the following weeks, the vigorous-intensity group will gradually increase the aerobic target intensity; thus, by the 7th week of the program, this group will train at the selected training intensity.
- Strength training will consist on exercises localized in the main muscle groups (e.g. bench press, leg press, lat pull down, etc.) and will include several compensatory exercises (e.g. core stability, stabilizers muscle, etc.) similar to those performed in the familiarization phase. Combined strength sessions (which includes aerobic and strength training) will be a type A session (see figure 3) and will include a total of 4 major muscle groups, 3 core stability and 4 compensatory exercises. Participants will perform a total of 2 sets of 10 repetitions. For the vigorous-intensity group, strength training intensity will also be gradually increased until the selected dose is achieved (See Figure 2).
- ***Phase 2.*** Combined session will consist on a type A session and a type B session (see Figure 3). Strength training will include similar exercises as those reported in the previous

phase, as well as exercises involving several small muscle groups (e.g. arm curl, elbow extension, etc.), performing a total of 8-9 exercises per session. Both, session type A and session type B will include the same strength exercises. Strength exercises will be performed on 2 sets of 10 repetitions each session. On session type B, recovery time will be 1 minute between sets and between different exercises.

- ***Phase 3.*** It will include a type A session and a type B session (see Figure 3). Both session A and session B will contain the same strength exercises. Strength training will include global exercises, which use simultaneously two or more kinetic chains (e.g. Lunge+horizontal press, hinge+horizontal pull, etc.) and, consequently, constitute a vigorous stimulus to stabilizers muscles such as core muscles. Strength exercises will be performed on 2 sets of 10 repetitions each session. On session type B, recovery time will be 1 minute between sets and between different exercises.
- **Phase 4**. After the evaluation week, this phase will be divided into two periods of two weeks. It will follow an undulating periodization [58] that will use similar stimulus than that used in phase 2 and 3, but emphasizing power training (i.e. maximum concentric speed, lower eccentric phase time, intra-set recoveries, etc.). The first 2-weeks period will use the same sessions than phase 2 and the second 2-weeks period will contain the phase 3 session types (see above).

Training sessions

The duration of each individual session will depend on the number of visits per week (see above, frequency). Yet exercisers will be advised to evenly distribute the 150 minutes of aerobic physical activity throughout the week, and will be advised not to do aerobic physical activity for longer periods than 60 minutes per session. There was a concern that longer periods of aerobic exercise would be an excessive burden and might have an adverse effect on adherence to the exercise intervention program due to a higher risk of injuries, fatigue and boredom. In order to distribute homogenously the training time across sessions, aerobic training time will be distributed considering the strength training time, which means that combined training sessions (aerobic training and strength training) will have a lower aerobic volume that the aerobic ones. In every aerobic training session, participants will alternate aerobic exercise with compensatory exercises (e.g. scapular and shoulder mobility and muscles activation, core stability, hip stabilizers muscle, balance exercises, etc.) that constitute a very low training load but might help to avoid boredom during sessions and prevent injuries.
Every combined training session will start with a warm up consistent on 5 minutes of moderate intensity aerobic exercise (i.e. 60%HRres), and a set of mobility and activation exercises (e.g. front plank, monster walker, bridge, pelvic mobility, shoulder rotation, etc.). On aerobic training sessions, exercisers will only carry out the aerobic part of the warm-up. After warming-up, aerobic training will be performed on series of 10 minutes. Participants will change the ergometer (cycle ergometer, treadmill and elliptical ergometer) between series to avoid overuse of anatomical structures and boredom.
In the combined training sessions type A, strength exercises will be intercalated between aerobic series, mainly to avoid monotony that could result in participants drop out. However in type B sessions, strength training will be performed before the aerobic training, and all aerobic series will be performed one after the other. At the end of every session, participants will perform a cooling-down protocol consistent on 2 minutes of aerobic exercise at a very light intensity and a set of stretching exercises of the main muscle groups involved in the training session.

Usual care group (control). Participants randomly assigned to the usual care (control) group will receive general advices from the exercise-training specialist about the positive

effects of physical activity at the start of the study. We will prepare informative pamphlets describing the benefits of physical activity that our group has prepared for the Region of Andalucía (Spain), and that meet with the World Health Organization Guidelines. http://www.juntadeandalucia.es/salud/servicios/contenidos/andaluciaessalud/docs/130/Guia_Recomendaciones_AF.pdf.

Outcomes measures

Primary outcome measures are BAT activity and mass. Secondary outcome variables include REE, MIT, CIT, body temperature regulation and shivering threshold, body composition and cardiovascular disease risk factors. Other variables of interest include cardiorespiratory fitness and muscular strength, dietary habits, physical activity, sleep habits, health-related quality of life and other psychosocial variables, appetite, and demographic characteristics (Table 2). We will also obtain biopsies from abdominal subcutaneous white adipose tissue and skeletal muscle to analyse the expression of genes encoding proteins involved in the thermogenic machinery.

The baseline assessments are divided into 5 days:

Day 1: During the first session, each participant will go through a medical examination by the sports medicine staff from the *Centro Andaluz de Medicina del Deporte (CAMD)*. Resting electrocardiogram and blood pressure will be measured. Thereafter, participants will perform muscle strength tests (see below). Lastly, a maximum exercise test will be conducted on a treadmill (h/p cosmos, Italy).

Day 2: During the second assessment day, participants will arrive at 8:00 to the laboratory, where they will rest for 60 minutes during which time heart rate variability will be measured the first 15 minutes (Polar RS800X, Polar Electro Öy, Kempele, Finland), and REE will be measured the last 30 minutes by indirect calorimetry. Afterwards, they will have a standard breakfast (50% REE, T-Diet, Vegenat®: 16.Kcal/ml; 47% CHO, 30% FAT; 15% PRO, 3% FIBER) and energy expenditure will be measured for other 3:30 hours to determine MIT [59]. Appetite measures [visual analogue scales, (VAS)], thermal sensation and thermal comfort (VAS), and skin temperature will be monitored during and after the MIT. Lastly, body composition will be determined by Dual Energy X-ray Absorptiometry (DXA) scan (HOLOGIC, QDR 4500W).

Day 3: We will determine shivering threshold (Polar Products Inc, Ohio, USA) and CIT.

Day 4: We will conduct PET/CT scans (Siemens Biograph 16 PET/CT, Siemens, Germany), and we will collect blood sample to determine energy metabolism-related biomarkers changes in response to cold, at the *Hospital Universitario Virgen de las Nieves* (*Granada*, Spain).

Day 5: Fasting blood measurements and muscle and subcutaneous adipose tissue biopsies.

In a separate day, participants will fill in at home several web-based questionnaires regarding dietary habits, quality of life, sleeping habits, etc. Physical activity and sleep quality will be assessed through accelerometry during a week, and dietary intake will be measured by three 24h recalls.

Primary outcome: To quantify the effect of exercise intensities on BAT activity and mass, we will determine the maximal and mean standardized uptake values. We will do a PET/CT scan (Siemens Biograph 16 PET/CT, Siemens, Germany) to each participant at the cervical and supraclavicular level until mediastinum, after an individualized cooling protocol [60]. Participants will rest in a room at 18-19ºC for an hour wearing a temperature controlled water circulation cooling vest (Polar Products Inc., Ohio, USA) at a temperature 3ºC above the individually measured shivering threshold (see below). One

hour later, we will raise 1ºC the water temperature, and we will inject 74MBq (2 mCi) of ^{18}F-fluorodeoxyglucose (^{18}F-FDG) tracer and will rest for another hour with the cooling chest vest at the same room temperature. If the participant reports shivering, the water temperature will increase 1ºC until shivering stops. During all the process the skin temperature will be monitored, as well as the systolic and diastolic blood pressure. Two nuclear medicine physicians will independently interpret the images from the PET/CT. Additionally, immediately before the cold exposure (thermoneutral conditions) and after one hour of cold exposure (before the tracer injection) we will collect a blood sample to determine markers of energy metabolism such as glucose, insulin, and non-esterified fatty acids.

Secondary outcomes

To investigate the metabolic consequences of activating BAT we will measure:

- **REE**. It will be measured by indirect calorimetry (CCM express®, Medgraphics, Saint Paul, MN USA). REE will be calculated from O_2 and CO_2 volumes, as well as from urine excretion nitrogen values.
- **MIT.** It will be determined as the increase in energy expenditure above the REE divided by the energy content of a standardized meal (50% REE, T-Diet Vegenat).
- **CIT**. We will measure energy expenditure with indirect calorimetry during cold exposure of the aforementioned cooling protocol.
- All calorimetric measures will follow the accepted standard to assure the validity of the tests [61].
- **Shivering threshold**. To determine the shivering threshold, participants will wear a temperature controlled water circulation cooling vest (Polar Products Inc., Ohio, USA). The cooling vest covers the clavicular region of the participant, as well as the chest and abdominals. During the cooling protocol, participants will stay in a room at a temperature of 18-19 ºC in a semi-supine position. The cooling starts at a water vest temperature of 21ºC and water will be gradually decreased until shivering occurs [62]. We will determine shivering both visually and by asking the participants if they are experiencing shivering.
- **Body temperature regulation**. We will monitor skin temperature during (i) cooling protocols previous to the PET/TC; (ii) shivering threshold protocol; (iii) MIT test; and (iv) exercise test. Participants will wear a set of iButtons (DS 1922L, Thermochron, iButtons®; resolution: 0.0625°C, accuracy: 0.5°C; Maxim, Dallas, USA) covering the standard skin points following ISO 9886-2004 [63] defined locations to measure skin body temperature, proximal, distal and skin temperature [64]. Peripheral vasoconstriction will be estimated through the gradient of two iButtons (forearm and fingertip) [65]. Moreover, subjective cold perception will be assessed by VAS. We will also monitor supraclavicular skin temperature since it seems to have predictive value for BAT detection in adult humans [62].
- **White adipose tissue and muscle biopsies**. To analyse the expression of genes encoding proteins involved in the thermogenic machinery, mitochondriogenesis and Brite brown adipocytes, we will obtain biopsies from abdominal subcutaneous white adipose tissue at the umbilical level to quantify whether there exists browning in white adipose tissue depots, expression of UCP-1 and other potential targets involved in the browning process; and in skeletal muscle at *vastus lateralis* to quantify the expression of genes encoding proteins potentially involved in human thermogenesis. An expert surgeon will perform the biopsies following the Bergström's technique. Immunohistochemical staining and quantitative RT-PCR will be used to perform theses analyses.

- **Plasma samples and biochemical analysis.** We will also measure a full set of cardiovascular risk factors including fasting plasma lipids and lipoproteins, glucose and insulin, myeloperoxidase, e-selectin, vessel cell adhesive protein 1, and immunoglobulin cell adhesive protein, cytokines (e.g tumor necrosis factor alpha, IL-6 and monocyte chemotactic protein 1) adipokines (e.g leptin, adiponectin, resistin and active plasminogen activation inhibitor factor 1) as well as irisin and soluble (extracellular) FNDC5, orexin A and B neuropeptides, atrial natriuretic peptides, bone morphogenetic protein 7, fibroblast growth factor 21, transforming growth factor-β, and β-aminoisobutyric acid, among others. ELISA kits, western blots and High Performance Liquid Chromatography- Mass Spectroscoypy will be used to perform these analyses.
- **Cardiorespiratory fitness.** It will be determined using a maximum treadmill exercise test (h/p cosmos, Italy) following the modified Balke protocol [66], which has been extensively used and validated [67-69]. The criteria for achieving maximum oxygen consumption (VO_{2max}) will be: respiratory exchange ratio ≥1.1, a plateau in VO_2 (change of <100 ml/min in the last three consecutive 10 seconds stage), and a heart rate within 10 beats/min of the age-predicted maximal heart rate (209-0.73*age). Participants that do not achieve these criteria will be classified as having reached the VO_{2peak}. Subsequent analysis will account for this factor. The exercise electrocardiogram will be monitored continuously.
- **Muscular strength**. We will determine 1RM of the bench press and leg press, using the resistance weight machines (Keiser ®) that will be used in the exercise training sessions. We will also measure handgrip strength [70].
- **Appetite**. To examine whether changes in energy expenditure (REE, MIT, CIT) during and post-exercise intervention lead to increased appetite and therefore to an increased energy intake, we will assess (i) appetite trough questionnaires (Three-Factor Eating Questionnaire, Binge Eating Scale, Control of Eating Questionnaire), and VAS; (ii) **Dietary intake**. It will be estimated by means of a previously validated food frequency questionnaire and 3 non-consecutive 24h dietary recalls (one weekend day and two non-weekend days). Dietary and nutritional assessment will be analysed using a database for the composition of Spanish foods. We will remind participants not to change their dietary habits.
- **Body composition**. We will also measure fat mass, lean body mass, abdominal adipose tissue and bone mineral density by conducting a DXA scan (HOLOGIC, QDR 4500W). We will also measure waist circumference.
- **Physical activity**. We will monitor unstructured daily physical activity to assess potential changes in non-supervised physical activity, and to assess non-exercise activity thermogenesis. All participants will wear two activity monitors, one on the wrist and another on the hip (ActiSleep, Actigraph, Pensacola, Florida, USA) for 7 consecutive days for 24 hours to record physical activity intensity levels and patterns, as well as sleeping habits. Participants will also complete a diary log.
- **Adverse effects**. We will record adverse effects or health problems attributable to the testing sessions or intervention sessions, including muscle pain, fatigue, and general aches and pains by self-report during the study period. An independent researcher will be in charge of auditing all assessment staff to record all these events in the participants over the study period.
- **Room and outdoor temperature**. The temperature of the exercise room as well as outdoor will be recorded all through the study from the Spanish Weather Service (*Agencia Española de Meteorología, www.aemet.es/es/portada*). Participants will exercise at 20-22ºC according to the American College of Sports Medicine [71].

Potential impact of ACTIBATE

This project contributes to one of the social challenges set up by the Horizon 2020 as priority: Health, demographic change and well-being. It will improve our knowledge to combat chronic diseases as obesity and diabetes. Currently, there are no simple and non-surgical treatment strategies for treating type obesity, type 2 diabetes, and atherogenic development. While physical exercise seems to play a key role in the prevention and treatment of obesity and insulin resistance, the mechanisms that may mediate these effects are currently unknown. ACTIBATE will quantify, for the first time in humans, the effect of physical exercise on BAT activity and mass measured by PET/CT (primary outcome). Moreover, ACTIBATE will obtain biopsies from white adipose tissue and skeletal muscle tissues to analyse the expression of genes encoding proteins involved in the thermogenic machinery. We will also investigate the metabolic consequences of activating BAT by measuring REE, MIT, CIT, body temperature regulation and shivering threshold, and cardiovascular disease risk factors before and after the exercise intervention. This study integrates three different disciplines, namely exercise physiology, nutritional biochemistry, and nuclear medicine, to investigate whether exercise is able to (i) activate and recruit BAT, (ii) identify new BAT precursors and (iii) induce the specific gene program to favour white-to-brown adipocyte transformation in humans. The exercise intervention may potentially translate to a modest (around 5%) but "chronic" increase of REE. Moreover, even if BAT recruitment would not produce enough energy expenditure increase to induce a negative energy balance, it could potentially exert metabolic benefits. Findings from the ACTIBATE will have significant implications for our understanding of exercise and its protective effects against the development of type 2 diabetes, obesity and related metabolic diseases.

Supplementary note 1 references

1. Guh DP, Zhang W, Bansback N, Amarsi Z, Birmingham CL, Anis AH: **The incidence of co-morbidities related to obesity and overweight: a systematic review and meta-analysis**. *BMC Public Health* 2009, **9**:88.
2. International Diabetes Federation. IDF Diabetes Atlas teB, Belgium: International Diabetes Federation. http://www.idf.org/diabetesatlas. In.; 2013.
3. Jia H, Lubetkin EI: **Trends in quality-adjusted life-years lost contributed by smoking and obesity**. *Am J Prev Med* 2010, **38**(2):138-144.
4. Cannon B, Nedergaard J: **Brown adipose tissue: function and physiological significance**. *Physiological reviews* 2004, **84**(1):277-359.
5. Lean ME, James WP, Jennings G, Trayhurn P: **Brown adipose tissue uncoupling protein content in human infants, children and adults**. *Clinical science (London, England : 1979)* 1986, **71**(3):291-297.
6. Nedergaard J, Bengtsson T, Cannon B: **Unexpected evidence for active brown adipose tissue in adult humans**. *Am J Physiol Endocrinol Metab* 2007, **293**(2):E444-452.
7. van Marken Lichtenbelt WD, Vanhommerig JW, Smulders NM, Drossaerts JM, Kemerink GJ, Bouvy ND, Schrauwen P, Teule GJ: **Cold-activated brown adipose tissue in healthy men**. *N Engl J Med* 2009, **360**(15):1500-1508.
8. Virtanen KA, Lidell ME, Orava J, Heglind M, Westergren R, Niemi T, Taittonen M, Laine J, Savisto NJ, Enerback S *et al*: **Functional brown adipose tissue in healthy adults**. *N Engl J Med* 2009, **360**(15):1518-1525.

9. Cypess AM, Lehman S, Williams G, Tal I, Rodman D, Goldfine AB, Kuo FC, Palmer EL, Tseng YH, Doria A *et al*: **Identification and importance of brown adipose tissue in adult humans**. *N Engl J Med* 2009, **360**(15):1509-1517.
10. Saito M, Okamatsu-Ogura Y, Matsushita M, Watanabe K, Yoneshiro T, Nio-Kobayashi J, Iwanaga T, Miyagawa M, Kameya T, Nakada K *et al*: **High incidence of metabolically active brown adipose tissue in healthy adult humans: effects of cold exposure and adiposity**. *Diabetes* 2009, **58**(7):1526-1531.
11. Cao L, Choi EY, Liu X, Martin A, Wang C, Xu X, During MJ: **White to brown fat phenotypic switch induced by genetic and environmental activation of a hypothalamic-adipocyte axis**. *Cell metabolism* 2011, **14**(3):324-338.
12. Wu J, Bostrom P, Sparks LM, Ye L, Choi JH, Giang AH, Khandekar M, Virtanen KA, Nuutila P, Schaart G *et al*: **Beige adipocytes are a distinct type of thermogenic fat cell in mouse and human**. *Cell* 2012, **150**(2):366-376.
13. Rothwell NJ, Stock MJ: **A role for brown adipose tissue in diet-induced thermogenesis**. *Nature* 1979, **281**(5726):31-35.
14. Lee P, Swarbrick MM, Ho KK: **Brown adipose tissue in adult humans: a metabolic renaissance**. *Endocrine reviews* 2013, **34**(3):413-438.
15. Saito M, Yoneshiro T, Aita S: **Postprandial ther- mogenesis and brown adipose tissue in humans**. *Obesity* 2011:19(suppl 11):S80.
16. Kajimura S, Saito M: **A new era in brown adipose tissue biology: molecular control of brown fat development and energy homeostasis**. *Annual review of physiology* 2014, **76**:225-249.
17. Peirce V, Vidal-Puig A: **Regulation of glucose homoeostasis by brown adipose tissue**. *The lancet Diabetes & endocrinology* 2013, **1**(4):353-360.
18. Lee P, Smith S, Linderman J, Courville AB, Brychta RJ, Dieckmann W, Werner CD, Chen KY, Celi FS: **Temperature-acclimated brown adipose tissue modulates insulin sensitivity in humans**. *Diabetes* 2014, **63**(11):3686-3698.
19. De Lorenzo F, Mukherjee M, Kadziola Z, Sherwood R, Kakkar VV: **Central cooling effects in patients with hypercholesterolaemia**. *Clinical science (London, England : 1979)* 1998, **95**(2):213-217.
20. Bordicchia M, Liu D, Amri EZ, Ailhaud G, Dessi-Fulgheri P, Zhang C, Takahashi N, Sarzani R, Collins S: **Cardiac natriuretic peptides act via p38 MAPK to induce the brown fat thermogenic program in mouse and human adipocytes**. *J Clin Invest* 2012, **122**(3):1022-1036.
21. Bostrom P, Wu J, Jedrychowski MP, Korde A, Ye L, Lo JC, Rasbach KA, Bostrom EA, Choi JH, Long JZ *et al*: **A PGC1-alpha-dependent myokine that drives brown-fat-like development of white fat and thermogenesis**. *Nature* 2012, **481**(7382):463-468.
22. Roberts LD, Bostrom P, O'Sullivan JF, Schinzel RT, Lewis GD, Dejam A, Lee YK, Palma MJ, Calhoun S, Georgiadi A *et al*: **beta-Aminoisobutyric acid induces browning of white fat and hepatic beta-oxidation and is inversely correlated with cardiometabolic risk factors**. *Cell metabolism* 2014, **19**(1):96-108.
23. Reihmane D, Dela F: **Interleukin-6: possible biological roles during exercise**. *European journal of sport science* 2014, **14**(3):242-250.
24. Fisher FM, Kleiner S, Douris N, Fox EC, Mepani RJ, Verdeguer F, Wu J, Kharitonenkov A, Flier JS, Maratos-Flier E *et al*: **FGF21 regulates PGC-1alpha and browning of white adipose tissues in adaptive thermogenesis**. *Genes & development* 2012, **26**(3):271-281.

25. Ruiz JR, Martinez-Tellez B, Sanchez-Delgado G, Aguilera CM, Gil A: **Regulation of energy balance by brown adipose tissue: at least three potential roles for physical activity**. *Br J Sports Med* 2015.
26. Sanchez-Delgado G, Martinez-Tellez B, Olza J, Aguilera CM, Gil A, Ruiz JR: **Role of exercise in the activation of brown adipose tissue** *Ann Nutr Metab* In press.
27. De Matteis R, Lucertini F, Guescini M, Polidori E, Zeppa S, Stocchi V, Cinti S, Cuppini R: **Exercise as a new physiological stimulus for brown adipose tissue activity**. *Nutr Metab Cardiovasc Dis* 2013, **23**(6):582-590.
28. Hatano D, Ogasawara J, Endoh S, Sakurai T, Nomura S, Kizaki T, Ohno H, Komabayashi T, Izawa T: **Effect of exercise training on the density of endothelial cells in the white adipose tissue of rats**. *Scand J Med Sci Sports* 2011, **21**(6):e115-121.
29. Slocum N, Durrant JR, Bailey D, Yoon L, Jordan H, Barton J, Brown RH, Clifton L, Milliken T, Harrington W *et al*: **Responses of brown adipose tissue to diet-induced obesity, exercise, dietary restriction and ephedrine treatment**. *Experimental and toxicologic pathology : official journal of the Gesellschaft fur Toxikologische Pathologie* 2013, **65**(5):549-557.
30. Xu X, Ying Z, Cai M, Xu Z, Li Y, Jiang SY, Tzan K, Wang A, Parthasarathy S, He G *et al*: **Exercise ameliorates high-fat diet-induced metabolic and vascular dysfunction, and increases adipocyte progenitor cell population in brown adipose tissue**. *Am J Physiol Regul Integr Comp Physiol* 2011, **300**(5):R1115-1125.
31. Segawa M, Oh-Ishi S, Kizaki T, Ookawara T, Sakurai T, Izawa T, Nagasawa J, Kawada T, Fushiki T, Ohno H: **Effect of running training on brown adipose tissue activity in rats: a reevaluation**. *Research communications in molecular pathology and pharmacology* 1998, **100**(1):77-82.
32. Scarpace PJ, Yenice S, Tumer N: **Influence of exercise training and age on uncoupling protein mRNA expression in brown adipose tissue**. *Pharmacology, biochemistry, and behavior* 1994, **49**(4):1057-1059.
33. Shibata H, Nagasaka T: **The effect of forced running on heat production in brown adipose tissue in rats**. *Physiology & behavior* 1987, **39**(3):377-380.
34. Wickler SJ, Stern JS, Glick Z, Horwitz BA: **Thermogenic capacity and brown fat in rats exercise-trained by running**. *Metabolism* 1987, **36**(1):76-81.
35. Yamashita H, Yamamoto M, Sato Y, Izawa T, Komabayashi T, Saito D, Ohno H: **Effect of running training on uncoupling protein mRNA expression in rat brown adipose tissue**. *International journal of biometeorology* 1993, **37**(1):61-64.
36. Ruiz JR, Sanchez-Delgado G, Martinez-Tellez B, Aguilera CM, Gil A: **RE: Association between habitual physical activity and brown adipose tissue activity in individuals undergoing PET-CT scan**. *Clinical endocrinology* 2014.
37. Dinas PC, Nikaki A, Jamurtas AZ, Prassopoulos V, Efthymiadou R, Koutedakis Y, Georgoulias P, Flouris AD: **Association between habitual physical activity and brown adipose tissue activity in individuals undergoing PET-CT scan**. *Clinical endocrinology* 2014.
38. Lee P, Linderman JD, Smith S, Brychta RJ, Wang J, Idelson C, Perron RM, Werner CD, Phan GQ, Kammula US *et al*: **Irisin and FGF21 are cold-induced endocrine activators of brown fat function in humans**. *Cell metabolism* 2014, **19**(2):302-309.

39. Bibbins-Domingo K, Coxson P, Pletcher MJ, Lightwood J, Goldman L: **Adolescent overweight and future adult coronary heart disease**. *N Engl J Med* 2007, **357**(23):2371-2379.
40. Sturkenboom MG, Franssen EJ, Berkhof J, Hoekstra OS: **Physiological uptake of [18F]fluorodeoxyglucose in the neck and upper chest region: are there predictive characteristics?** *Nuclear medicine communications* 2004, **25**(11):1109-1111.
41. Zingaretti MC, Crosta F, Vitali A, Guerrieri M, Frontini A, Cannon B, Nedergaard J, Cinti S: **The presence of UCP1 demonstrates that metabolically active adipose tissue in the neck of adult humans truly represents brown adipose tissue**. *Faseb J* 2009, **23**(9):3113-3120.
42. van Marken Lichtenbelt WD, Schrauwen P: **Implications of nonshivering thermogenesis for energy balance regulation in humans**. *Am J Physiol Regul Integr Comp Physiol* 2011, **301**(2):R285-296.
43. Hill JO, Wyatt HR, Reed GW, Peters JC: **Obesity and the environment: where do we go from here?** *Science* 2003, **299**(5608):853-855.
44. Ruiz JR, Perales M, Pelaez M, Lopez C, Lucia A, Barakat R: **Supervised exercise-based intervention to prevent excessive gestational weight gain: a randomized controlled trial**. *Mayo Clinic proceedings* 2013, **88**(12):1388-1397.
45. Barakat R, Ruiz JR, Rodriguez-Romo G, Montejo-Rodriguez R, Lucia A: **Does exercise training during pregnancy influence fetal cardiovascular responses to an exercise stimulus? Insights from a randomised, controlled trial**. *British journal of sports medicine* 2010, **44**(10):762-764.
46. Holm S: **A simple sequentially rejective multiple test procedure**. *Scand J Statist* 1979, **6**:65-70.
47. Hollis S, Campbell F: **What is meant by intention to treat analysis? Survey of published randomised controlled trials**. *BMJ* 1999, **319**(7211):670-674.
48. WHO: **Global Recommendations on Physical Activity for Health. World Health Organ publications. Geneva, Switzerland**. 2010.
49. Pedersen BK, Saltin B: **Evidence for prescribing exercise as therapy in chronic disease**. *Scand J Med Sci Sports* 2006, **16 Suppl 1**:3-63.
50. http://www.health.gov/PAGuidelines/
51. Garber CE, Blissmer B, Deschenes MR, Franklin BA, Lamonte MJ, Lee IM, Nieman DC, Swain DP: **American College of Sports Medicine position stand. Quantity and quality of exercise for developing and maintaining cardiorespiratory, musculoskeletal, and neuromotor fitness in apparently healthy adults: guidance for prescribing exercise**. *Med Sci Sports Exerc* 2011, **43**(7):1334-1359.
52. Borg GA: **Psychophysical bases of physical exertion**. *Med Sci Sports Exerc* 1982, **14**(5):377-381.
53. ACSM: **American College of Sports Medicine position stand. Progression models in resistance training for healthy adults**. *Med Sci Sports Exerc* 2009, **41**(3):687-708.
54. **American College of Sports Medicine position stand. Progression models in resistance training for healthy adults**. *Med Sci Sports Exerc* 2009, **41**(3):687-708.
55. Gonzalez-Badillo JJ, Marques MC, Sanchez-Medina L: **The importance of movement velocity as a measure to control resistance training intensity**. *Journal of human kinetics* 2011, **29A**:15-19.

56. Roig M, O'Brien K, Kirk G, Murray R, McKinnon P, Shadgan B, Reid WD: **The effects of eccentric versus concentric resistance training on muscle strength and mass in healthy adults: a systematic review with meta-analysis**. *Br J Sports Med* 2009, **43**(8):556-568.
57. Schoenfeld BJ: **The mechanisms of muscle hypertrophy and their application to resistance training**. *J Strength Cond Res* 2010, **24**(10):2857-2872.
58. Fleck SJ: **Non-linear periodization for general fitness & athletes**. *Journal of human kinetics* 2011, **29A**:41-45.
59. Ruddick-Collins LC, King NA, Byrne NM, Wood RE: **Methodological considerations for meal-induced thermogenesis: measurement duration and reproducibility**. *Br J Nutr* 2013, **110**(11):1978-1986.
60. van der Lans AA, Wierts R, Vosselman MJ, Schrauwen P, Brans B, van Marken Lichtenbelt WD: **Cold-activated brown adipose tissue in human adults: methodological issues**. *American journal of physiology Regulatory, integrative and comparative physiology* 2014, **307**(2):R103-113.
61. Compher C, Frankenfield D, Keim N, Roth-Yousey L: **Best practice methods to apply to measurement of resting metabolic rate in adults: a systematic review**. *Journal of the American Dietetic Association* 2006, **106**(6):881-903.
62. Boon MR, Bakker LE, van der Linden RA, Pereira Arias-Bouda L, Smit F, Verberne HJ, van Marken Lichtenbelt WD, Jazet IM, Rensen PC: **Supraclavicular skin temperature as a measure of 18F-FDG uptake by BAT in human subjects**. *PLoS One* 2014, **9**(6):e98822.
63. ISO: **ISO-standard 9886:2004 (Ergonomics – Evaluation of thermal strain by physiological measurements, International Standards Organization, Geneva, Switzerland**.
64. van Marken Lichtenbelt WD, Daanen HA, Wouters L, Fronczek R, Raymann RJ, Severens NM, Van Someren EJ: **Evaluation of wireless determination of skin temperature using iButtons**. *Physiology & behavior* 2006, **88**(4-5):489-497.
65. Schellen L, Loomans MG, de Wit MH, Olesen BW, van Marken Lichtenbelt WD: **The influence of local effects on thermal sensation under non-uniform environmental conditions--gender differences in thermophysiology, thermal comfort and productivity during convective and radiant cooling**. *Physiology & behavior* 2012, **107**(2):252-261.
66. Balke B, Ware RW: **An experimental study of physical fitness of Air Force personnel**. *U S Armed Forces Med J* 1959, **10**(6):675-688.
67. Sui X, LaMonte MJ, Laditka JN, Hardin JW, Chase N, Hooker SP, Blair SN: **Cardiorespiratory fitness and adiposity as mortality predictors in older adults**. *JAMA* 2007, **298**(21):2507-2516.
68. Ortega FB, Lee DC, Katzmarzyk PT, Ruiz JR, Sui X, Church TS, Blair SN: **The intriguing metabolically healthy but obese phenotype: cardiovascular prognosis and role of fitness**. *Eur Heart J* 2013, **34**(5):389-397.
69. Wei M, Kampert JB, Barlow CE, Nichaman MZ, Gibbons LW, Paffenbarger RS, Jr., Blair SN: **Relationship between low cardiorespiratory fitness and mortality in normal-weight, overweight, and obese men**. *JAMA* 1999, **282**(16):1547-1553.
70. Ruiz-Ruiz J, Mesa JL, Gutierrez A, Castillo MJ: **Hand size influences optimal grip span in women but not in men**. *J Hand Surg [Am]* 2002, **27**(5):897-901.
71. Tharrett s, Mcinnis K, Peterson J: **ACSM's Health / Fitness Facility Standards and Guidelines, 3[a] ed. Champaign, Illinois: Human Kinetics.** 2007.

Table 1. Selection criteria.

Inclusion criteria	Exclusion criteria
-Age: 18-25 years.<br>-BMI: 18.5-35 kg/m^2.<br>-Not engaged in regular physical activity >20 min on >3 days/week.<br>-Not participating in a weight loss program.<br>-Stable weight over the last 3 months (body weight changes <3 kg).<br>-Normal electrocardiogram.<br>-Participants must be capable and willing to provide consent, understand exclusion criteria and accept the randomized group assignment.	- History of cardiovascular disease.<br>- Diabetes or hypertension.<br>- Pregnancy, or planning to get pregnant during the study period.<br>-Medication for hypertension, hyperlipidemia, hyperuricemia or other illness.<br>-Beta blockers or benzodiapezins use.<br>-Smoking.<br>-Frequent exposure to cold temperatures (Granada is surrounded by high mountains where people can ski or do trekking).<br>-Taking medication for thyroid.<br>-Other significant medical conditions that are life-threatening or that can interfere with or be aggravated by exercise.<br>- Unwillingness to either complete the study requirements or to be randomized into control or training group.<br>- A first-degree relative with history of cancer.

Table 2. Summary of time point measurements of the primary and secondary outcomes of the study.

Measurements	1-<br>Baseline<br>(week 0)	2-<br>Week<br>4	3-<br>Week<br>8	4-<br>Week<br>12	5-<br>Week<br>16	6-<br>Week<br>20	7-<br>Post-int<br>(week 25)
Primary outcome							
BAT activity and mass	x						x
Secondary outcomes							
Resting energy expenditure	x		x		x		x
Meal induced thermogenesis	x						x
Cold-induced thermogenesis	x						x
Shivering threshold	x						x
Body temperature regulation	x						x
Body composition	x		x		x		x
CVD risk factors	x						x
Metabolomics	x						x
Proteomics	x						x
Morphology of tissues	x						x
Dietary assessment	x		x		x		x
Appetite questionnaires	x		x		x		x
Physical activity (monitors)	x		x		x		x
Sleeping habits	x		x		x		x
Cardiorespiratory fitness	x		x		x		x
Muscular strength	x		x		x		x
Health-related quality of life	x						x
Side effects	x	x	x	x	x	x	x

FIGURE LEGENDS

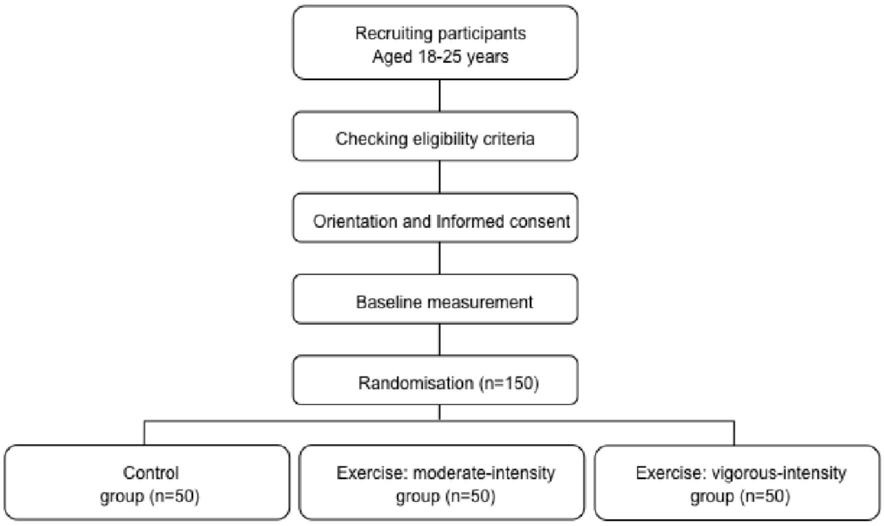

Figure 1. Flow diagram of the study participants.

	Phases	Familiarization				Phase 1					Phase 2					Phase 3				Phase 4					
	Weeks	1	2	3	4	5	6	7	8	9	10	11	12	13	14	15	16	17	18	19	20	21	22	23	24
Endurance training	Aerobic training volume (min)	75	105	135	150	120	150	150	150	150	120	150	150	150	150	120	150	150	150	150	120	150	150	120	150
	Intensity (%HRres) MOD-EX	60	60	60	60	60	60	60	60	60	60	60	60	60	60	60	60	60	60	60	60	60	60	60	60
	Intensity (%HRres) VIG-EX	60	60	60	60	70	75	80	80	80	80	80	80	80	80	80	80	80	80	80	80	80	80	80	80
Resistance training	Intensity (%RM) MOD-EX	Weight- bearing and elastics bands					50	50	50			50	50	50			50	50	50		50	50		50	50
	Intensity (%RM) VIG-EX						50	60	70			60	70	70			60	70	70		70	70		70	70
	Number of different combined session per week	1					2					2					2				2			2	
	Type of exercises performed	Slow and global movement pattern (light weights)					Exercises localized to major muscle groups					Exercises localized to major muscle groups					Whole body exercises				Exercises localized to major muscle groups			Whole body exercises	
	Training stimulus aim	Learning of movement patterns					Initial adaptations to resistance training					Session A: mechanical tension and muscle damage					Session A: mechanical tension and muscle damage				Session A: mechanical tension and power			Session A: mechanical tension and power	
		Compensatory training: -Core stability - Flexibility - Stabilizing muscles					Compensatory training: -Core stability - Flexibility - Stabilizing muscles					Session B: Metabolic Stress					Session B: Metabolic Stress				Session B: Metabolic Stress			Session B: Metabolic Stress	

Figure 2. Training periodization.

Grey blocks represent weeks where the strength training load is reduced in order to learn the proper technique of the exercise used in the next phase. Black blocks represent weeks where strength training mostly consists on repetition maximum (RM) assessment in the different exercises used in the phase. HR: Heart rate reserve.

SESSION TYPE A		
Warm-up		
MAIN PART	**Exercise**	**Time/sets**
	Aerobic set 1	10 min
	Strength exercise A	1set x 10reps
	Strength exercise B	1set x 10reps
	Strength exercise C	1set x 10reps
	Strength exercise D	1set x 10reps
	Aerobic set 2	10 min
	Strength exercise E	1set x 10reps
	Strength exercise F	1set x 10reps
	Strength exercise G	1set x 10reps
	Strength exercise H	1set x 10reps
	Aerobic set 3	10 min
	Strength exercise A	1set x 10reps
	Strength exercise B	1set x 10reps
	Strength exercise C	1set x 10reps
	Strength exercise D	1set x 10reps
	Aerobic set 4	10 min
	Strength exercise E	1set x 10reps
	Strength exercise F	1set x 10reps
	Strength exercise G	1set x 10reps
	Strength exercise H	1set x 10reps
Cooling down		

SESSION TYPE B		
Warm-up		
MAIN PART	**Exercise**	**Time/sets**
	Strength exercise A	2sets x 10reps
	Strength exercise G	2sets x 10reps
	Strength exercise E	2sets x 10reps
	Strength exercise C	2sets x 10reps
	Strength exercise B	2sets x 10reps
	Strength exercise F	2sets x 10reps
	Strength exercise D	2sets x 10reps
	Strength exercise H	2sets x 10reps
	Aerobic set 1	10 min
	Aerobic set 2	10 min
	Aerobic set 3	10 min
	Aerobic set 4	10 min
Cooling down		

Figure 3. Examples of types of a combined training session.

In session type A strength straining aims to maximize mechanical tensions and muscle damage, while in session type B strength training aims to maximize metabolic stress. On session type A, the exercise order alternate between upper-body and lower-body, and agonist and antagonist exercises, allowing the maximum recovery possible to the implied muscle groups. On session type B, all upper-body exercises are performed before the lower-body exercises, and all agonist muscle groups exercises are also performed afterward each other.
