# Supplementary material for: No evidence of brown adipose tissue activation after 24 weeks of supervised exercise training in young sedentary adults in the ACTIBATE randomized controlled trial
Source: Nat Commun. 2022 Sep 12;13:5259. doi: 10.1038/s41467-022-32502-x (PMC9467993; doi:10.1038/s41467-022-32502-x)
Supplement: Supplementary file 3 — Reporting Summary [file 41467_2022_32502_MOESM3_ESM.pdf]

nature portfolio

Corresponding author(s): Jonatan Ruiz Ruiz

Last updated by author(s): Aug 2, 2022

Reporting Summary

Nature Portfolio wishes to improve the reproducibility of the work that we publish. This form provides structure for consistency and transparency in reporting. For further information on Nature Portfolio policies, see our Editorial Policies and the Editorial Policy Checklist.

Statistics

For all statistical analyses, confirm that the following items are present in the figure legend, table legend, main text, or Methods section.

n/a	Confirmed	
☐	☒	The exact sample size (*n*) for each experimental group/condition, given as a discrete number and unit of measurement
☐	☒	A statement on whether measurements were taken from distinct samples or whether the same sample was measured repeatedly
☐	☒	The statistical test(s) used AND whether they are one- or two-sided<br>*Only common tests should be described solely by name; describe more complex techniques in the Methods section.*
☐	☒	A description of all covariates tested
☐	☒	A description of any assumptions or corrections, such as tests of normality and adjustment for multiple comparisons
☐	☒	A full description of the statistical parameters including central tendency (e.g. means) or other basic estimates (e.g. regression coefficient) AND variation (e.g. standard deviation) or associated estimates of uncertainty (e.g. confidence intervals)
☐	☒	For null hypothesis testing, the test statistic (e.g. *F*, *t*, *r*) with confidence intervals, effect sizes, degrees of freedom and *P* value noted<br>*Give P values as exact values whenever suitable.*
☒	☐	For Bayesian analysis, information on the choice of priors and Markov chain Monte Carlo settings
☒	☐	For hierarchical and complex designs, identification of the appropriate level for tests and full reporting of outcomes
☐	☒	Estimates of effect sizes (e.g. Cohen's *d*, Pearson's *r*), indicating how they were calculated

Our web collection on statistics for biologists contains articles on many of the points above.

Software and code

Policy information about availability of computer code

Data collection

REAGENT or RESOURCE SOURCE IDENTIFIER
Biological Samples

Human plasma and serum samples Clinical Trial NCT-02365129

Chemical, Peptides, and Recombinant Proteins

Glucose assay kit Beckman Coulter #OSR6521
Insulin assay kit Beckman Coulter #OSR33410
γ-GT assay kit Beckman Coulter #OSR6507
ALT assay kit Beckman Coulter #OSR6507
ALP assay kit Beckman Coulter #OSR6204
Cholesterol assay kit Beckman Coulter #OSR6516
HDL-C assay kit Beckman Coulter #OSR6587
Triacylglycerols assay kit Beckman Coulter #OSR61118
CRP assay kit Beckman Coulter #OSR6299

Other

18F-FDG-Radiotracer IBA molecular N/A
Heart Rate Monitors Polar RS800CX
Dual x-ray absorptiometry (DXA) Hologic Discovery https://medpick.in/product/hologic-discovery-wi-bone-densitometers/
Cooling vests and chillers Polar Products Inc. https://www.polarproducts.com/polarshop/pc/Cooling-Systems-for-Operating-Rooms-and-Medical-Facilities-c444.htm

	16 PET/CT scanner Siemens Biograph https://www.siemens-healthineers.com/nl/molecular-imaging/pet-ct/biograph-vision<br>Blood pressure monitor Omrom M2 Omron Healthcare, Kyoto, Japan<br>Handgrip dynamometer (digital Grip-D) Takei https://www.newitts.com/takei-5401-hand-grip-digital-dynamometer<br>Leg press pneumatic machine (A300) Keiser Corporation https://www.keiser.com/fitness-equipment/strength-training/air300-leg-press?ssevariant=1<br>Power pneumatic rack (Model 3111) Keiser Corporation https://www.keiser.com/fitness-equipment/racks/power-rack<br>Treadmill (Pulsar treadmill) HPcosmos https://www.hpcosmos.com/en/pulsar-3p<br>CPX Ultima CardioO2 metabolic cart Medical Graphics Corp. https://mgcdiagnostics.com/products/ultima-cpx-metabolic-stress-testing-system
Data analysis	FIJI- Image J FIJI software v.1 https://sourceforge.net/p/bifijiplugins/wiki/Brown%20fat%20Volume/<br>Statistical Package for the Social Sciences v.22.0 IBM Corporation https://www.ibm.com/analytics/spss-statistics-software. RRID:SCR_002865<br>Prism software v.7 GraphPad http://www.graphpad.com/ RRID: SCR_002798

For manuscripts utilizing custom algorithms or software that are central to the research but not yet described in published literature, software must be made available to editors and reviewers. We strongly encourage code deposition in a community repository (e.g. GitHub). See the Nature Portfolio guidelines for submitting code & software for further information.

Data

Policy information about availability of data

All manuscripts must include a data availability statement. This statement should provide the following information, where applicable:

- Accession codes, unique identifiers, or web links for publicly available datasets
- A description of any restrictions on data availability
- For clinical datasets or third party data, please ensure that the statement adheres to our policy

Source data as well as the study protocol (see supplementary note 1) are provided with this paper. All of the individual participant data collected during the trial, after deidentification, will be available for any researchers who provide a methodological sound proposal. Proposals should be directed to ruizj@ugr.es. To gain access, data requestors will need to sign a data access agreement and the data will be provided to achieve the aims of the approved proposal. All type of analysis is allowed. These proposals may be submitted up to 60 months following article publication. After this period, data will be available in our University's data warehouse but without investigator support other than deposited metadata.

Field-specific reporting

Please select the one below that is the best fit for your research. If you are not sure, read the appropriate sections before making your selection.

☒ Life sciences ☐ Behavioural & social sciences ☐ Ecological, evolutionary & environmental sciences

For a reference copy of the document with all sections, see nature.com/documents/nr-reporting-summary-flat.pdf

Life sciences study design

All studies must disclose on these points even when the disclosure is negative.

Sample size	A conservative approach to sample size estimation was followed, and a relatively large standard deviation assumed based on the heterogeneity of the data published on humans up to the time when the study was designed (March 2014). Increases of 10% and 20% were anticipated in activated BAT volume at 24 weeks in the MOD-EX and VIG-EX groups respectively (rising from a baseline level of 50-70 mL), along with a standard deviation of 50-60 mL. Assuming an effect in either direction, differences of at least 10% in BAT volume could be detected with a power of >80% and an α of 0.05 in a group of 17 subjects per study group. To study sex differences, a total of 34 subjects (17 men and 17 women) were required for each group. Assuming a maximum loss to follow-up of 30%, 150 subjects were thus targeted (i.e., 50 per group). The IBM-SPSS Sample power software (version 3.0.1) was used for calculations.
Data exclusions	8 participants were exlcuded from the exercise ECG, because they showed abnormal ECG.<br>2 participants were exlcuded because they did not meet the inclusion criteria after medical screening<br>39 participants declided to participate before starting the trial without reason.<br>During the study intervention, 38 participants stopped their participation in the study without reporting the reason.
Replication	Not applicable. The measurements methods (such as PET/CT scans, blood sample and biopisies collection) are not suitable for replication to its invasive nature and susceptability of including carry-over effects.
Randomization	After baseline examination, subjects were assigned to one of three groups via computer-generated simple unrestricted randomization by JRR: (i) a control group (no exercise, CON), (ii) a moderate-intensity exercise group (MOD-EX), and (iii) a vigorous-intensity exercise group (VIG-EX).
Blinding	The PI was blinded during the whole protocol, but for the researchers that were performing the study was not possible. SInce the same researchers who were doing the exercise intervention, were the same researchers who measured the primary and secondary outcomes.

Reporting for specific materials, systems and methods

We require information from authors about some types of materials, experimental systems and methods used in many studies. Here, indicate whether each material, system or method listed is relevant to your study. If you are not sure if a list item applies to your research, read the appropriate section before selecting a response.

Materials & experimental systems

n/a	Involved in the study
☒	☐ Antibodies
☒	☐ Eukaryotic cell lines
☒	☐ Palaeontology and archaeology
☒	☐ Animals and other organisms
☐	☒ Human research participants
☐	☒ Clinical data
☒	☐ Dual use research of concern

Methods

n/a	Involved in the study
☒	☐ ChIP-seq
☒	☐ Flow cytometry
☒	☐ MRI-based neuroimaging

Human research participants

Policy information about studies involving human research participants

Population characteristics	97 participants (65% women) finished the current randomized controlled trial. All of them were relatively healthy (Table 1)
Recruitment	Participants were recruited through newspaper advertisements in the vicinity of Granada, Spain. Selection bias could have been occured, which could result in including only the men and women who are interested and motivated to comply to an exercise intervention for 24 weeks. This could affect feasability of the intervention and can not be directly translated to the general population. However this study was designed to test the effect of exercise as potential activator of BAT, and not the feasability or implantation in daily life directly. So the recruitment strategy is not expected to affect the outcomes of this study.
Ethics oversight	The study was approved by the Ethics Committee on Human Research of the University of Granada (no. 924) and by the Servicio Andaluz de Salud (Centro de Granada, CEI-Granada, Spain). All subjects provided informed consent to be included.

Note that full information on the approval of the study protocol must also be provided in the manuscript.

Clinical data

Policy information about clinical studies

All manuscripts should comply with the ICMJE guidelines for publication of clinical research and a completed CONSORT checklist must be included with all submissions.

Clinical trial registration	The study was registered at ClinicalTrials.gov ID: NCT02365129
Study protocol	The original full protocol is available as supplementary note 1 in the supplementary material
Data collection	The first participant was enrolled on October 5th, 2015 and the last participant was enrolled on November 7th, 2016 Data was collected between October 2015 and July 2017 at Granada University, Spain.
Outcomes	Primary endpoints: Change from Baseline in BAT volume and 18F-FDG uptake assessed by Positron emission tomography/computed tomography (PET/CT) [Time Frame: Baseline and 24-week later (immediately after the interventions ends)]<br>Secondary endpoints: changes in body composition (assessed by DEXA scan); changes in cardiometabolic risk factors assesed in serum and plasma and changes in physical fitness outcomes assessed by maximal effort test and handgrip test.
