# Supplementary material for: No evidence of brown adipose tissue activation after 24 weeks of supervised exercise training in young sedentary adults in the ACTIBATE randomized controlled trial
Source: Nat Commun. 2022 Sep 12;13:5259. doi: 10.1038/s41467-022-32502-x (PMC9467993; doi:10.1038/s41467-022-32502-x)
Supplement: Supplementary file 2 — Peer Review File [file 41467_2022_32502_MOESM2_ESM.pdf]

nature portfolio

Peer Review File

No evidence of brown adipose tissue activation after 24 weeks of supervised exercise training in young sedentary adults in the ACTIBATE randomized controlled trial

Open Access This file is licensed under a Creative Commons Attribution 4.0 International License, which permits use, sharing, adaptation, distribution and reproduction in any medium or format, as long as you give appropriate credit to the original author(s) and the source, provide a link to the Creative Commons license, and indicate if changes were made. In the cases where the authors are anonymous, such as is the case for the reports of anonymous peer reviewers, author attribution should be to 'Anonymous Referee' followed by a clear attribution to the source work. The images or other third party material in this file are included in the article's Creative Commons license, unless indicated otherwise in a credit line to the material. If material is not included in the article's Creative Commons license and your intended use is not permitted by statutory regulation or exceeds the permitted use, you will need to obtain permission directly from the copyright holder. To view a copy of this license, visit http://creativecommons.org/licenses/by/4.0/.

REVIEWER COMMENTS

Reviewer #1 (Remarks to the Author):

SUMMARY
Activation of brown adipose tissue (BAT) in humans via mild cold or pharmacologic stimulation has been fairly well established. The ability of exercise to stimulate BAT activity is well established in rodents; however, this concept of exercise-induced activation of BAT is fairly unexplored and remains inconclusive in humans. The current study executed a rigorous randomized, controlled supervised endurance and resistance exercise training intervention for 24 weeks in 145 young healthy sedentary adults to determine the impact of the training on BAT volume and glucose uptake via 18F-FDG-PET/CT, as well as other measures of adiposity, strength and cardiorespiratory fitness. Despite robust increases in VO2max and leg strength and significant loss of fat mass, in particular visceral adipose tissue, the authors found no changes in BAT volume nor BAT glucose uptake following 24 weeks of training. The authors noted that the time/day of the year in which the baseline 18F-FDG PET scans were performed was related to the response in BAT volume and glucose uptake. However, no relationships were observed among the changes in VO2max, adiposity and strength with the changes in BAT volume and BAT glucose uptake. While the findings are null, they are conclusive. These findings will make a significant contribution to this area of research by using the gold-standard technique of 18F-FDG PET to assess BAT activity and volume, in addition to performing such a rigorously controlled and supervised intervention in a large cohort. I have only minor comments that need to be addressed and are detailed below.

MINOR COMMENTS:
In agreement/support of the authors' findings, BAT glucose metabolism better reflects BAT's insulin sensitivity and blood flow. It is now clear that most glucose taken up by BAT does not fuel mitochondrial oxidative metabolism and that BAT glucose uptake can therefore be disconnected from thermogenesis…as would be the expected case with exercise training to increase oxidative metabolism rather than thermogenesis. That being said, while the authors use the currently accepted gold standard method of 18F-FDG PET for assessments of BAT activity and volume, they do not discuss the other established non-invasive methodologies that have been used to determine the contribution of triglycerides and fatty acids as a BAT fuel/substrate using 11C-acetate, as well as the use of 15O for oxygen consumption. Some references are provided below.

- PMID: 30919091
- PMID: 30131768
- PMID: 29802209
- PMID: 28134339
- PMID: 28089568
- PMID: 24423363
- PMID: 22269323

Figure 3 is intriguing in that it shows an inverse correlation between the time of year (natural calendar

day) for the baseline scan and the response to training in terms of BAAT volume and activity. This seems to align with the notion that the BAT uptake is higher in colder seasons. However, I wonder if the authors have correlated the baseline and the post-intervention PET scans with the respective days of the year on which they were measured rather than the change since the intervention took place over a period of 6 months and therefore the temperature outside was dramatically different during the baseline scan vs the post-intervention scan.

Further to this finding of the seasonal temperature variation being correlated with the changes in BAT volume and activity, have the authors adjusted for the "baseline scan day of the year" in their analyses of the exercise intervention responses in BAT volume and activity (ie Figure 2)?

In the Introduction (Lines 91-93) and in the Results/Discussion (Lines 242-245), the authors allude to the ability of exercise to "brown/beige" WAT in humans. Since there is currently no direct evidence of exercise-induced browning of WAT in humans, the authors should soften such speculation. Also related, the Reference #66 on Line 245 is incorrect. The following publications are among the very few (if not only) investigations into the effect of exercise training on browning in human WAT. These should be referenced and discussed.

- PMID: 27804230
- PMID: 26189600
- PMID: 24237962
- PMID: 30212302

Related to this topic of browning of human WAT in response to exercise training, the authors do measure and present gene expressions of 3 markers of brown adipose tissue (Table S1 – WAT Browning Markers). I recommend that these data should be removed as they are measured so far out from the last bout of exercise (10-11 days post last bout as stated by the authors) that they are not reflective of a chronic training response in WAT and thus inconclusive. Additionally, the 3 markers are not a very comprehensive assessment of "browning/beiging" as there are some key genes that have been omitted, such as CD137, PRDM16, TMEM26.

DETAILED ERRORS/TYPOS:

- Reference 11 in Line 79 is for a mouse study, not humans. The correct reference would be the paper by Wu J et al., 2012, Cell (PMID: 22796012).
- In the Study Subjects Section (lines 126-127), the text says to see criteria below, but there is no reference to Figure 1. Please add.
- Line 158 references a very recent study in mice demonstrating sexual dimorphism in training responses in WAT (reference #31). However, the authors state that this is in BAT, rather than WAT, and extrapolate the findings to human, which is over-reaching. Please remove/revise. Sex differences in BAT volume and activity have been demonstrated by other groups in response to mild cold and at thermoneutrality, and those should be referenced. (PMID: 25894250; PMID: 20357363; PMID: 31970907

to list a few, but there are more).

Reviewer #2 (Remarks to the Author):

Martinez-Tellez B and Sanchez-Delgado G et al investigated the effects of two-types of exercise interventions on brown adipose tissue (BAT) volume/activity in healthy young humans. This is a well-designed intervention study with a long-term experimental period and a huge number of volunteers. They found that BAT volume/activity was unaffected by the exercise regimens whereas they found a significant reduction of body fat and an increase in muscular fitness following the interventions. Their findings are important to the research field because it has remained controversial for a while if exercise regimens are capable of activating human BAT. I have only a few concerns to be addressed.

1) As this is a study with a long-term treatment period (24 weeks, ~a half year), I am wondering if BAT volume/activity assessed by FDG-PET/CT may be highly fluctuated during the experimental period due to significant intra-individual seasonal variations of BAT, which makes it difficult to detect effects of exercise on BAT. Thus, the authors should report outdoor temperature at the day when baseline PET/CT scan was performed and outdoor temperature at the day when final PET/CT scan was performed. The authors have shown the relationship between natural day of the year when baseline PET/CT was performed and delta-BAT activity/volume (Fig. 3A-D); however, these are not sufficient to argue that the effects of exercise are not masked by the changes in outdoor temperature during the experimental period.
2) The authors should also analyze the relationship between “delta-BAT activity/volume” and “delta-outdoor temperature” to prove that the observed results are not affected by the seasonal variation of BAT.
3) Another possible confounding factor is the accuracy and reproducibility of the FDG-PET/CT examination with a personalized cold exposure for assessing BAT activity/volume. In fact, delta-BAT volume shows hundreds mL of the changes in BAT volume even in the sedentary control group (Fig. 2A), suggesting a huge fluctuation of BAT volume independent of exercise effects. Thus, the authors should analyze correlation between BAT volume/activity baseline and those after the intervention. This analysis might support the highly reproducible assessment of BAT by the methods and then strengthen the conclusion of this study.
4) The parallel-group comparison study design is also a limitation of the study. The cross-over study design would be beneficial to investigate such potentially small effects on BAT because in the cross-over design, each subject acts as own control. Thus, the limitation and the need for retesting the effects of exercise on BAT in a season-matched cross-over design (see PMID: 28275131) should be described in the Discussion.

Reviewer #3 (Remarks to the Author):

Dear authors,

I reviewed the manuscript “A 24-week exercise intervention does not modify brown adipose tissue volume and 18F-FDG uptake in young sedentary adults” with great interest. I appreciate the thorough literature review and background, as well as the concise and clear writing style. As best I can tell, the study was conducted well and seeks to address an important question. I particularly like the figures, as many of them overlay the raw data. The underlying science is beyond the scope of my area of expertise; therefore, my major comments at this stage will mostly pertain to design, analysis, reporting, and interpretation of the results and I hope these critiques are useful to the authors in order to strengthen the manuscript.

1) Much of the information that I might expect from a manuscript reporting the results of a randomized controlled trial appears to be missing. For instance:

a. Some sort of information about institutional approval.
b. Information on how patients were recruited initially.
c. Information about power and sample size.
d. Detailed description of inclusion/exclusion criteria.
e. Information on how the randomization was performed. Was it stratified? Were there permuted blocks?
f. Information about statistical methodology (I had to dig to find the statistical methods in the table footnotes/figure legends, but I think detailed information about how the analyses were conducted is merited in the main body of the text).

2) Elaborating on comment 1(e) in particular, ANOVA and ANCOVA make unnecessary assumptions surrounding constant error variance. Moreover, these methods do not allow you to leverage baseline information by adjusting for covariates for increased power. Embedding analyses into mixed-model or a generalized estimating equations framework will strengthen the analysis greatly, especially if employing robust variance estimation methods. Further, it needs to be made clear that these methods are comparing differences in means.

3) Across the board, null statements are stated in a way that is too strong. The null hypothesis cannot be proven true but only falsified by the experimental data. This is particularly problematic since the confidence intervals were quite wide across the board (all the more reason a pre-specified power calculation should be presented). Here are some examples of statements that sound like they are proving the null hypothesis:

a. In the title: “…does not modify…”
b. In the abstract: “Neither of the exercise regimens modified…” and “were not related.”
c. In the results “Neither…modified BAT volume…”
d. etc.

Instead, language such as “there is not sufficient evidence of an effect/change…” etc. is to be preferred.

4) P-values in Table 1 are not appropriate. Since the experiment is randomized, all imbalances are random and not systematic (p-values are used to quantify statistical strength of evidence of systematic associations, not random imbalances). I would follow recent guidance on this by simply removing p-values for the baseline comparisons as they are meaningless.

5) I found myself a little bit confused by the organization of the results/discussion, as it seems that they are sort of interwoven. Thus is an editorial comment so I will let the editorial board weigh in, but in my experience, Nature papers tend to have a separate section for the discussion.

Best regards,

RESPONSE TO REVIEWERS' COMMENTS FOR MS. NCOMMS-21-37906

Reviewer #1
SUMMARY

Activation of brown adipose tissue (BAT) in humans via mild cold or pharmacologic stimulation has been fairly well established. The ability of exercise to stimulate BAT activity is well established in rodents; however, this concept of exercise-induced activation of BAT is fairly unexplored and remains inconclusive in humans. The current study executed a rigorous randomized, controlled supervised endurance and resistance exercise training intervention for 24 weeks in 145 young healthy sedentary adults to determine the impact of the training on BAT volume and glucose uptake via ^{18}F-FDG-PET/CT, as well as other measures of adiposity, strength and cardiorespiratory fitness. Despite robust increases in VO_2max and leg strength and significant loss of fat mass, in particular visceral adipose tissue, the authors found no changes in BAT volume nor BAT glucose uptake following 24 weeks of training. The authors noted that the time/day of the year in which the baseline ^{18}F-FDG PET scans were performed was related to the response in BAT volume and glucose uptake. However, no relationships were observed among the changes in VO_2max, adiposity and strength with the changes in BAT volume and BAT glucose uptake. While the findings are null, they are conclusive. These findings will make a significant contribution to this area of research by using the gold-standard technique of ^{18}F-FDG PET to assess BAT activity and volume, in addition to performing such a rigorously controlled and supervised intervention in a large cohort. I have only minor comments that need to be addressed and are detailed below.

MINOR COMMENTS:

Comment 1: In agreement/support of the authors' findings, BAT glucose metabolism better reflects BAT's insulin sensitivity and blood flow. It is now clear that most glucose taken up by BAT does not fuel mitochondrial oxidative metabolism and that BAT glucose uptake can therefore be disconnected from thermogenesis…as would be the expected case with exercise training to increase oxidative metabolism rather than thermogenesis. That being said, while the authors use the currently accepted gold standard method of ^{18}F-FDG PET for assessments of BAT activity and volume, they do not discuss the other established non-invasive methodologies that have been used to determine the contribution of triglycerides and fatty acids as a BAT fuel/substrate using ^{11}C-acetate, as well as the use of ^{15}O for oxygen consumption. Some references are provided below.

- PMID: 30919091
- PMID: 30131768
- PMID: 29802209
- PMID: 28134339
- PMID: 28089568
- PMID: 24423363
- PMID: 22269323

> **Answer 1:** We do appreciate the reviewer's comments and suggestions. We have included this information, as well as some references on page 11 line 240: *"…since most glucose taken up by BAT does not fuel mitochondrial oxidative metabolism."* and page 11 lines 251-258: *"Moreover, exercise training increases mitochondrial oxidative capacity in skeletal muscle, and it might likewise increase BAT oxidative metabolism rather than BAT ^{18}F-FDG uptake. BAT oxidative metabolism, as well as the contribution of fatty acids in human BAT, has been recently quantified by ^{11}C-acetate,*

^{15}O-oxygen and ^{18}F-fluoro-thiaheptadecanoic acid (^{18}FTHA) PET in response to cold exposure in humans. Thus, it would be interesting to ascertain in future studies if assessing BAT metabolism by methods other than glucose uptake would unveil whether exercise can activate/recruit BAT."

Comment 2: Figure 3 is intriguing in that it shows an inverse correlation between the time of year (natural calendar day) for the baseline scan and the response to training in terms of BAT volume and activity. This seems to align with the notion that the BAT uptake is higher in colder seasons. However, I wonder if the authors have correlated the baseline and the post-intervention PET scans with the respective days of the year on which they were measured rather than the change since the intervention took place over a period of 6 months and therefore the temperature outside was dramatically different during the baseline scan vs the post-intervention scan.

Answer 2: We appreciate this comment. We have analysed the correlation between the baseline and the post-intervention BAT-related outcomes with the respective natural calendar days at which the PET/CT scans were performed. We found that the natural calendar days at which baseline PET/CT scans were performed positively correlated with BAT volume and BAT ^{18}F-FDG uptake (see new **Table S2** below), whereas no correlation was observed between the natural calendar days at which the post-intervention PET/CT scans were performed and any BAT-related outcomes (**Table S2**). These findings persisted when using outdoor ambient temperature instead of the natural calendar day when the post-intervention PET/CT scans were performed.

We have added a section in the results of the manuscript describing this finding, see page 9 lines 199-202: *"At baseline, we found that natural calendar days at which the PET/CT scans were performed positively correlated with BAT-related outcomes (all $r \geq 0.422$ and $P<0.001$, Table S2), although these significant correlations disappeared when post-intervention values were used instead (Table S2*; see supplementary material page 4*)."*

New Table S2. Pearson correlations between natural calendar day when the baseline or post-intervention positron emission tomography/computed tomography (PET/CT) scans were performed with baseline, or post-intervention, brown adipose tissue (BAT)-related outcomes.

	BAT VOLUME		BAT SUVmean		BAT SUVpeak		BAT mean radiodensity	
**BASELINE**<br>All sample (n=97)								
	r	P	r	P	r	P	r	P
Natural calendar day when baseline PET/CT scans were performed	0.484	**<0.001**	0.422	**<0.001**	0.446	**<0.001**	0.176	0.150
**POST-INTERVENTION**<br>All sample (n=97)								
	BAT VOLUME		BAT SUVmean		BAT SUVpeak		BAT mean radiodensity	
	r	P	r	P	r	P	r	P
Natural calendar day when post-intervention PET/CT scans were performed	-0.047	0.646	-0.076	0.462	-0.097	0.350	-0.14	0.229
CON (N=35)								
	r	P	r	P	r	P	r	P
Natural calendar day when post-intervention PET/CT scans were performed	-0.117	0.505	-0.070	0.689	-0.144	0.408	-0.331	0.080
MOD-EX (N=31)								
	r	P	r	P	r	P	r	P
Natural calendar day when post-intervention PET/CT scans were performed	0.039	0.837	-0.129	0.496	-0.169	0.372	0.122	0.571
VIG-EX (N=31)								
	r	P	r	P	r	P	r	P
Natural calendar day when post-intervention PET/CT scans were performed	-0.044	0.815	-0.014	0.940	0.001	0.994	-0.110	0.619

CON: control group; MOD-EX: moderate-intensity exercise group; VIG-EX: vigorous-intensity exercise group.

Comment 3: Further to this finding of the seasonal temperature variation being correlated with the changes in BAT volume and activity, have the authors adjusted for the “baseline scan day of

the year" in their analyses of the exercise intervention responses in BAT volume and activity (ie Figure 2)?

Answer 3: Although the natural calendar day of the PET/CT scans influenced the estimation of BAT-related variables, the participants were randomly allocated to the three groups. We did not observe group differences in the natural calendar day when the PET/CT scans were performed, and hence we decided not to adjust the analyses for this variable. Nonetheless, following the reviewer's comment, we repeated the analysis after adjusting for the natural calendar day when the baseline PET/CT scans were performed and the results did not change (See new Table S3 below; supplementary material page 5). We have decided to add this table as supplementary Table S3 and mention this finding on page 9 lines 202-205: *"After observing these correlations, we performed the main analyses adding the natural calendar day when the baseline PET/CT scans were performed as covariate and the results did not change (all P≥0.093, Table S3)."*

New Table S3. Effect of the 24-week supervised exercise intervention on brown adipose tissue (BAT) volume, ^{18}F-FDG uptake (Standardized uptake Value [SUV] mean and peak), and mean radiodensity.

Outcome	P-value
Δ BAT volume (mL)	0.747
Δ BAT SUVmean	0.906
Δ BAT SUVpeak	0.343
Δ Total BAT mean radiodensity (HU)	0.093

Δ was calculated as post-intervention minus baseline values for every outcome. P values are from analyses of covariance adjusting for baseline values and natural calendar day when the baseline PET/CT scans were performed (n=97). Total BAT mean radiodensity (control group n=25; Moderate-intensity exercise group n=17; Vigorous-intensity exercise group n=16).

Comment 4: In the Introduction (Lines 91-93) and in the Results/Discussion (Lines 242-245), the authors allude to the ability of exercise to "brown/beige" WAT in humans. Since there is currently no direct evidence of exercise-induced browning of WAT in humans, the authors should soften such speculation. Also related, the Reference #66 on Line 245 is incorrect. The following publications are among the very few (if not only) investigations into the effect of exercise training on browning in human WAT. These should be referenced and discussed.
• PMID: 27804230
• PMID: 26189600
• PMID: 24237962
• PMID: 30212302

Answer 4: Following the reviewer's comments we have softened this speculation in the introduction and discussion:

Introduction, page 4, lines 90-93: *"Recruiting or activating BAT and WAT browning might therefore have cardiometabolic health benefits. Mouse studies have shown that exercise induces WAT browning, and exercise might thus be speculated to do the same in humans".*

Results: Since we have rewritten the sentence, and adapted the manuscript, the reference was no longer needed it. See page 12 lines 266-269: *"Thus, exercise might not increase the volume or activity of the brown-like depots detectable by PET/CT scanning, but may still induce the browning of individual adipocytes, that are not detected by this technique.".*

Comment 5: Related to this topic of browning of human WAT in response to exercise training, the authors do measure and present gene expressions of 3 markers of brown adipose tissue (Table S1 – WAT Browning Markers). I recommend that these data should be removed as they are measured so far out from the last bout of exercise (10-11 days post last bout as stated by the authors) that they are not reflective of a chronic training response in WAT and thus inconclusive. Additionally, the 3 markers are not a very comprehensive assessment of "browning/beiging" as there are some key genes that have been omitted, such as CD137, PRDM16, TMEM26.

> **Answer 5:** We agree with the reviewer and we have therefore completely removed gene expression analyses, description of the results, and procedures of the WAT biopsies from the manuscript.

Comment 6: DETAILED ERRORS/TYPOS:
• Reference 11 in Line 79 is for a mouse study, not humans. The correct reference would be the paper by Wu J et al., 2012, Cell (PMID: 22796012).

Answer 6: Thanks for realizing of this mistake. The reference has been corrected (see page 4, line 80).

Comment 7:
• In the Study Subjects Section (lines 126-127), the text says to see criteria below, but there is no reference to Figure 1. Please add.

Answer 7: Thanks, the reference to Figure 1 has been added (see page 6, line 126).

Comment 8:

• Line 158 references a very recent study in mice demonstrating sexual dimorphism in training responses in WAT (reference #31). However, the authors state that this is in BAT, rather than WAT, and extrapolate the findings to human, which is over-reaching. Please remove/revise. Sex differences in BAT volume and activity have been demonstrated by other groups in response to mild cold and at thermoneutrality, and those should be referenced. (PMID: 25894250; PMID: 20357363; PMID: 31970907 to list a few, but there are more).

> **Answer 8:** Thanks, we have adapted this paragraph accordingly and added the references suggested. See page 8, lines 159-163*: "Previous clinical studies have shown that BAT volume and ^{18}F-FDG uptake in response to mild-cold and thermoneutral exposures are different in women and men, which was also confirmed in the cold-induced BAT of this cohort. However, the absence of evidence of BAT activation after exercise training was recorded for both sexes when analysed separately*".

Reviewer #2
Martinez-Tellez B and Sanchez-Delgado G et al investigated the effects of two-types of exercise interventions on brown adipose tissue (BAT) volume/activity in healthy young humans. This is a well-designed intervention study with a long-term experimental period and a huge number of volunteers. They found that BAT volume/activity was unaffected by the exercise regimens whereas they found a significant reduction of body fat and an increase in muscular fitness following the interventions. Their findings are important to the research field because it has remained controversial for a while if exercise regimens are capable of activating human BAT. I have only a few concerns to be addressed.

Comment 1: As this is a study with a long-term treatment period (24 weeks, ~a half year), I am wondering if BAT volume/activity assessed by FDG-PET/CT may be highly fluctuated during the experimental period due to significant intra-individual seasonal variations of BAT, which makes it difficult to detect effects of exercise on BAT. Thus, the authors should report outdoor temperature at the day when baseline PET/CT scan was performed and outdoor temperature at the day when final PET/CT scan was performed. The authors have shown the relationship between natural day of the year when baseline PET/CT was performed and delta-BAT activity/volume (Fig. 3A-D); however, these are not sufficient to argue that the effects of exercise are not masked by the changes in outdoor temperature during the experimental period.

> **Answer 1**: We do agree with the reviewer's comment, and have included the outdoor temperature registered by the Spanish agency of the meteorology in the manuscript, see Methods section on page 20 lines 454-457: "*We downloaded the outdoor ambient temperature from the Spanish National Meteorological Agency daily during the period of the study (from September 1st, 2015 to July 1st, 2017)*". Moreover, we have added this information in Table S1 (see supplementary material, page 3). Altogether, we believe that these results suggest that the effect of exercise on BAT activity might be masked by the seasonal variation.

Comment 2: The authors should also analyze the relationship between "delta-BAT activity/volume" and "delta-outdoor temperature" to prove that the observed results are not affected by the seasonal variation of BAT.

> **Answer 2:** Following the reviewer's comment, we have analyzed the relationship between "delta-BAT activity/volume" and "delta-outdoor temperature", finding similar results than in Figures 3A-D. Accordingly, we have included these findings as Figure S4 (see below and supplementary material page 12) and added this finding in the results section see page 9 lines 195-199: "*The same significant correlations were observed when the baseline ambient outdoor temperature or Δ outdoor ambient temperature were included instead of the natural calendar days at which the baseline PET/CT scans were performed (Figure S4) (i.e., the higher the outdoor temperature, the lower the BAT volume and activity)*"

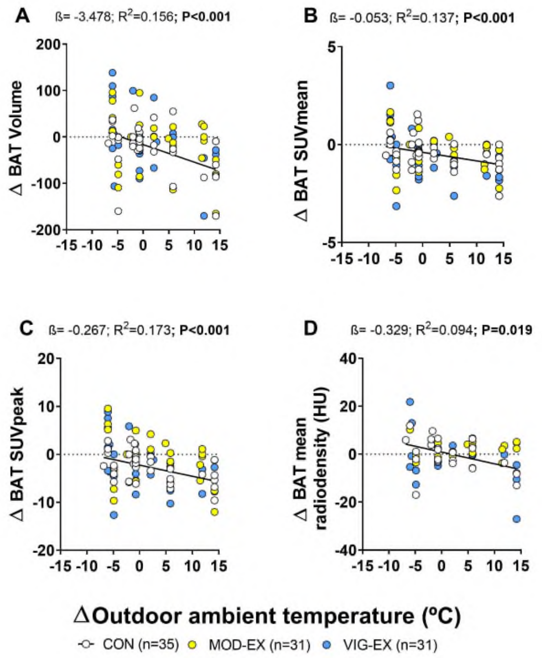

New Figure S4: Associations between Δ (post-intervention minus baseline value) outdoor ambient temperature and Δ brown adipose tissue (BAT)-related outcomes. A. Total BAT volume. B. Total BAT standardized uptake value (SUV) mean. C. Total BAT SUVpeak. D. Total BAT mean radiodensity (CON n=25; MOD-EX n=17; VIG-EX n=16). SUV values are shown relative to lean mass. β: non-standardized coefficients; BAT: brown adipose tissue; CON: Control group; HU: Hounsfield units; MOD-EX: Moderate-intensity exercise group; R2: explained variance; SUV: standardized uptake value; VIG-EX: Vigorous-intensity exercise group.

Comment 3: Another possible confounding factor is the accuracy and reproducibility of the FDG-PET/CT examination with a personalized cold exposure for assessing BAT activity/volume. In fact, delta-BAT volume shows hundreds mL of the changes in BAT volume even in the sedentary control group (Fig. 2A), suggesting a huge fluctuation of BAT volume independent of exercise effects. Thus, the authors should analyze correlation between BAT volume/activity baseline and those after the intervention. This analysis might support the highly reproducible assessment of BAT by the methods and then strengthen the conclusion of this study.

Answer 3:

We appreciate the reviewer's comment. The reproducibility of BAT-related variables measured by ^{18}F-FDG-PET/CT scan after a personalized cooling protocol is high, as recently demonstrated[1]. However, the reviewer is right that the long time elapsed between the two assessments in our study (and the associated seasonal changes) might have reduced such reproducibility. Following the reviewer's suggestion, we have analysed the association between the baseline and post-intervention BAT-related outcomes. New Table S4 shows that the correlations between baseline and post-intervention (final) BAT-related outcomes are moderate (~all r=0.5), see below. Table S4 is included in the supplemental material of the manuscript (page 6) and these results are discussed on page 9 lines 205-208: "*In addition, we found weak to strong correlations (range from r=0.346 to r=0.878) between baseline and post-intervention BAT related-outcomes, which suggests that our protocols were reproducible despite the presence of large seasonal variation (Table S4).*"

New Table S4: Partial correlations between baseline and post-intervention brown adipose tissue (BAT)-related outcomes adjusted for the natural calendar day when the baseline positron emission tomography/computed tomography scans were performed.

		**POST INTERVENTION**							
		BAT Volume		BAT SUVmean		BAT SUVpeak		BAT mean radiodensity	
CON (n=35)	**BASELINE**	r	P	r	P	r	P	r	P
	BAT Volume	0.521	**0.002**	-	-	-	-	-	-
	BAT SUVmean	-	-	0.746	**<0.001**	-	-	-	-
	BAT SUVpeak	-	-	-	-	0.745	**<0.001**	-	-
	BAT mean radiodensity	-	-	-	-	-	-	0.833	**<0.001**

MOD-EX (n=31)	BAT Volume	0.346	0.061	-	-	-	-	-	-
	BAT SUVmean	-	-	0.626	**<0.001**	-	-	-	-
	BAT SUVpeak	-	-	-	-	0.528	**0.003**	-	-
	BAT mean radiodensity	-	-	-	-	-	-	0.878	**<0.001**
VIG-EX (n=31)	BAT Volume	0.413	**0.023**	-	-	-	-	-	-
	BAT SUVmean	-	-	0.401	**0.028**	-	-	-	-
	BAT SUVpeak	-	-	-		0.478	**0.008**	-	-
	BAT mean radiodensity	-	-	-	-	-	-	0.321	0.243

CON: control group; MOD-EX: Moderate-intensity exercise group; VIG-EX: Vigorous-intensity exercise group.

Comment 4: The parallel-group comparison study design is also a limitation of the study. The cross-over study design would be beneficial to investigate such potentially small effects on BAT because in the cross-over design, each subject acts as own control. Thus, the limitation and the need for retesting the effects of exercise on BAT in a season-matched cross-over design (see PMID: 28275131) should be described in the Discussion.

Answer 4: We fully agree with the reviewer's comment on that using a crossover study design would be an excellent way to exclude the seasonal variation in short-term interventions. When testing the effect of relatively long exercise training trials, as the ACTIBATE (24-week exercise intervention), crossover study designs are hardly feasible. A potential way to, somehow, control for the seasonal variation would be to conduct a 1-year exercise intervention study with a control group and with multiple BAT measurements. Nonetheless, following the reviewer's comment, we have included this issue as limitation: "*Likewise, other study designs and experimental settings, with longer durations, should be implemented for addressing this scientific question in the future.*" (See page 12 lines 279-281).

Reviewer #3

Dear authors, I reviewed the manuscript "A 24-week exercise intervention does not modify brown adipose tissue volume and 18F-FDG uptake in young sedentary adults" with great interest. I appreciate the thorough literature review and background, as well as the concise and clear writing style. As best I can tell, the study was conducted well and seeks to address an important question. I particularly like the figures, as many of them overlay the raw data. The underlying science is beyond the scope of my area of expertise; therefore, my major comments at this stage will mostly pertain to design, analysis, reporting, and interpretation of the results and I hope these critiques are useful to the authors in order to strengthen the manuscript.

Comment 1: Much of the information that I might expect from a manuscript reporting the results of a randomized controlled trial appears to be missing. For instance:

a. Some sort of information about institutional approval.
b. Information on how patients were recruited initially.
c. Information about power and sample size.
d. Detailed description of inclusion/exclusion criteria.
e. Information on how the randomization was performed. Was it stratified? Were there permuted blocks?
f. Information about statistical methodology (I had to dig to find the statistical methods in the table footnotes/figure legends, but I think detailed information about how the analyses were conducted is merited in the main body of the text).

Answer 1: We believe that the reviewer may have missed this information, as it was included in the PDF following the references (pages 20-31 of the original document). Please, find below the information:

a. Page 16 lines 359-362: "*The study was approved by the Ethics Committee on Human Research of the University of Granada (no. 924) and by the Servicio Andaluz de Salud (Centro de Granada, CEI-Granada, Spain). All subjects provided informed consent in order to be included.*"
b. Page 16 lines 350-351: *"For subject recruitment, the study was advertised through social networks, local media, and via posters."*
c. Page 23 lines 537-547: "*A conservative approach to sample size estimation was followed, and a relatively large standard deviation assumed based on the heterogeneity of the data published on humans up to the time when the study was designed (March 2014). Increases of 10% and 20% were anticipated in activated BAT volume at 24 weeks in the MOD-EX and VIG-EX groups respectively (rising from a baseline level of 50-70 mL), along with a standard deviation of 50-60 mL. Assuming an effect in either direction, differences of at least 10% in BAT volume could be detected with a power of >80% and an α of 0.05 in a group of 17 subjects per study group. To study sex differences, a total of 34 subjects (17 men and 17 women) were required for each group. Assuming a maximum loss to follow-up of 30%, 150 subjects were thus targeted (i.e., 50 per group). The IBM-SPSS Sample power software (version 3.0.1) was used for calculations.*"
d. Page 16 lines 351-355: "*The inclusion criteria included a self-reported sedentary lifestyle (performing a maximum of 20 min of moderate-to-vigorous physical activity per day on less than three days per week), being a non-smoker, not taking any medication, and having a stable body weight over the last three months. The exclusion criteria were: having been diagnosed with diabetes, hypertension, or any other significant medical condition (either life-threatening or that might interfere with or be aggravated by exercise), being pregnant, using medication deemed to affect energy metabolism, or to be frequently exposed to cold temperatures.*"
e. Page 16 lines 364-367: "*After baseline examination, subjects were assigned to one of three groups via computer-generated simple unrestricted randomization by an external investigator: (i) a control group (no exercise, CON), (ii) a moderate-intensity exercise group (MOD-EX), and (iii) a vigorous-intensity exercise group (VIG-EX).*"
f. We have a dedicated section where we carefully explained all the statistical analyses applied. See page 24, section *Quantification and statistical analysis*

Comment 2: Elaborating on comment 1(e) in particular, ANOVA and ANCOVA make unnecessary assumptions surrounding constant error variance. Moreover, these methods do not allow you to leverage baseline information by adjusting for covariates for increased power. Embedding analyses into mixed-model or a generalized estimating equations framework will

strengthen the analysis greatly, especially if employing robust variance estimation methods. Further, it needs to be made clear that these methods are comparing differences in means.

Answer 2: The statistical approach used for this manuscript has been an important point of discussion in the group because we wanted to find the simplest yet most appropriate way to present the results for a broad readership when published.

We agree with the reviewer on that ANOVA/ANCOVA make unnecessary assumptions; however, we performed Levene's test to investigate whether the variances were equal in the primary outcomes (see **Table R1**). As it can be observed in Table R1, most of the primary outcomes show similar variances, which thus rectifies the application of ANOVA or ANCOVA.

Table R1: Levene's test of equality of error variances for the primary outcomes

	**F(2,94)**	**P**
Δ BAT volume (mL)	2.4	0.093
Δ BAT SUVmean	2.6	0.073
Δ BAT SUVpeak	4.9	**0.009**
Δ BAT mean radiodensity (HU)	0.9	0.379
**Δ Volume (mL)**		
Left BAT laterocervical	0.03	0.970
Right BAT laterocervical	0.9	0.414
Left BAT supraclavicular	2.2	0.121
Right BAT supraclavicular	2.3	0.098
BAT mediastinum	0.8	0.434
BAT paravertebral	0.8	0.437
**Δ SUVMean**		
Left BAT laterocervical	3.0	0.053
Right BAT laterocervical	2.3	0.099
Left BAT supraclavicular	3.2	**0.044**
Right BAT supraclavicular	2.7	0.070
BAT mediastinum	0.5	0.594
BAT paravertebral	1.1	0.312
**Δ SUVpeak**		
Left BAT laterocervical	4.4	**0.014**
Right BAT laterocervical	3.5	**0.034**
Left BAT supraclavicular	3.2	**0.044**
Right BAT supraclavicular	4.5	**0.013**
BAT mediastinum	1.7	0.179
BAT paravertebral	2.1	0.125

P<0.05 means that variances are not equal

Nevertheless, as suggested by the reviewer, we performed mixed-effects regression models with random intercepts to test the primary hypothesis presented in Figures 2A, B, C and D. New figure S2 (see also below, see supplementary material page 10) shows that the results are similar to those obtained with the ANOVAs/ANCOVAs, and thus, the main message from the manuscript does not change. We have included these analyses as supplementary figure S2 and a sentence in the results section see page 7, lines 155-157: "*We repeated the analyses using mixed-effects regression models and the absence of evidence of BAT activation after supervised exercise training persisted (Figure S2,* see supplementary material, page 10)."

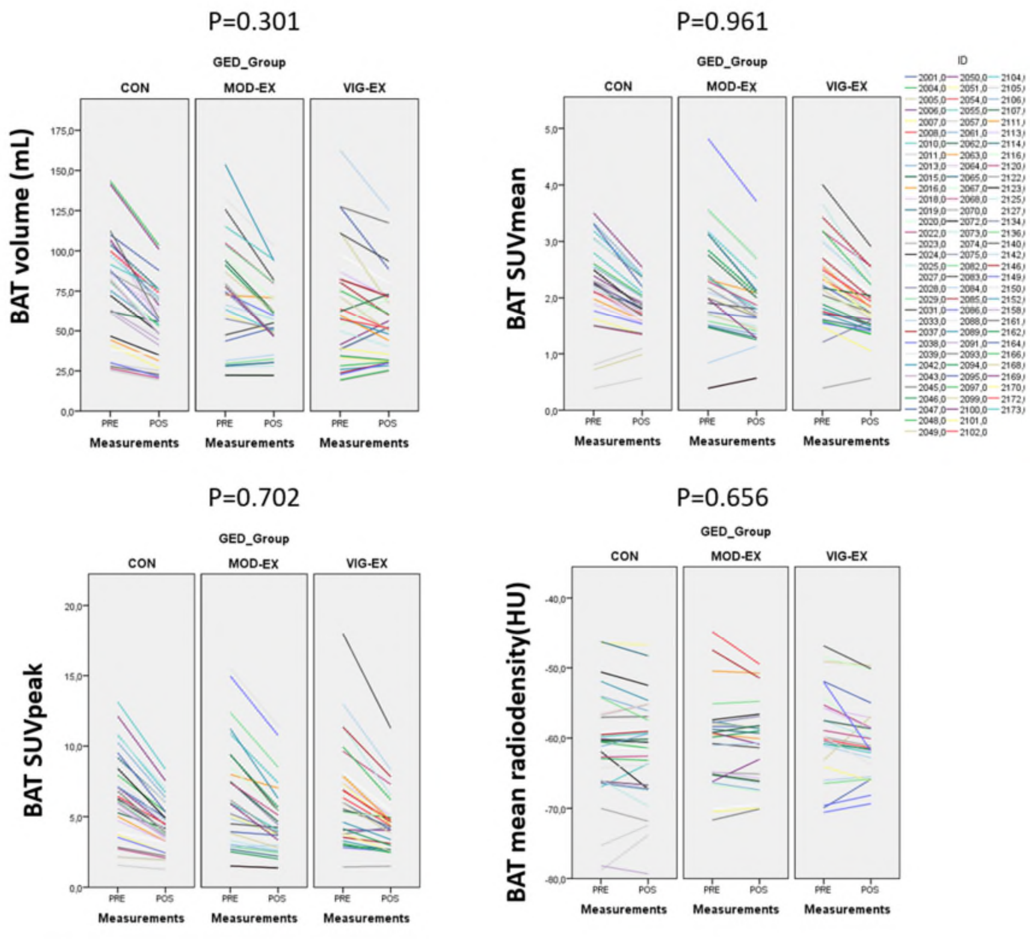

New Figure S2: No evidence of brown adipose tissue activation after 24 weeks of supervised exercise training in young sedentary adults when using mixed-effects regression models. BAT: brown adipose tissue; CON: Control; HU: Hounsfield Units; ID: identification code of the participant. MOD-EX: moderate exercise intensity; PRE: before starting exercise training measurements. POS: post-intervention measurements; VIG-EX: vigorous exercise intensity.

Comment 3: Across the board, null statements are stated in a way that is too strong. The null hypothesis cannot be proven true but only falsified by the experimental data. This is particularly problematic since the confidence intervals were quite wide across the board (all the more reason a pre-specified power calculation should be presented). Here are some examples of statements that sound like they are proving the null hypothesis:

a. In the title: "…does not modify…"
b. In the abstract: "Neither of the exercise regimens modified…" and "were not related."
c. In the results "Neither…modified BAT volume…"
d. etc.

Instead, language such as "there is not sufficient evidence of an effect/change…" etc. is to be preferred.

Answer 3: We value the reviewer's comments, and have carefully changed the manuscript accordingly. Following the reviewer's comment, we have also changed the title: "*No evidence of brown adipose tissue activation after 24 weeks of exercise training in young sedentary adults*". Moreover, we have highlighted the power size calculation performed in this study, see previous comments or page 23 lines 537-547 in the manuscript.

Comment 4: P-values in Table 1 are not appropriate. Since the experiment is randomized, all imbalances are random and not systematic (p-values are used to quantify statistical strength of evidence of systematic associations, not random imbalances). I would follow recent guidance on this by simply removing p-values for the baseline comparisons as they are meaningless.

Answer 4: Following the reviewer's comment, P-values have been removed from Table 1 (see main manuscript page 34) and Table S1 (see supplementary material pages 2 and 3).

Comment 5: I found myself a little bit confused by the organization of the results/discussion, as it seems that they are sort of interwoven. Thus is an editorial comment so I will let the editorial board weigh in, but in my experience, Nature papers tend to have a separate section for the discussion.

Answer 5: We present the Results and Discussion sections combined because we believe that it improves the manuscript presentation. Some analyses are justified based on previous literature and this is why we believe that this approach is more appropriate to present the findings in a more integrated way. Nevertheless, we have asked the Editor Dr. Riikka Jokinen about the possibility of keeping the results/discussion sections combined, and she said it can be editorially approved.

REFERENCES

1. Fraum, T. J. *et al.* Repeatability of Quantitative Brown Adipose Tissue Imaging Metrics on Positron Emission Tomography with 18F-Fluorodeoxyglucose in Humans. *Cell Metabolism* **30**, 212-224.e4 (2019).

REVIEWER COMMENTS

Reviewer #1 (Remarks to the Author):

The authors have done a very good job of addressing all of my comments and concerns. No further comments from me.

Reviewer #2 (Remarks to the Author):

In the revised manuscript, the authors have addressed most of my original comments and improved the manuscript. The authors observed negative correlations between changes in outdoor temperature and exercise-induced change in BAT activity, suggesting that the results of the current study might be masked by difference in seasons of experimental periods between individuals. However, the authors are still using the natural calendar days as an index of season which should be revised.

Answer 1: We do agree with the reviewer's comment, and have included the outdoor temperature registered by the Spanish agency of the meteorology in the manuscript, see Methods section on page 20 lines 454-457: "We downloaded the outdoor ambient temperature from the Spanish National Meteorological Agency daily during the period of the study (from September 1st, 2015 to July 1st, 2017)". Moreover, we have added this information in Table S1 (see supplementary material, page 3). Altogether, we believe that these results suggest that the effect of exercise on BAT activity might be masked by the seasonal variation.
Comment 1-2: Thanks for the additional analysis of outdoor temperature. As the authors said, seasonal changes in BAT activity would mask the effect of exercise on BAT. This limitation as well as a need for future study with a season-matched study design should be clearly described in the manuscript.

Answer 2: Following the reviewer's comment, we have analyzed the relationship between "delta-BAT activity/volume" and "delta-outdoor temperature", finding similar results than in Figures 3A-D. Accordingly, we have included these findings as Figure S4 (see below and supplementary material page 12) and added this finding in the results section see page 9 lines 195-199: "The same significant correlations were observed when the baseline ambient outdoor temperature or Δ outdoor ambient temperature were included instead of the natural calendar days at which the baseline PET/CT scans were performed (Figure S4) (i.e., the higher the outdoor temperature, the lower the BAT volume and activity)".
Comment 2-2: Outdoor temperature is a reliable index of season and ambient temperature. On the other hand, natural calendar day is not suitable for examining relationship between exercise-induced BAT activation and season. The use of natural calendar days is odd and not acceptable. New correlation analysis clearly suggests that outdoor temperature is a confounding factor for exercise-induced BAT activation. Therefore, the authors should use delta-outdoor temperature, instead of natural calendar days (Fig 3A-D). Moreover, the authors might use multivariate regression models, including outdoor temperature as a covariate, to examine effects of exercise on BAT.

Reviewer #3 (Remarks to the Author):

I appreciate the authors' response to these comments. Regarding my first comment, I should have clarified that my expectation was that this information would appear in the main body as part of the methodology, as this much of this seems to be essential. However, if the editors consider it adequate to keep this material following the references, that is fine. Thanks for the thoughtfulness of your responses.

Reviewer #1 (Remarks to the Author):

The authors have done a very good job of addressing all of my comments and concerns. No further comments from me.

Answer 1: We thank the Reviewer very much for the time invested in reviewing our work.

Reviewer #2 (Remarks to the Author):

In the revised manuscript, the authors have addressed most of my original comments and improved the manuscript. The authors observed negative correlations between changes in outdoor temperature and exercise-induced change in BAT activity, suggesting that the results of the current study might be masked by difference in seasons of experimental periods between individuals. However, the authors are still using the natural calendar days as an index of season which should be revised.

Comment 1-2: Thanks for the additional analysis of outdoor temperature. As the authors said, seasonal changes in BAT activity would mask the effect of exercise on BAT. This limitation as well as a need for future study with a season-matched study design should be clearly described in the manuscript.

Answer 1-2: Comment appreciated. The Reviewer's suggestions have been included in page 12 lines 274-279, see: *"Interestingly, we found that outdoor ambient temperature influenced BAT volume, ^{18}F-FDG uptake and radiodensity, which might be masking the possible effect of exercise on BAT recruitment/activity. Thus, other study designs and experimental settings, with longer durations, and taking into account the potential effect of outdoor ambient temperature, should be implemented for addressing this scientific question".*

Comment 2-2: Outdoor temperature is a reliable index of season and ambient temperature. On the other hand, natural calendar day is not suitable for examining relationship between exercise-induced BAT activation and season. The use of natural calendar days is odd and not acceptable. New correlation analysis clearly suggests that outdoor temperature is a confounding factor for exercise-induced BAT activation. Therefore, the authors should use delta-outdoor temperature, instead of natural calendar days (Fig 3A-D). Moreover, the authors might use multivariate regression models, including outdoor temperature as a covariate, to examine effects of exercise on BAT.

Answer 2-2: We agree with the Reviewer's comment on that outdoor temperature is a reliable index of season and ambient temperature. Therefore, following the Reviewer's suggestion, we have replaced in all the analyses the "natural calendar day" by the "outdoor ambient temperature", see new figure 3A-E and new tables S2, S3, S4 and S5. As previously suggested by this Reviewer, we have investigated the effect of exercise on BAT-related outcomes adjusting for outdoor ambient temperature and the lack of effect remained (see table S3). The lack of effect was similar after applying analyses of covariance or multivariate regression models.

Table S3. Effect of the 24-week supervised exercise intervention on brown adipose tissue (BAT) volume, 18F-fluorodeoxyglucose (^{18}F-FDG) uptake (Standardized uptake Value [SUV] mean and peak), and mean radiodensity.

Outcomes	P-value
**Model 1**	
Δ BAT volume (mL)	0.782
Δ BAT SUVmean	0.961
Δ BAT SUVpeak	0.433
Δ Total BAT mean radiodensity (HU)	0.182
**Model 2**	
Δ BAT volume (mL)	0.789
Δ BAT SUVmean	0.929
Δ BAT SUVpeak	0.332
Δ Total BAT mean radiodensity (HU)	0.815

Δ was calculated as post-intervention minus baseline values for every outcome. Model 1; P values are from analyses of covariance adjusting for baseline values and baseline outdoor ambient temperature (n=97). Model 2; P values are from analyses of covariance adjusting for baseline values and Δ outdoor ambient temperature (n=97) Total BAT mean radiodensity (control group n=25; Moderate-intensity exercise group n=17; Vigorous-intensity exercise group n=16).

Reviewer #3 (Remarks to the Author):

I appreciate the authors' response to these comments. Regarding my first comment, I should have clarified that my expectation was that this information would appear in the main body as part of the methodology, as this much of this seems to be essential. However, if the editors consider it adequate to keep this material following the references, that is fine. Thanks for the thoughtfulness of your responses.

Answer 1: We thank the Reviewer you very much for the time invested in reviewing our work.

REVIEWERS' COMMENTS

Reviewer #2 (Remarks to the Author):

In the revised manuscript, the authors have addressed all of my original comments and the manuscript has been improved substantially. I think it is suitable for publication in the current form.
